# Supplementary material for: Prevalence and short-term change in symptoms of anxiety and depression following bariatric surgery: a prospective cohort study
Source: BMJ Open. 2024 Jan 3;14(1):e071231. doi: 10.1136/bmjopen-2022-071231 (PMC10773381; doi:10.1136/bmjopen-2022-071231)
Supplement: Supplementary data [file bmjopen-2022-071231supp001.pdf]

Supplementary Results

Anxiety and depression following bariatric surgery

List of supplementary tables

		Page
Table 1	Change in clinical cases from baseline to 12 months post-randomisation	2
Table 2	Baseline characteristics by repeat HADS questionnaire return status	3
Table 3	Baseline characteristics by repeat HADS-A questionnaire return status	4
Table 4	Baseline characteristics by repeat HADS-D questionnaire return status	5

. *BMJ Open* 2024; 14:e071231. doi: 10.1136/bmjopen-2022-071231

Supplementary Results

Anxiety and depression following bariatric surgery

Table 1
Change in clinical cases from baseline to 12 months post-randomisation

		**Frequency**	**Proportion** (%)	**95% CI (%)**
**Anxiety** HADS-A (*n* 503)	Non-case unchanged	229	45.5	41.2 – 49.9
	Non-case to case	46	9.2	6.9 – 12.0
	Case to non-case	94	18.7	15.5 – 22.3
	Case unchanged	134	26.6	23.0 – 30.7
**Depression** HADS-D (*n* 498)	Non-case unchanged	245	49.2	44.8 – 53.6
	Non-case to case	22	4.4	2.9 – 6.6
	Case to non-case	133	26.7	23.0 – 30.8
	Case unchanged	98	19.7	16.4 – 23.4

. *BMJ Open* 2024; 14:e071231. doi: 10.1136/bmjopen-2022-071231

Supplementary Results

Anxiety and depression following bariatric surgery

Table 2
Participant baseline characteristics by repeat HADS questionnaire return status
(Continuous variables)

		12 months post-randomisation HADS-A return			
		**Yes** (N = 503)	**No** (N = 204)	**Difference** (95% CI)	**p-value**[1]
**Age** (years)	Mean (95% CI)	48.7 (47.8 to 49.6)	44.6 (43.1 to 46.0)	4.1 (2.5 to 5.8)	<0.001
**BMI** (kg/m2)	Mean (95% CI)	46.3 (45.7 to 46.8)	46.8 (45.8 to 47.7)	-0.5 (-1.6 to 0.6)	0.379
**Time from randomisation to surgery** (days)	Mean (95% CI)	92.6 (88.7 to 96.5)	91.5 (85.5 to 97.6)	1.1 (-6.1 to 8.3)	0.767
		**12 months post-randomisation HADS-D return**			
		**Yes** (N = 498)	**No** (N = 206)	**Difference** (95% CI)	**p-value**[1]
**Age** (years)	Mean (95% CI)	48.8 (47.9 to 49.7)	44.5 (43.1 to 45.9)	4.3 (2.7 to 6.0)	<0.001
**BMI** (kg/m2)	Mean (95% CI)	46.3 (45.7 to 46.9)	46.8 (45.8 to 47.7)	-0.5 (-1.6 to 0.6)	0.370
**Time from randomisation to surgery** (days)	Mean (95% CI)	92.9 (89.0 to 96.8)	91.8 (85.8 to 97.8)	1.1 (-6.1 to 8.3)	0.767

[1] p-value obtained from paired sample t-test statistic for mean difference by HADS return status

. *BMJ Open* 2024; 14:e071231. doi: 10.1136/bmjopen-2022-071231

Supplementary Results

Anxiety and depression following bariatric surgery

Table 3
Participant characteristics by repeat HADS-A questionnaire return status (categorical variables)

		12 months post-randomisation HADS-A return			
		**Yes** (%) N = 503 (71.15)	**No** (%) N = 204 (28.85)	**Odds Ratio for non-return**[2] (95% CI)	**p-value**[3]
**Sex**	Male (*n* 178)	126 (70.79)	52 (29.21)	1.00	0.903
	Female (*n* 529)	377 (71.27)	152 (28.73)	0.98 (0.67 to 1.42)	
**Ethnicity**	White (*n* 658)	472 (71.73)	186 (28.27)	1.00	0.217
	Other ethnic group[4] (*n* 49)	31 (63.27)	18 (36.73)	1.47 (0.80 to 2.70)	
**Marital status**	Married or civil partnership (*n* 395)	280 (70.89)	115 (29.11)	1.00	0.926
	Co-habiting (*n* 84)	62 (73.81)	22 (26.19)	0.86 (0.51 to 1.47)	
	Single (*n* 132)	92 (69.70)	40 (30.30)	1.06 (0.69 to 1.63)	
	Divorced, Separated, or Widowed[5] (*n* 96)	69 (71.88)	27 (28.12)	0.95 (0.58 to 1.56)	
**Smoking status**	Never smoked (*n* 317)	226 (71.29)	91 (28.71)	1.00	0.339
	Ex-smoker (*n* 343)	248 (72.30)	95 (27.70)	0.95 (0.68 to 1.33)	
	Current smoker (*n* 47)	29 (61.70)	18 (38.30)	1.54 (0.82 to 2.91)	
**Employment status**	Employed (*n* 455)	326 (71.65)	129 (28.35)	1.00	0.002
	Not in employment or student[6] (*n* 190)	123 (64.74)	67 (35.26)	1.38 (0.96 to 1.97)	
	Retired (*n* 62)	54 (87.10)	8 (12.90)	0.37 (0.17 to 0.81)	
**Income band**	≤10,000 (*n* 84)	59 (70.24)	25 (29.76)	1.00	0.247
	10,001 to 30,000 (*n* 289)	212 (73.36)	77 (26.64)	0.86 (0.50 to 1.46)	
	30,001 to 50,000 (*n* 150)	105 (70.00)	45 (30.00)	1.01 (0.56 to 1.81)	
	≥50,001 (*n* 91)	69 (75.82)	22 (24.18)	0.75 (0.38 to 1.47)	
	Not disclosed (*n* 92)	57 (61.96)	35 (38.04)	1.45 (0.77 to 2.72)	

[2] Odds ratio for questionnaire non-return calculated using logistic regression
[3] p-value obtained from likelihood ratio chi-square test
For calculation of Odds ratios associated with questionnaire non-return, categories with sub-groups containing ≤5% (*n* 35/707) total respondents were merged with the next largest sub-group to avoid data sparsity: [4] Includes participants who identified as African or Caribbean (*n* 28/707), Mixed ethnic group (*n* 13/707), Asian (*n* 3/707), or Other (*n* 5/707). [5] Participants who identified as separated (*n* 21/703) and widowed (*n* 11/703) combined with those who identified as divorced (*n* 64/703) as next largest category. [6] Participants who identified as students (*n* 6/703) combined with those not in employment (*n* 183/703)

. BMJ Open 2024; 14:e071231. doi: 10.1136/bmjopen-2022-071231

Supplementary Results

Anxiety and depression following bariatric surgery

Table 4
Baseline characteristics by repeat HADS-D questionnaire return status (categorical variables)

		12 months post-randomisation HADS-D return			
		**Yes** (%) N = 498 (70.74)	**No** (%) N = 206 (29.26)	**Odds Ratio for non-return**[2] (95% CI)	**p-value**[3]
**Sex**	Male (*n* 178)	126 (70.79)	52 (29.21)	1.00	0.987
	Female (*n* 526)	372 (70.72)	154 (29.28)	0.98 (0.69 to 1.46)	
**Ethnicity**	White (*n* 655)	468 (71.45)	187 (28.55)	1.00	0.139
	Other ethnic group[4] (*n* 49)	30 (61.22)	19 (38.78)	1.59 (0.87 to 2.89)	
**Marital status**	Married or civil partnership (*n* 390)	273 (70.00)	117 (30.00)	1.00	0.592
	Co-habiting (*n* 84)	64 (76.19)	20 (23.81)	0.73 (0.42 to 1.26)	
	Single (*n* 132)	90 (68.18)	42 (31.82)	1.09 (0.71 to 1.67)	
	Divorced, Separated, or Widowed[5] (*n* 98)	71 (72.45)	27 (27.55)	0.89 (0.54 to 1.45)	
**Smoking status**	Never smoked (*n* 317)	225 (70.98)	92 (29.02)	1.00	0.566
	Ex-smoker (*n* 340)	243 (71.47)	97 (28.53)	0.98 (0.70 to 1.37)	
	Current smoker (*n* 47)	30 (63.83)	17 (36.17)	1.39 (0.73 to 2.63)	
**Employment status**	Employed (*n* 451)	322 (71.40)	129 (28.60)	1.00	0.003
	Not in employment or student[6] (*n* 190)	122 (64.21)	68 (35.79)	1.39 (0.97 to 1.99)	
	Retired (*n* 63)	54 (85.71)	9 (14.29)	0.40 (0.33 to 0.49)	
**Income band**	≤10,000 (*n* 86)	59 (68.60)	27 (31.40)	1.00	0.108
	10,001 to 30,000 (*n* 286)	207 (72.38)	79 (27.62)	0.83 (0.49 to 1.41)	
	30,001 to 50,000 (*n* 149)	103 (69.13)	46 (30.87)	0.98 (0.55 to 1.73)	
	≥50,001 (*n* 91)	72 (79.12)	19 (20.88)	0.58 (0.29 to 1.14)	
	Not disclosed (*n* 91)	56 (61.54)	35 (38.46)	1.37 (0.73 to 2.54)	

2 Odds ratio for questionnaire non-return obtained using logistic regression
3 P-value obtained from likelihood ratio chi-square test
For calculation of Odds ratios associated with questionnaire non-return, categories with sub-groups containing ≤5% (*n* 35/707) total respondents were merged into the next largest sub-group to avoid data sparsity: **4** Includes participants who identified as African or Caribbean (*n* 28/704), Mixed ethnic group (*n* 13/707), Asian (*n* 3/707), or Other (*n* 5/707). **5** Participants who identified as separated (*n* 21/703) and widowed (*n* 11/703) combined with those who identified as divorced (*n* 66/703) as next largest category. **6** Participants who identified as students (*n* 7/703) combined with those not in employment (*n* 183/703).

. BMJ Open 2024; 14:e071231. doi: 10.1136/bmjopen-2022-071231
