# Supplementary material for: Prevalence and short-term change in symptoms of anxiety and depression following bariatric surgery: a prospective cohort study
Source: BMJ Open. 2024 Jan 3;14(1):e071231. doi: 10.1136/bmjopen-2022-071231 (PMC10773381; doi:10.1136/bmjopen-2022-071231)
Supplement: Author's manuscript [file bmjopen-2022-071231.draft_revisions.pdf]

BMJ Open is committed to open peer review. As part of this commitment we make the peer review history of every article we publish publicly available.

When an article is published we post the peer reviewers' comments and the authors' responses online. We also post the versions of the paper that were used during peer review. These are the versions that the peer review comments apply to.

The versions of the paper that follow are the versions that were submitted during the peer review process. They are not the versions of record or the final published versions. They should not be cited or distributed as the published version of this manuscript.

BMJ Open is an open access journal and the full, final, typeset and author-corrected version of record of the manuscript is available on our site with no access controls, subscription charges or pay-per-view fees (http://bmjopen.bmj.com).

If you have any questions on BMJ Open's open peer review process please email info.bmjopen@bmj.com

Prevalence and short-term change in symptoms of anxiety and depression following bariatric surgery: a prospective cohort study

Journal:	*BMJ Open*
Manuscript ID	bmjopen-2022-071231
Article Type:	Original research
Date Submitted by the Author:	24-Dec-2022

Complete List of Authors:	Gibb, Jonathan; University of Bristol, Centre for Academic Mental Health, Population Health Sciences, Bristol Medical School<br>Moran, Paul; University of Bristol, Centre for Academic Mental Health, Population Health Sciences, Bristol Medical School<br>Band-Sleeve Trial Management Group, (Group Authorship); University of Bristol, Bristol Trials Centre, Bristol Medical School, University of Bristol

Keywords:	Depression & mood disorders < PSYCHIATRY, Anxiety disorders < PSYCHIATRY, Adult surgery < SURGERY, EPIDEMIOLOGIC STUDIES

SCHOLARONE™ Manuscripts

I, the Submitting Author has the right to grant and does grant on behalf of all authors of the Work (as defined in the below author licence), an exclusive licence and/or a non-exclusive licence for contributions from authors who are: i) UK Crown employees; ii) where BMJ has agreed a CC-BY licence shall apply, and/or iii) in accordance with the terms applicable for US Federal Government officers or employees acting as part of their official duties; on a worldwide, perpetual, irrevocable, royalty-free basis to BMJ Publishing Group Ltd ("BMJ") its licensees and where the relevant Journal is co-owned by BMJ to the co-owners of the Journal, to publish the Work in this journal and any other BMJ products and to exploit all rights, as set out in our licence.

The Submitting Author accepts and understands that any supply made under these terms is made by BMJ to the Submitting Author unless you are acting as an employee on behalf of your employer or a postgraduate student of an affiliated institution which is paying any applicable article publishing charge ("APC") for Open Access articles. Where the Submitting Author wishes to make the Work available on an Open Access basis (and intends to pay the relevant APC), the terms of reuse of such Open Access shall be governed by a Creative Commons licence – details of these licences and which Creative Commons licence will apply to this Work are set out in our licence referred to above.

Other than as permitted in any relevant BMJ Author's Self Archiving Policies, I confirm this Work has not been accepted for publication elsewhere, is not being considered for publication elsewhere and does not duplicate material already published. I confirm all authors consent to publication of this Work and authorise the granting of this licence.

For peer review only

Prevalence and short-term change in symptoms of anxiety and depression following bariatric surgery: a prospective cohort study

Jonathan Gibb, Paul Moran, and the By-Band-Sleeve Trial Management Group

Corresponding author

Dr Jonathan Gibb

Centre for Academic Mental Health, Population Health Sciences

Bristol Medical School, University of Bristol

Oakfield Grove, Clifton, Bristol BS8 2BN

Jonathan.gibb@bristol.ac.uk

+44 (0)117 428 2489

Authors

Jonathan Gibb, Centre for Academic Mental Health, Population Health Sciences, Bristol Medical School, University of Bristol, Bristol, UK

Prof Paul Moran, Centre for Academic Mental Health, Population Health Sciences, Bristol Medical School, University of Bristol, Bristol, UK

By-Band-Sleeve Trial Management Group (Continued on next page)

Rob C Andrews PhD, Medical Research, University of Exeter Medical School, Exeter, UK

For peer review only - http://bmjopen.bmj.com/site/about/guidelines.xhtml

John Bessent, Patient representative, By-Band Sleeve Trial management Group, UK

Jane M Blazeby MD, NIHR Bristol Biomedical Research Centre Bristol Medical School, Population Health Sciences, University of Bristol, Bristol, UK

James P Byrne MD. University Hospital Southampton NHS Foundation Trust, Southampton, UK

Nicholas Carter MSc, Portsmouth Hospitals University NHS Trust, Portsmouth, UK

Caroline Clay (Deceased), Patient representative, By-Band-Sleeve Trial Management Group, UK

Jenny L Donovan PhD, Bristol Medical School, Population Health Sciences, University of Bristol, Bristol, UK

Eleanor A Gidman PhD, Bristol Trials Centre, Bristol Medical School, University of Bristol, Bristol, UK

Graziella Mazza PhD, Bristol Trials Centre, Bristol Medical School, University of Bristol, Bristol, UK

Mary O'Kane MSc, Dietetic Department, Leeds Teaching Hospitals NHS Trust, Leeds, UK

For peer review only - http://bmjopen.bmj.com/site/about/guidelines.xhtml

For peer review only

Barnaby C Reeves PhD, Bristol Trials Centre, Bristol Medical School, University of Bristol, Bristol, UK

Chris A Rogers PhD, Bristol Trials Centre, Bristol Medical School, University of Bristol, Bristol, UK

Nicki Salter DipHE, Somerset NHS Foundation Trust, Somerset, UK

Janice L Thompson PhD, School of Sport, Exercise & Rehabilitation Sciences, University of Birmingham, Birmingham, UK

Richard Welbourn MD, Somerset NHS Foundation Trust, Somerset, UK

Sarah Wordsworth PhD, Health Economics Research Centre, Nuffield Department of Population Health, University of Oxford, UK

Word Count (Excluding title page, abstract, references, figures and table): **2,944**

For peer review only - http://bmjopen.bmj.com/site/about/guidelines.xhtml

ABSTRACT

Objectives: Bariatric surgery is an effective treatment for severe obesity that leads to significant physical health improvements. Few studies have prospectively described the short-term impact of surgery on mental health using standardised case-finding measures for anxiety or depressive disorders. This study describes the prevalence and short-term course of these conditions following surgery.

Design: Prospective observational cohort study.

Setting: 12 National Health Service centres in England.

Participants: Participants studied took part in the By-Band-Sleeve study, a multi-centre randomised controlled trial evaluating the surgical management of severe obesity. We included participants who had undergone surgery (Gastric Bypass, Gastric Band or Sleeve Gastrectomy) within 6 months of randomisation.

Primary and secondary outcome measures: Anxiety and depression were assessed using the Hospital Anxiety and Depression Scale (HADS) at baseline and 12 months post-randomisation. Sociodemographic variables collected at pre-randomisation included Body Mass Index, Age, Sex, Ethnicity, Marital Status, Tobacco use, Employment Status, and Income Band.

Results: In our sample of 758 participants, 94.5% (n 716) and 93.9% (n 712) had completed baseline anxiety (HADS-A) and depression (HADS-D) subscales. At pre-randomisation 46.1% (n 330/716, 95% CI 42.4 to 49.7%) met clinical case criteria for

For peer review only - http://bmjopen.bmj.com/site/about/guidelines.xhtml

anxiety and 48.2% (n 343/712, 95% CI 44.5 to 51.8%) for depression. Among participants returning completed 12 months post-randomisation questionnaires (HADS-A n 503/716, HADS-D n 498/712), there was a highly significant reduction in the proportion of clinical cases with anxiety (-9.5%, 95% CI -14.3 to -4.8% $p < 0.001$) and depression (-22.3%, 95% CI -27.0 to -17.6% $p < 0.001$).

Conclusions: Almost half of people undergoing bariatric surgery had underlying anxiety or depressive symptoms. In the short term, these symptoms appear to substantially improve. Future work must identify whether these effects are sustained beyond the first post-randomisation year.

Trial registration: The By-Band-Sleeve Study is registered with ClinicalTrials.gov database (NCT02841527) and ISRCTN registry (ISRCTN00786323).

For peer review only

For peer review only - http://bmjopen.bmj.com/site/about/guidelines.xhtml

Strengths and limitations of this study

- A validated self-report measure, the Hospital Anxiety and Depression Scale (HADS), was used to detect anxiety and depressive disorders.

- Participants were recruited from the largest randomised controlled trial, to date, in bariatric surgery (The By-Band-Sleeve Study) from multiple NHS surgical centres in England.

- Although participants were re-assessed using the HADS at one-year post-randomisation, the total follow-up period from surgery was relatively short. It is possible that these changes were not maintained after the first post-operative year.

- With respect to surgical procedure, the participants were analysed as a whole group, rather than being stratified by surgery type (Gastric Bypass, Gastric Band or Sleeve Gastrectomy).

For peer review only

For peer review only - http://bmjopen.bmj.com/site/about/guidelines.xhtml

INTRODUCTION

Obesity and common mental disorders, such as anxiety and depression, contribute greatly to global disease burden and pose significant public health challenges [1, 2, 3]. There has been a recent focus on understanding the relationship between obesity and common mental disorders. Systematic reviews and meta-analyses of longitudinal studies have found a bi-directional relationship between being obese and developing a depressive disorder [4, 5] across both sexes, however a recently updated review found an elevated risk only among females [6]. Whilst there have been fewer longitudinal studies assessing the relationship between obesity and anxiety disorders, there is evidence of a positive association between the two conditions [7, 8]. These findings have coincided with a growing body of research studying the potential shared neurobiological (the role of prolonged inflammatory changes, cortisol dysregulation, metabolic dysfunction, and disrupted cellular signalling) pathways between obesity, anxiety states, and depression which may eventually give rise to a better understanding of these common co-morbidities [9, 10, 11].

When individuals with severe or complex obesity (Body Mass Index $\geq$40kg/m^2 or $\geq$35kg/m^2 with a significant co-morbidity) are unable to lose weight, and have attempted all relevant non-surgical measures, current guidelines in the United Kingdom recommend that bariatric surgery should be offered [12, 13]. Compared to non-surgical management, bariatric surgery has been shown to be an effective treatment for severe obesity and is associated with gains in overall life expectancy alongside increased remission rates of rates of obesity-related co-morbidities, such as type 2 diabetes mellitus [14, 15, 16].

Anxiety and depression following bariatric surgery

For peer review only - http://bmjopen.bmj.com/site/about/guidelines.xhtml

Previous research suggests that people who undergo bariatric surgery have higher rates of pre-operative depression compared to people with obesity who do not undergo surgery [17]. A 2016 meta-analysis of the international literature estimated that up to 23% of patients have a mood disorder at the time of surgery [18], with the pooled estimate for depression being 19% [95% CI 14 to 25%, 34 studies, *N* 12,009/51,908 participants] and anxiety 12% [95% CI 6 to 20%, 22 studies, *N* 10,515/38,459 participants]. In the short-term following surgery, there appears to be a reduction in the prevalence and severity of depression [19] however there remains uncertainty around the course of anxiety symptoms [19, 20, 21]. Previous literature on the mental health status of bariatric surgical recipients has often been limited due to the use of uncertain diagnostic criteria, measures for common mental disorders which do not address anxiety symptoms separately from depressive symptoms, and a lack of reporting on symptom severity [20]. As rates of severe and complex obesity increase, there is a clear need to better understand the prevalence and course of common mental health problems following surgery. This is particularly timely as recent research has found an increased risk of self-harm among those who undergo weight loss surgery [22, 23] compared to people with obesity who do not.

This paper presents findings from an analysis of data from the largest randomised controlled trial to date of bariatric surgery – the By-Band-Sleeve study [24, 25]. The study is comparing the clinical and cost effectiveness of gastric banding (Band), laparoscopic gastric bypass (Bypass) or sleeve gastrectomy (Sleeve) which are surgical treatments for severe obesity. The objectives of this sub-study were to describe the prevalence, and severity, of anxiety and depressive symptoms among

For peer review only - http://bmjopen.bmj.com/site/about/guidelines.xhtml

participants who underwent surgery within 6 months of randomisation at baseline (pre-randomisation) and following surgery (of any type) at 12 months post-randomisation.

For peer review only

For peer review only - http://bmjopen.bmj.com/site/about/guidelines.xhtml

METHOD

Participants

Participants were included in this sub-study if they had taken part in the By-Band-Sleeve study, had undergone surgery (irrespective of procedure type) within six months of randomisation, and had completed the Hospital Anxiety and Depression Scale after informed consent and before randomisation. By-Band-Sleeve study exclusion criteria included: previous gastric surgery for severe and complex obesity, previous abdominal surgery or gastro-intestinal conditions that precludes the surgical intervention, large abdominal ventral hernia or hiatus hernia >5cm, pregnancy, clinical conditions (such as Crohn's disease, liver cirrhosis and portal hypertension), known silicone allergy, or active participation in another interventional research study which may interfere with the By-Band-Sleeve study.

To understand the effect of surgery on mental health, participants were excluded if they had not undergone surgery within six months of randomisation. This cut-off of six months from enrolment was selected a priori in the event of participants waiting a prolonged time for surgery to take place (for example, due to the ongoing impact of the COVID-19 pandemic on elective surgery), which may have reduced the accuracy and relevance of baseline assessment of pre-operative mental health status. In total, 1,351 participants were randomised to the By-Band-Sleeve study and in this paper, we report on the mental health outcomes of the 758 eligible participants.

Primary measure

The Hospital Anxiety and Depression Scale (HADS) was completed at pre-randomisation (study enrolment or 'baseline') and at 12 months post-randomisation.

For peer review only - http://bmjopen.bmj.com/site/about/guidelines.xhtml

HADS is a 14-item questionnaire (7 questions for anxiety 'A' and 7 questions for depressive 'D' symptoms), which asks the participant to score each item between 0 to 3 based on their level of agreement. A sub-scale total score of less than 8 is considered normal, 8 to 10 suggestive of possible anxiety or depressive disorder, and a score greater than 11 is suggestive of a probable disorder [26]. Previous research has determined that a sub-scale score of $\geq$8 represents the optimal case cut-off for clinical anxiety and depressive disorders, in terms of the balance between sensitivity and specificity [27].

Secondary measures

Baseline characteristics and demographic data for participants were collected on study enrolment. These included Body Mass Index (BMI), Age, Sex, Ethnicity, Marital Status, Tobacco use, Employment Status, and Income Band. Time from randomisation to surgery and number of centres participating were described.

Statistical analysis

Analyses were undertaken using Stata Version 16. Returned HADS questionnaires were assessed for completion of the 7-item anxiety (HADS-A) and depression (HADS-D) subscales. Participants who fully completed either subscale had a total symptom score calculated. The proportions of participants who met case criteria for possible anxiety and depression (defined as HADS-A/D $\geq$8) were described alongside baseline sociodemographic variables. The median symptom score (and interquartile range) was calculated for participants who had completed a subscale at both baseline and 12 months post-randomisation. The Wilcoxon signed-rank test was used to assess the statistical significance of any change in median symptom

For peer review only - http://bmjopen.bmj.com/site/about/guidelines.xhtml

score. The change in proportions of participants with possible depression or anxiety at pre-randomisation compared to 12 months post-randomisation was calculated alongside 95% confidence intervals. McNemar's chi-squared test was used quantify the strength of association.

Missing data and loss to follow-up

A complete case analysis was undertaken in which participants with fully completed HADS-A or HADS-D questionnaire subscales were included in the analysis. The characteristics of participants who did not return completed questionnaires at 12 months post-randomisation was compared to returners with respect to baseline symptom scores, proportion of clinical cases, and sociodemographic variables. For categorical variables, cross-tabulation was used to compare the distribution of baseline characteristics by repeat subscale return status. Odds ratios (with 95% confidence intervals) for questionnaire return status were calculated using logistic regression for each categorical variable. For continuous variables, which were normally distributed, a two-sample t-test was used to compare whether the mean value (such as BMI, age, and time from randomisation to surgery) differed by return status.

Ethical approval

The By-Band-Sleeve study gained National Health Service (NHS) ethics approval from the Southwest Frenchay Research Ethics Committee (REC No: 11/SW/0248) in 2011. The study is sponsored by the University of Bristol and was granted Health Research Authority (HRA) Approval in 2017. The By-Band-Sleeve Study is

For peer review only - http://bmjopen.bmj.com/site/about/guidelines.xhtml

registered with the National Institutes of Health ClinicalTrials.gov database (NCT02841527) and ISRCTN registry (ISRCTN00786323).

Patient and public involvement

This sub-study features data obtained from participants who took part in the By-Band-Sleeve study. Patients and public were involved in By-Band-Sleeve Study throughout the design and conduct of the trial. Patient representatives on the Trial Management Group contributed towards the writing of this manuscript and are recognised as co-authors. The results of this sub-study will be disseminated through the By-Band-Sleeve Patient and Public Involvement Group and summarised, for a non-specialist audience, on the study (www.bybandsleevestudy.blogs.bristol.ac.uk) webpage following publication.

For peer review only - http://bmjopen.bmj.com/site/about/guidelines.xhtml

RESULTS

Seven hundred and fifty-eight By-Band-Sleeve study participants who had undergone surgery at the time of undertaking this work and who were within six months of randomisation were included [***Figure 1***]. Participants were recruited between January 2013 and September 2019 from 12 NHS surgical centres in England. Demographic characteristics by baseline (pre-randomisation) total HADS scores (normal, possible, probable disorder) are displayed in [***Table 1***]. At the point of randomisation, the mean age of participants was 47.8 (Standard Deviation, *SD* 10.6) years and the mean BMI was 46.3 (SD 6.7) kg/m^2. In total 570/758 (75.2%) participants were female.

Participant characteristics by baseline HADS scores

Of the 758 participants, 737 (97.2%) had returned baseline HADS questionnaires. For the subscales, baseline completion for the HADS-A was 94.5% (716/758) and 93.9% (712/758) for the HADS-D. The median symptom score for both baseline HADS-A and HADS-D was 7 (IQR 4 – 10). The proportion of individuals meeting case criteria for a possible, or probable, anxiety disorder was 46.1% (*n* 330/716, 95% CI 42.4 to 49.7%) and 48.2% (*n* 343/712, 95% CI 44.5 to 51.8%) for depression. Time from randomisation to surgery varied with a mean time of 92.1 (SD 44.4) days and was similar across the groups when stratified by baseline anxiety and depression status.

TABLE 1: Demographic data by baseline HADS-A and HADS-D scores. Case categories based on the original HADS cut-offs proposed by Zigmond & Snaith, 1983.

For peer review only - http://bmjopen.bmj.com/site/about/guidelines.xhtml

Table 1: Demographic data by baseline HADS scores

		**HADS-A** (n = 716) Anxiety case category			**HADS-D** (n = 712) Depression case category		
		**Nil**	**Possible**	**Probable**	**Nil**	**Possible**	**Probable**
		<8	8 - 10	≥11	<8	8 - 10	≥11
**Total**	n (%)	386 (53.9)	159 (22.2)	171 (23.9)	369 (51.8)	181 (25.4)	162 (22.8)
**Age** (years)	mean (SD)	48.12 (10.5)	46.81 (10.7)	46.26 (10.6)	47.76 (10.5)	47.67 (11.2)	46.44 (10.2)
**BMI** (kg/m²)	mean (SD)	45.96 (6.4)	47.00 (7.1)	46.88 (7.0)	46.24 (6.6)	46.47 (6.9)	46.81 (7.0)
**Sex** n (%)	Male	102 (56.4)	42 (23.2)	37 (20.4)	90 (49.7)	50 (27.6)	41 (22.7)
	Female	284 (53.1)	117 (21.9)	134 (25.1)	279 (52.5)	131 (24.7)	121 (22.8)
**Ethnicity** n (%)	White	359 (53.9)	150 (22.5)	157 (23.6)	340 (51.4)	173 (26.1)	149 (22.5)
	African or Caribbean	17 (60.7)	6 (21.4)	5 (17.9)	17 (60.7)	4 (14.3)	7 (25.0)
	Mixed	7 (50.0)	2 (14.3)	5 (35.7)	8 (57.1)	1 (7.1)	5 (35.7)
	Asian	0 (0.0)	0 (0.0)	3 (100.0)	2 (66.7)	0 (0.0)	1 (33.3)
	Other	3 (60.0)	1 (20.0)	1 (20.0)	2 (40.0)	3 (60.0)	0 (0.0)
**Marital status** n (%)	Married or civil partnership	223 (55.8)	83 (20.8)	94 (23.5)	205 (52.0)	95 (24.1)	94 (23.9)
	Co-habiting	50 (58.8)	19 (22.4)	16 (18.8)	45 (52.9)	22 (25.9)	18 (21.2)
	Single	69 (51.5)	32 (23.9)	33 (24.6)	70 (52.2)	34 (25.4)	30 (22.4)
	Divorced	28 (43.1)	19 (29.2)	18 (27.7)	35 (52.2)	19 (28.4)	13 (19.4)
	Separated	11 (52.4)	5 (23.8)	5 (23.8)	11 (52.4)	7 (33.3)	3 (14.3)
	Widowed	5 (45.5)	1 (9.1)	5 (45.5)	3 (27.3)	4 (36.4)	4 (36.4)
**Smoking status** n (%)	Never smoked	181 (56.4)	61 (19.0)	79 (24.6)	156 (48.8)	83 (25.9)	81 (25.3)
	Ex-smoker	181 (52.0)	90 (25.9)	77 (22.1)	189 (54.8)	83 (24.1)	73 (21.2)
	Current smoker	24 (51.1)	8 (17.0)	15 (31.9)	24 (51.1)	15 (31.9)	8 (17.0)
**Employment status** n (%)	Employed	285 (62.1)	93 (20.3)	81 (17.7)	267 (58.8)	110 (24.2)	77 (17.0)
	Not in employment	62 (33.0)	50 (26.6)	76 (40.4)	70 (37.2)	51 (27.1)	67 (35.6)
	Student	4 (57.1)	2 (28.6)	1 (14.3)	4 (57.1)	2 (28.6)	1 (14.3)
	Retired	35 (56.5)	14 (22.6)	13 (21.0)	28 (44.4)	18 (28.6)	17 (27.0)
**Income band (GBP)** n (%)	≤ £10,000	28 (31.5)	29 (32.6)	32 (36.0)	35 (35.6)	30 (33.3)	28 (31.1)
	10,001-30,000	159 (52.5)	60 (20.6)	73 (25.0)	156 (54.0)	69 (23.9)	64 (22.2)
	30,001-50,000	89 (59.3)	36 (24.0)	25 (16.7)	80 (53.7)	41 (27.5)	28 (18.8)
	50,001-70,000	44 (68.8)	12 (18.8)	8 (12.5)	38 (60.3)	15 (23.8)	10 (15.9)
	> £70,001	18 (66.7)	6 (17.2)	3 (11.1)	20 (71.4)	6 (21.7)	2 (7.1)

For peer review only - http://bmjopen.bmj.com/site/about/guidelines.xhtml

	Not disclosed	47 (50.5)	16 (17.2)	30 (32.3)	42 (45.7)	20 (21.7)	30 (32.6)
	Missing	1 (100.0)	0 (0.0)	0 (0.0)	1 (100.0)	0 (0.0)	0 (0.0)

Prevalence of anxiety and depression at 12 months post-randomisation

At 12 months post-randomisation, nine of the participants who had completed baseline HADS-A and eight of the participants who had completed baseline HADS-D had withdrawn or died. [***Figure 1***]. After accounting for these individuals, the proportion of questionnaires returned complete was 71.1% (*n* 503/707) for the HADS-A and 70.7% (*n* 498/704) for the HADS-D. The median HADS score decreased from 7 at baseline to 5 (IQR 2 – 10) for anxiety and to 3 (IQR 1 – 7) for depression [***Table 2***] at 12 months post-randomisation among participants who completed questionnaires at both timepoints. There was a highly statistically significant ($p < 0.001$) decrease in both HADS-A and HADS-D scores [***Figure 2***]. This was coupled with a highly significant reduction in the proportion of participants meeting caseness for anxiety (9.5% decrease, 95% CI -14.3 to -4.8%, $p < 0.001$) and depression (22.3% decrease, 95% CI -27.0 to -17.6%, $p < 0.001$) at 12 months post-randomisation [***Figure 3***] when compared to baseline.

Whilst the overall proportion of cases of anxiety and depression decreased, the mental health of a small number of participants appeared to decline over the course of the 12-month follow-up, with 4.4% (*n* 22/498) of participants developing possible depression and 9.2% (*n* 46/503) developing a possible anxiety disorder (***Table 3***).

For peer review only - http://bmjopen.bmj.com/site/about/guidelines.xhtml

Table 2: Change in HADS scores from baseline to 12 months post-randomisation						
				Proportions		
		**Median score** (IQR)	**Cases** (%)	**Change** (%)	**95% CI** (%)	**P-value**
**HADS-A** Anxiety (*n* 503)	Baseline	7 (4 – 10)	45.3	-9.5	-14.3 to -4.8	<0.001
	12 months post-randomisation	5 (2 – 10)	35.8			
**HADS-D** Depression (*n* 498)	Baseline	7 (4 – 10)	46.4	-22.3	-27.0 to -17.6	<0.001
	12 months post-randomisation	3 (1 – 7)	24.1			

TABLE 2: Frequency and percentage change in clinical cases for individuals who underwent surgery within 6 months of randomisation with completed questionnaires at both timepoints. p-value associated with change in case proportions obtained from McNemar's chi-squared test statistic.

Table 3: Change in clinical cases from baseline to 12 months post-randomisation		
		**Frequency** (%)
**Anxiety** HADS-A (*n* 503)	Case unchanged	134 (26.6)
	Case to non-case	94 (18.7)
	Non-case unchanged	229 (45.5)
	Non-case to case	46 (9.2)
**Depression** HADS-D (*n* 498)	Case unchanged	98 (19.7)
	Case to non-case	133 (26.7)
	Non-case unchanged	245 (49.2)
	Non-case to case	22 (4.4)

TABLE 3: Proportions of individuals with respect to change in case category (subscale score of ≥8) between baseline and 12 months post-randomisation.

FIGURE 2

FIGURE 3

For peer review only - http://bmjopen.bmj.com/site/about/guidelines.xhtml

Characteristics of 12-month post-randomisation HADS questionnaire returners and non-returners

The prevalence of baseline anxiety was similar (HADS-A Returners 54.7%, Non-returners 53.4% [*Difference* 1.3%, 95% CI -9.4 to +6.9%, p = 0.764]) amongst those who did and did not return a completed 12-month questionnaire. Those who returned questionnaires had a slightly higher prevalence of baseline depression than those who did not return questionnaires (HADS-D Returners 53.6%, Non-returners 47.1% [*Difference* 6.5%, 95% CI -1.6 to +14.6%, p = 0.115]). Baseline BMI, participant sex, ethnicity, marital status, smoking status, and self-reported income were not associated with repeat HADS questionnaire return. [***Appendix A***]

Factors associated with 12-month post-randomisation HADS return included participant age and employment status. Participants who returned completed anxiety or depression questionnaires were on average older (HADS-A: 4.1 years older, 95% CI 2.5 to 5.8, p < 0.001; HADS-D: 4.3 years older, 95% CI 2.7 to 6.0, p < 0.001) than participants who did not return completed questionnaires. Compared to individuals who were in employment, being retired at baseline was associated with an increased odds of completed HADS-A (Odds Ratio (OR) 2.7, 95% CI 1.2 to 5.8, p < 0.01) and HADS-D (OR 2.4, 95% CI 1.1 to 5.0, p < 0.05) questionnaire return at 12 months post-randomisation.

[Appendix A – Supplementary Results]

For peer review only - http://bmjopen.bmj.com/site/about/guidelines.xhtml

DISCUSSION

In this study of the course of common mental health disorders in a population of randomised participants undergoing bariatric surgery, nearly half of the sample met criteria for possible or probable anxiety or depression on trial enrolment. Following surgery, substantial reductions in the proportion of participants with possible depression and anxiety were observed at 12 months post-randomisation. The greatest reduction was observed in symptoms of depression, where there was over a 20% decrease in prevalence. Whilst most participants reported an improvement in their mental health, over a third retained symptoms of an underlying anxiety disorder and a quarter of participants met criteria for a depressive disorder at 12 months post-randomisation.

Compared to previously published research utilising the HADS, we found higher a prevalence of pre-operative anxiety and depression in our study sample. Karlsson et al. described HADS scores amongst a consecutive sample of participants (*n* 655) who took part in the Swedish Obese Subjects (SOS) study [28] and underwent bariatric surgery. Using identical cut-off points to those used in our study, the prevalence of pre-operative anxiety was 34% and that of depression was 24% among those who were surgically treated. Whilst the mean age of their sample was comparable to ours, the mean BMI (41.9 SD 4.2 kg/m^2) was lower. The increased BMI among our sample may reflect the higher rate of adult obesity within the UK population, alongside the substantially lower number of bariatric surgical procedures taking place in the UK compared to Sweden and other European countries [29]. This may also be linked to the higher levels of depression and anxiety in our sample. In a prospective study of people who underwent bariatric surgery (*n* 153) recruited from

For peer review only - http://bmjopen.bmj.com/site/about/guidelines.xhtml

six surgical centres in Austria and Germany, Burgmer et al. [30] found that 40.5% of the sample had depression (HADS-D $\geq$8) at baseline which decreased to 17.1% after one year following surgery. Participants had a higher mean BMI (51.3 SD 8.4 kg/m^2) compared to those enrolled in this study. However, they did not find any significant changes in anxiety caseness which could have arisen due to the use of a higher (HADS-A $\geq$10) case cut-off score.

For peer review only

Our study has several strengths. To our knowledge, it is the largest prospective study to assess the short-term effects of bariatric surgery on anxiety and depression in the UK. Participants were screened for anxiety and depression using a validated scale. Those who took part were recruited from 12 UK NHS surgical centres that are likely to be representative of the national population undergoing bariatric surgery, compared to those sampled from a single geographical site. We also report the effect size, with respect to change in prevalence of anxiety and depression – an important metric which has been missing from previously published studies in the field [18, 20]. There were also some important limitations. There was a significant questionnaire non-return rate of around 30% at 12 months post-randomisation. Whilst we did not find an association between having poorer mental health at baseline and questionnaire return, it is possible that individuals who did not return repeat HADS questionnaires may have later developed anxiety or depressive disorders following randomisation. We have also not explored the BMI of participants returning or not returning questionnaires in follow up because this primary outcome weight data remains confidential until analyses of the main trial is completed. It is possible that participants not returning questionnaires have regained weight and have poor mental health. In terms of sociodemographic characteristics, the

For peer review only - http://bmjopen.bmj.com/site/about/guidelines.xhtml

participants were predominantly female, identified as being from a White British ethnic background, and in employment at the time of study. It is therefore possible that the findings are not generalisable to the other groups undergoing bariatric surgery, particularly males and individuals from ethnic minorities. It is also plausible that responses to the pre-randomisation HADS questionnaires may have been influenced or affected by social desirability bias, particularly if participants incorrectly perceived that disclosure of their mental health difficulties was going to influence the likelihood of surgery. The role of mental health stigma and marginalisation has been highlighted throughout qualitative research [31, 32] featuring surgery recipients.

CONCLUSIONS

Our study highlights the very high prevalence of pre-operative psychological morbidity amongst people undergoing bariatric surgery for the treatment of severe or complex obesity. An improvement in symptoms of anxiety and depression was observed following surgery amongst participants who had returned completed questionnaires. Research has demonstrated a disparity in mental-health quality of life compared to physical-health quality of life gains following surgery [33]. Future work must be undertaken to establish whether such improvements in mental health are sustained over longer periods of time and to determine the mechanisms underpinning these associations.

For peer review only - http://bmjopen.bmj.com/site/about/guidelines.xhtml

Author contributions

Jonathan Gibb MB ChB: conception of study idea was based on available trial data (conceived and developed by Trial Management Group), undertook data-analysis, wrote first draft of final manuscript, contributed to final manuscript. Centre for Academic Mental Health, Population Health Sciences, Bristol Medical School, University of Bristol, UK

Paul Moran MD: supervised project, developed study idea, contributed to statistical analysis plan, contributed to draft and final manuscript. Centre for Academic Mental Health, Population Health Sciences, Bristol Medical School, University of Bristol, UK

By-Band-Sleeve Trial Management Group

Rob C Andrews PhD, On Trial Management Group: contributed to final manuscript and reviewed final manuscript. Medical Research, University of Exeter Medical School, UK

John Bessent, On Trial Management Group, patient representative: reviewed final manuscript.

Jane M Blazeby MD, By-Band-Sleeve Chief Investigator, and conception of study idea. On Trial Management Group: supervised project, developed study idea, contributed to statistical analysis plan, contributed to draft and final manuscript. NIHR Bristol Biomedical Research Centre Bristol Medical School, Population Health Sciences, University of Bristol, Bristol, UK

For peer review only - http://bmjopen.bmj.com/site/about/guidelines.xhtml

For peer review only

James P Byrne MD, On Trial Management Group: contributed to final manuscript and reviewed final manuscript. University Hospital Southampton NHS Foundation Trust, UK

Nicholas Carter MSc, On Trial Management Group: reviewed final manuscript. Portsmouth Hospitals University NHS Trust, UK

Caroline Clay (Deceased), On Trial Management Group, patient representative: reviewed final manuscript.

Jenny L Donovan PhD, On Trial Management Group: contributed to final manuscript and reviewed final manuscript. Population Health Sciences, Bristol Medical School, University of Bristol, UK

Eleanor A Gidman PhD, On Trial Management Group: prepared the sub-study dataset, contributed to statistical analysis plan, contributed to final manuscript, and reviewed final manuscript. Bristol Trials Centre, Bristol Medical School, University of Bristol, UK

Graziella Mazza PhD, By-Band-Sleeve Study Co-ordinator, On Trial Management Group: contributed to the study design, contributed to final manuscript, and reviewed final manuscript. Bristol Trials Centre, Bristol Medical School, University of Bristol, UK

For peer review only - http://bmjopen.bmj.com/site/about/guidelines.xhtml

For peer review only

Mary O'Kane MSc, On Trial Management Group: contributed to final manuscript and reviewed final manuscript. Dietetic Department, Leeds Teaching Hospitals NHS Trust, UK

Barnaby C Reeves PhD, On Trial Management Group: reviewed final manuscript. Bristol Trials Centre, Bristol Medical School, University of Bristol, UK

Chris A Rogers PhD, By-Band-Sleeve Lead methodologist, On Trial Management Group: contributed to statistical analysis plan, contributed to final manuscript, and reviewed final manuscript. Bristol Trials Centre, Bristol Medical School, University of Bristol, UK

Nicki Salter DipHE, On Trial Management group: reviewed final manuscript. Somerset NHS Foundation Trust, UK

Janice L Thompson PhD, On Trial Management group: contributed to final manuscript and reviewed final manuscript. School of Sport, Exercise & Rehabilitation Sciences, University of Birmingham, UK

Richard Welbourn MD, On Trial Management group: contributed to draft manuscript, contributed to final manuscript, and reviewed final manuscript. Somerset NHS Foundation Trust, UK

For peer review only

Sarah Wordsworth PhD, On Trial Management group: reviewed final manuscript.
Health Economics Research Centre, Nuffield Department of Population Health, University of Oxford, UK

Acknowledgements

Independent data monitoring committee

Craig Ramsay (Chair), Health Services Research Unit, University of Aberdeen UK

Nick Finer, UCLH centre for weight loss, metabolic and endocrine surgery, London, UK

Torsten Olbers, Sahlgrenska University Hospital, Sweden

Trial Steering Committee

Julia Brown (Chair), Leeds Institute of Clinical Trials Research, Leeds UK

John Dixon, Baker IDI Heart and Diabetes Institute, Melbourne, Australia

Steve Morris, Department of Public Health and Primary Care, University of Cambridge

Jodie Smith, Patient representative

Michel Suter, Université de Lausanne, Switzerland

John Wilding, Clinical Sciences Centre, University Hospital Aintree, Liverpool, UK

Lead research nurses

Sally Abbott, Benita Adams, Alison Fletcher, Hassina Furreed, Hussain Gordon, Jennifer Henderson, Helen Horton, Tracey Lee, Amy Long, Melody MacGregor, Sarah Matthias, Maria Moon, Catherine Moriarty, Rosemary Mullett, Nicki Salter, Jill Townley

For peer review only

Research nurses and practitioners

Philippa Allison, Fiona Brogan, Katie Cook, Paul Corrigan, , Anne Daw, Naomi Dindol, Jacqueline Dingle, Eve Fletcher, Jeremy Gilbert, Ana Gill, Beth Greenslade, Andrew Guy, Madeleine Hawkes, Emma Holzer, Lianne Hufton, Lucy Johnstone, Jasmine Jose, Susan Kelly, Krishna Kholia, Jasmina Mandair, Claire Mason, Priya Mathew, Maxine Nixon, Madeleine Pappas, Mark Priestley, Tracey Robson, Jana Rojkova, Rachel Schranz, Barbara Watkins, Louise White

Bariatric surgeons

Ahmed Ahmed, Sanjay Agrawal, Sara Ajaz, Waleed Al-Khyatt, Sherif Awad, Altaf Awan, Shlok Balupuri, Ashok Bohra, James Byrne, Richard Byrom, Nicholas Carter, Michael Clarke, Allwyn Cota, Markos Daskalakis, Nick Davies, Simon Dexter, Ian Finlay, Jeremy Hayden, James Hopkins, Noah Howes, Khaleel Fareed, Sherif Hakky, James Hewes, Neil Jennings, Jamie Kelly, Ben Knight, Yashwant Koak, Moorthy Krishna, Paul Leeder, John Loy, Brijesh Madhok, Kamal Mahawar, David Mahon, Matthew Mason, Samir Mehta, Rajwinder Nijjar, Hamish Noble, Alan Osborne, Dimitri Pournaras, Sanjay Purkayastha, Martin Richardson, Abeezar Sarela, Rishi Singhal, Peter Small, Shaw Somers, Paul Super, Christos Tsironis, Richard Welbourn

For peer review only

For peer review only - http://bmjopen.bmj.com/site/about/guidelines.xhtml

Declaration of interests

From authorship group:

James P Byrne MD, On Trial Management Group, is on the medical advisory board for the company Oxford Medical Products. All other authors declare no competing interests.

From those acknowledged:

Sanjay Agrawal received a royalty from Springer Publishers for being Editor of the book – 'Obesity, Bariatric and Metabolic Surgery-A Practical Guide' in addition to honoraria for lectures given at national and international bariatric meetings; Sanjay Agrawal is also the Director of Bariatric and Metabolic Surgery UK: Not for Profit – Charity Company; Company No: 11729612, Registered in England & Wales. Sherif Awad receives honoraria for lectures delivered at bariatric meetings. Nick Finer is the Chair of the Trial Steering Committee for the iPREVENT study (NIHR funded EME Project:15/185/16 - Increase colonic propionate as a method of preventing weight gain in young adults). John Dixon previously served as a consultant for the company Reshape who own the LapBand.

For peer review only - http://bmjopen.bmj.com/site/about/guidelines.xhtml

Ethical approval

The By-band study gained National Health Service (NHS) ethics approval from the South West Frenchay Research Ethics Committee (REC No: 11/SW/0248) on the 6th December 2011 and on the 8th May 2015 the Ethics Committee granted ethical approval to adapt the study from a two group (By-Band) to a three group (By-Band-Sleeve) trial. REC approval applies to all NHS sites taking part in the study. The study is sponsored by the University of Bristol and it is the responsibility of the sponsor to ensure that all the conditions of the study are complied with. In addition, By-Band-Sleeve study was processed under pre-Health Research Authority (HRA) Approval systems, the study was granted HRA approval on the 24th July 2017. The By-Band-Sleeve Study is registered with the National Institutes of Health ClinicalTrials.gov database (NCT02841527) and ISRCTN registry (ISRCTN00786323).

Funding statement

The By-Band-Sleeve study is funded by the United Kingdom National Institute for Health Research (NIHR) HTA programme (ref: 09/127/53). The views and opinions expressed are those of the authors and do not necessarily reflect those of the HTA programme, the NIHR, the UK NHS, or the Department of Health. The trial is being delivered in collaboration with Bristol Trials Centre, a UKCRC registered clinical trials unit which is in receipt of NIHR CTU support funding. This study was supported by the NIHR Biomedical Research Centre at University Hospitals Bristol and Weston NHS Foundation Trust and the University of Bristol. Jane M Blazeby MD is partially funded by the Bristol NIHR Biomedical research centre.

For peer review only - http://bmjopen.bmj.com/site/about/guidelines.xhtml

Data sharing statement

Data may be obtained from a third party and are not publicly available. Data will not be made available for sharing until after publication of the main results of the randomised trial. Thereafter, anonymised individual patient data will be made available for secondary research, conditional on assurance from the secondary researcher that the proposed use of the data is compliant with the MRC Policy on Data Preservation and Sharing regarding scientific quality, ethical requirements, and value for money. For more information on requirements, please refer to the By-Band-Sleeve Study Protocol [25].

REFERENCES

[1] Dai H, Alsalhe TA, Chalghaf N, Riccò M, Bragazzi NL, Wu J. The global burden of disease attributable to high body mass index in 195 countries and territories, 1990-2017: An analysis of the Global Burden of Disease Study. PLoS Med. 2020;17(7):e1003198.

[2] Vos T, Lim SS, Abbafati C, Abbas KM, Abbasi M, Abbasifard M, et al. Global burden of 369 diseases and injuries in 204 countries and territories, 1990–2019: a systematic analysis for the Global Burden of Disease Study 2019. The Lancet. 2020;396(10258):1204-22.

[3] Santomauro DF, Mantilla Herrera AM, Shadid J, Zheng P, Ashbaugh C, Pigott DM, et al. Global prevalence and burden of depressive and anxiety disorders in 204 countries and territories in 2020 due to the COVID-19 pandemic. The Lancet. 2021;398(10312):1700-12.

[4] Luppino FS, Wit LMd, Bouvy PF, Stijnen T, Cuijpers P, Penninx BWJH, et al. Overweight, Obesity, and Depression: A Systematic Review and Meta-analysis of Longitudinal Studies. Arch. Gen. Psychiatry. 2021;67:220-9.

For peer review only - http://bmjopen.bmj.com/site/about/guidelines.xhtml

[5] Pan A, Sun Q, Czernichow S, Kivimaki M, Okereke OI, Lucas M, et al. Bidirectional association between depression and obesity in middle-aged and older women. Int J Obes (Lond). 2012;36(4):595-602.

[6] Jung SJ, Woo H-t, Cho S, Park K, Jeong S, Lee YJ, et al. Association between body size, weight change and depression: systematic review and meta-analysis. British Journal of Psychiatry. 2017;211(1):14-21.

[7] Gariepy G, Nitka D, Schmitz N. The association between obesity and anxiety disorders in the population: a systematic review and meta-analysis. International journal of obesity. 2010 Mar;34(3):407-19.

[8] de Wit L, Have Mt, Cuijpers P, de Graaf R. Body Mass Index and risk for onset of mood and anxiety disorders in the general population: Results from the Netherlands Mental Health Survey and Incidence Study-2 (NEMESIS-2). BMC Psychiatry. 2022;22(1):522. 2.

[9] F ulton S, Décarie-Spain L, Fioramonti X, Guiard B, Nakajima S. The menace of obesity to depression and anxiety prevalence. Trends in Endocrinology & Metabolism. 2022;33(1):18-35.

[10] McLaughlin AP, Nikkheslat N, Hastings C, Nettis MA, Kose M, Worrell C, et al. The influence of comorbid depression and overweight status on peripheral inflammation and cortisol levels. Psychological Medicine. 2022;52(14):3289-96.

[11] Borgland SL. Can treatment of obesity reduce depression or vice versa? J Psychiatry Neurosci. 2021;46(2):E313-e8.

[12] National Institute for Health and Care Excellence. Obesity: identification, assessment and management [Internet]. [London]: NICE; 2014 [updated 2022 Sept 08; cited 2022 Dec 04]. (Clinical guideline [CG189]). Available from: https://www.nice.org.uk/guidance/cg189

[13] Douglas IJ, Bhaskaran K, Batterham RL, Smeeth L. Bariatric Surgery in the United Kingdom: A Cohort Study of Weight Loss and Clinical Outcomes in Routine Clinical Care. PLoS Med. 2015;12(12):e1001925.

For peer review only - http://bmjopen.bmj.com/site/about/guidelines.xhtml
For peer review only

[14] Syn NL, Cummings DE, Wang LZ, Lin DJ, Zhao JJ, Loh M, et al. Association of metabolic-bariatric surgery with long-term survival in adults with and without diabetes: a one-stage meta-analysis of matched cohort and prospective controlled studies with 174;772 participants. Lancet. 2021;397(10287):1830-41.

[15] Gloy VL, Briel M, Bhatt DL, Kashyap SR, Schauer PR, Mingrone G, et al. Bariatric surgery versus non-surgical treatment for obesity: a systematic review and meta-analysis of randomised controlled trials. BMJ. 2013;347:f5934.

[16] Nguyen NT, Varela JE. Bariatric surgery for obesity and metabolic disorders: state of the art. Nat. Rev. Gastroenterol. Hepatol. 2016;14:160-9.

[17] Booth H, Khan O, Prevost AT, Reddy M, Charlton J, Gulliford MC. Impact of bariatric surgery on clinical depression. Interrupted time series study with matched controls. J. Affect. Disord. 2015;174:644-9.

[18] Dawes AJ, Maggard-Gibbons M, Maher AR, Booth MJ, Miake-Lye I, Beroes JM, et al. Mental Health Conditions Among Patients Seeking and Undergoing Bariatric Surgery: A Meta-analysis. JAMA. 2016;315:150-63.

[19] Fu R, Zhang Y, Yu K, Mao D, Su H. Bariatric surgery alleviates depression in obese patients: A systematic review and meta-analysis. Obesity Research & Clinical Practice. 2022;16(1):10-6.

[20] Gill H, Kang S, Lee Y, Rosenblat JD, Brietzke E, Zuckerman H, et al. The long-term effect of bariatric surgery on depression and anxiety. J. Affect. Disord. 2019;246:886-94.

[21] de Zwaan M, Enderle J, Wagner S, Mühlhans B, Ditzen B, Gefeller O, et al. Anxiety and depression in bariatric surgery patients: A prospective, follow-up study using structured clinical interviews. Journal of Affective Disorders. 2011;133(1):61-8.

[22] Neovius M, Bruze G, Jacobson P, Sjöholm K, Johansson K, Granath F, et al. Risk of suicide and non-fatal self-harm after bariatric surgery: results from two matched cohort studies. Lancet Diabetes Endocrinol. 2018;6:197-207.

For peer review only - http://bmjopen.bmj.com/site/about/guidelines.xhtml

[23] Backman O, Stockeld D, Rasmussen F, Näslund E, Marsk R. Alcohol and substance abuse, depression and suicide attempts after Roux-en-Y gastric bypass surgery. Br J Surg. 2016;103:1336-42.

[24] Rogers CA, Reeves BC, Byrne J, Donovan JL, Mazza G, Paramasivan S, et al. Adaptation of the By-Band randomised clinical trial to By-Band-Sleeve to include a new intervention and maintain relevance of the study to practice. Br J Surg. 2017;104:1207-14.

[25] Rogers CA, Welbourn R, Byrne J, Donovan JL, Reeves BC, Wordsworth S, et al. The By-Band study: gastric bypass or adjustable gastric band surgery to treat morbid obesity: study protocol for a multi-centre randomised controlled trial with an internal pilot phase. Trials. 2014;15:53.

[26] Zigmond AS, Snaith RP. The hospital anxiety and depression scale. Acta Psychiatr Scand. 1983;67:361-70.

[27] Bjelland I, Dahl AA, Haug TT, Neckelmann D. The validity of the Hospital Anxiety and Depression Scale: An updated literature review. J. Psychosom. Res. 2002;52:69-77.

[28] Karlsson J, Taft C, Rydén A, Sjöström L, Sullivan M. Ten-year trends in health-related quality of life after surgical and conventional treatment for severe obesity: the SOS intervention study. Int J Obes (Lond). 2007;31:1248-61.

[29] Borisenko O, Colpan Z, Dillemans B, Funch-Jensen P, Hedenbro J, Ahmed AR. Clinical Indications, Utilization, and Funding of Bariatric Surgery in Europe. Obes Surg. 2015;25:1408-16.

[30] Burgmer R, Petersen I, Burgmer M, de Zwaan M, Wolf AM, Herpertz S. Psychological outcome two years after restrictive bariatric surgery. Obes Surg. 2007;17(6):785-91.

[31] Homer CV, Tod AM, Thompson AR, Allmark P, Goyder E. Expectations and patients' experiences of obesity prior to bariatric surgery: a qualitative study. BMJ Open. 2016;6:e009389.

For peer review only - http://bmjopen.bmj.com/site/about/guidelines.xhtml

[32] Raves DM, Brewis A, Trainer S, Han S-Y, Wutich A. Bariatric Surgery Patients' Perceptions of Weight-Related Stigma in Healthcare Settings Impair Post-surgery Dietary Adherence. Front Psychol. 2016;7:1497-.

[33] Lindekilde N, Gladstone BP, Lübeck M, Nielsen J, Clausen L, Vach W, et al. The impact of bariatric surgery on quality of life: a systematic review and meta-analysis. Obes Rev. 2015;16:639-51.

For peer review only

For peer review only - http://bmjopen.bmj.com/site/about/guidelines.xhtml

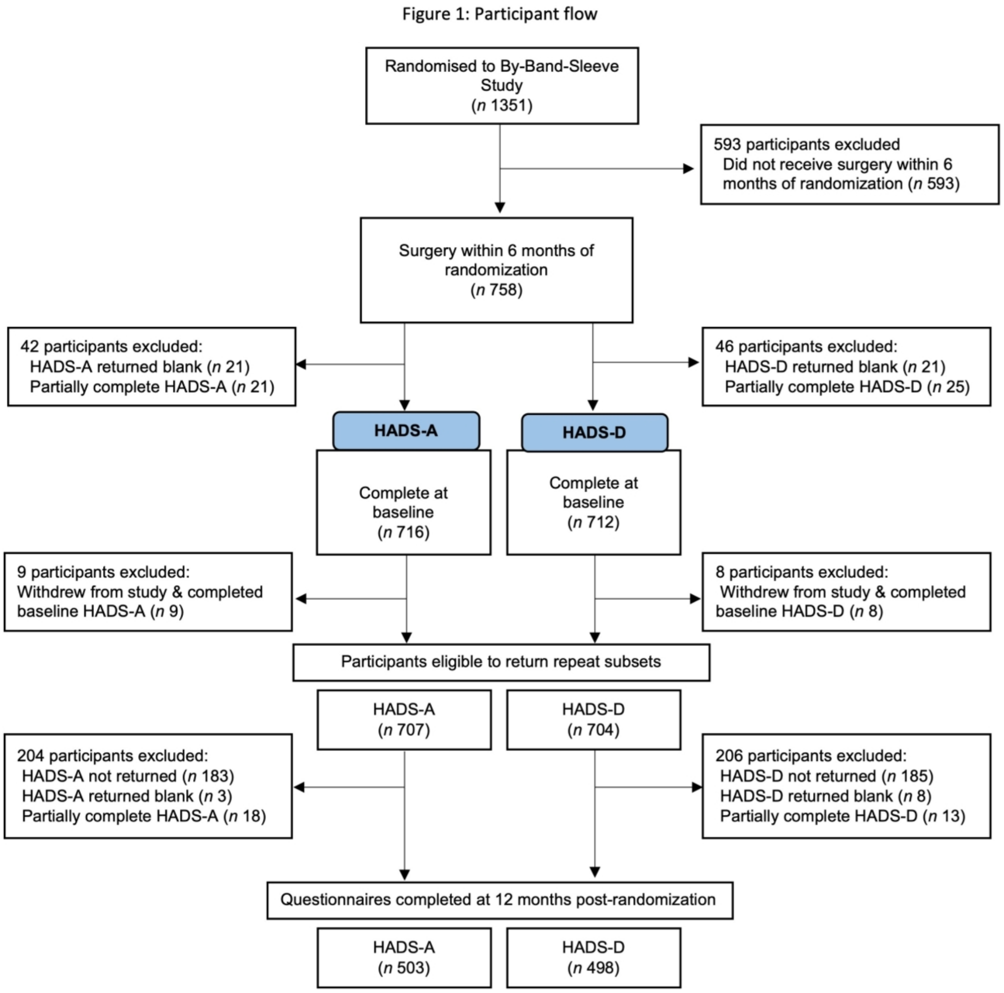

FIGURE 1: Flow Diagram
Flow diagram representing questionnaire sub-scale completion for the HADS amongst the sample obtained from the By-Band-Sleeve study.

192x192mm (300 x 300 DPI)

Figure 2: HADS Anxiety (HADS-A) and Depression (HADS-D)

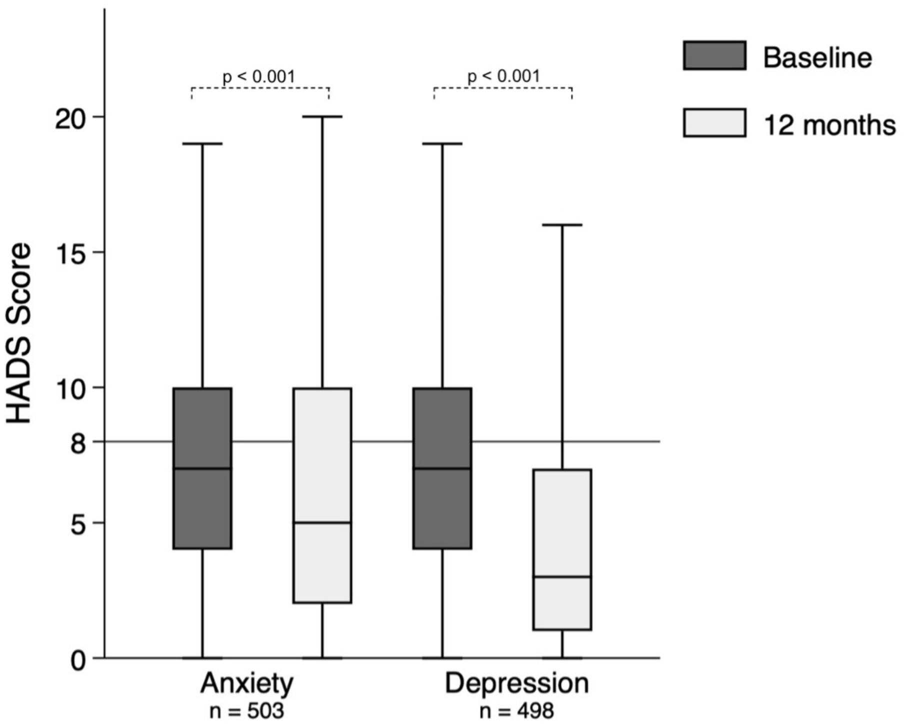

Total symptom scores for participants who completed HADS-A or HADS-D subscales at baseline (pre-randomisation) and 12 months post-randomisation. The horizontal black line at the HADS Score of 8 on the y axis represents the cut-off for clinical cases. For both anxiety and depression, there was a significant ($p < 0.001$) decrease in median HADS score at 12 months post-randomisation.

159x136mm (220 x 220 DPI)

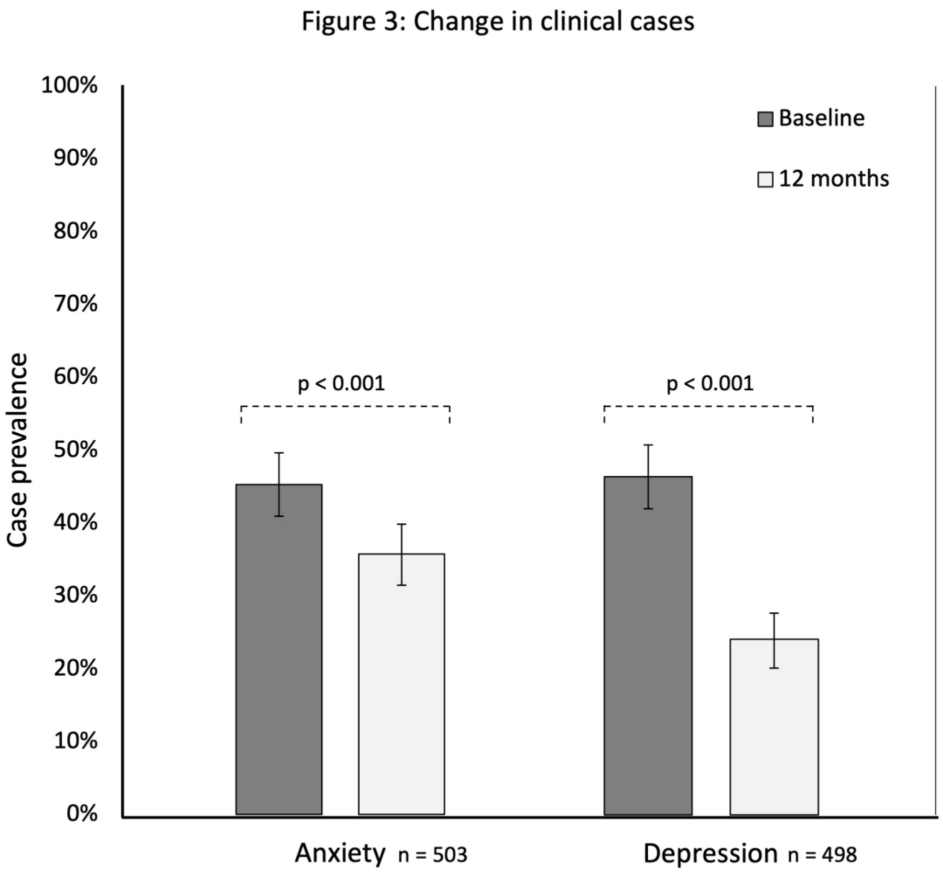

FIGURE 3: Change in clinical cases
Proportion of possible clinical cases (HADS-A/D >8) at baseline and 12 months post-randomisation. Each bar represents the case prevalence (with associated 95% confidence interval) for anxiety and depression. At 12 months post-randomisation, there was a reduction in the proportion of individuals with anxiety (9.5% decrease, 95% CI -14.3 to -4.8%, $p < 0.001$) and depression (22.3% decrease, 95% CI -27.0 to -17.6%, $p < 0.001$).

165x149mm (300 x 300 DPI)

Appendix A

Anxiety and depression following bariatric surgery

Supplementary Results

Supplementary results

List of tables

		Page
Table 1	Baseline characteristics by repeat HADS questionnaire return status	2
Table 2	Baseline characteristics by repeat HADS-A questionnaire return status	3
Table 3	Baseline characteristics by repeat HADS-D questionnaire return status	4

For peer review only

For peer review only - http://bmjopen.bmj.com/site/about/guidelines.xhtml

Appendix A

Anxiety and depression following bariatric surgery

Supplementary Results

Table 1
Participant baseline characteristics by repeat HADS questionnaire return status (Continuous variables)

		12 months post-randomisation HADS-A return			
		**Yes** (N = 503)	**No** (N = 204)	**Difference** (95% CI)	**p-value**[1]
**Age** (years)	Mean (95% CI)	48.7 (47.8 to 49.6)	44.6 (43.1 to 46.0)	4.1 (2.5 to 5.8)	<0.001
**BMI** (kg/m2)	Mean (95% CI)	46.3 (45.7 to 46.8)	46.8 (45.8 to 47.7)	-0.5 (-1.6 to 0.6)	0.379
**Time from randomisation to surgery** (days)	Mean (95% CI)	92.6 (88.7 to 96.5)	91.5 (85.5 to 97.6)	1.1 (-6.1 to 8.3)	0.767
		**12 months post-randomisation HADS-D return**			
		**Yes** (N = 498)	**No** (N = 206)	**Difference** (95% CI)	**p-value**[1]
**Age** (years)	Mean (95% CI)	48.8 (47.9 to 49.7)	44.5 (43.1 to 45.9)	4.3 (2.7 to 6.0)	<0.001
**BMI** (kg/m2)	Mean (95% CI)	46.3 (45.7 to 46.9)	46.8 (45.8 to 47.7)	-0.5 (-1.6 to 0.6)	0.370
**Time from randomisation to surgery** (days)	Mean (95% CI)	92.9 (89.0 to 96.8)	91.8 (85.8 to 97.8)	1.1 (-6.1 to 8.3)	0.767

[1] p-value obtained from paired sample t-test statistic for mean difference by HADS return status

For peer review only - http://bmjopen.bmj.com/site/about/guidelines.xhtml

Appendix A

Anxiety and depression following bariatric surgery
Supplementary Results

Table 2
Participant characteristics by repeat HADS-A questionnaire return status (categorical variables)

		12 months post-randomisation HADS-A return			
		**Yes** (%) N = 503 (71.15)	**No** (%) N = 204 (28.85)	**Odds Ratio for non-return**[2] (95% CI)	**p-value**[3]
**Sex**	Male (*n* 178)	126 (70.79)	52 (29.21)	1.00	0.903
	Female (*n* 529)	377 (71.27)	152 (28.73)	0.98 (0.67 to 1.42)	
**Ethnicity**	White (*n* 658)	472 (71.73)	186 (28.27)	1.00	0.217
	Other ethnic group[4] (*n* 49)	31 (63.27)	18 (36.73)	1.47 (0.80 to 2.70)	
**Marital status**	Married or civil partnership (*n* 395)	280 (70.89)	115 (29.11)	1.00	0.926
	Co-habiting (*n* 84)	62 (73.81)	22 (26.19)	0.86 (0.51 to 1.47)	
	Single (*n* 132)	92 (69.70)	40 (30.30)	1.06 (0.69 to 1.63)	
	Divorced, Separated, or Widowed[5] (*n* 96)	69 (71.88)	27 (28.12)	0.95 (0.58 to 1.56)	
**Smoking status**	Never smoked (*n* 317)	226 (71.29)	91 (28.71)	1.00	0.339
	Ex-smoker (*n* 343)	248 (72.30)	95 (27.70)	0.95 (0.68 to 1.33)	
	Current smoker (*n* 47)	29 (61.70)	18 (38.30)	1.54 (0.82 to 2.91)	
**Employment status**	Employed (*n* 455)	326 (71.65)	129 (28.35)	1.00	0.002
	Not in employment or student[6] (*n* 190)	123 (64.74)	67 (35.26)	1.38 (0.96 to 1.97)	
	Retired (*n* 62)	54 (87.10)	8 (12.90)	0.37 (0.17 to 0.81)	
**Income band**	≤10,000 (*n* 84)	59 (70.24)	25 (29.76)	1.00	0.247
	10,001 to 30,000 (*n* 289)	212 (73.36)	77 (26.64)	0.86 (0.50 to 1.46)	
	30,001 to 50,000 (*n* 150)	105 (70.00)	45 (30.00)	1.01 (0.56 to 1.81)	
	≥50,001 (*n* 91)	69 (75.82)	22 (24.18)	0.75 (0.38 to 1.47)	
	Not disclosed (*n* 92)	57 (61.96)	35 (38.04)	1.45 (0.77 to 2.72)	

2 Odds ratio for questionnaire non-return calculated using logistic regression
3 p-value obtained from likelihood ratio chi-square test
For calculation of Odds ratios associated with questionnaire non-return, categories with sub-groups containing ≤5% (*n* 35/707) total respondents were merged with the next largest sub-group to avoid data sparsity: **4** Includes participants who identified as African or Caribbean (*n* 28/707), Mixed ethnic group (*n* 13/707), Asian (*n* 3/707), or Other (*n* 5/707). **5** Participants who identified as separated (*n* 21/703) and widowed (*n* 11/703) combined with those who identified as divorced (*n* 64/703) as next largest category. **6** Participants who identified as students (*n* 6/703) combined with those not in employment (*n* 183/703)

For peer review only - http://bmjopen.bmj.com/site/about/guidelines.xhtml

Appendix A

Anxiety and depression following bariatric surgery
Supplementary Results

Table 3
Baseline characteristics by repeat HADS-D questionnaire return status (categorical variables)

		12 months post-randomisation HADS-D return			
		**Yes** (%) N = 498 (70.74)	**No** (%) N = 206 (29.26)	**Odds Ratio for non-return**[2] (95% CI)	**p-value**[3]
**Sex**	Male (*n* 178)	126 (70.79)	52 (29.21)	1.00	0.987
	Female (*n* 526)	372 (70.72)	154 (29.28)	0.98 (0.69 to 1.46)	
**Ethnicity**	White (*n* 655)	468 (71.45)	187 (28.55)	1.00	0.139
	Other ethnic group[4] (*n* 49)	30 (61.22)	19 (38.78)	1.59 (0.87 to 2.89)	
**Marital status**	Married or civil partnership (*n* 390)	273 (70.00)	117 (30.00)	1.00	0.592
	Co-habiting (*n* 84)	64 (76.19)	20 (23.81)	0.73 (0.42 to 1.26)	
	Single (*n* 132)	90 (68.18)	42 (31.82)	1.09 (0.71 to 1.67)	
	Divorced, Separated, or Widowed[5] (*n* 98)	71 (72.45)	27 (27.55)	0.89 (0.54 to 1.45)	
**Smoking status**	Never smoked (*n* 317)	225 (70.98)	92 (29.02)	1.00	0.566
	Ex-smoker (*n* 340)	243 (71.47)	97 (28.53)	0.98 (0.70 to 1.37)	
	Current smoker (*n* 47)	30 (63.83)	17 (36.17)	1.39 (0.73 to 2.63)	
**Employment status**	Employed (*n* 451)	322 (71.40)	129 (28.60)	1.00	0.003
	Not in employment or student[6] (*n* 190)	122 (64.21)	68 (35.79)	1.39 (0.97 to 1.99)	
	Retired (*n* 63)	54 (85.71)	9 (14.29)	0.40 (0.33 to 0.49)	
**Income band**	<u><</u>10,000 (*n* 86)	59 (68.60)	27 (31.40)	1.00	0.108
	10,001 to 30,000 (*n* 286)	207 (72.38)	79 (27.62)	0.83 (0.49 to 1.41)	
	30,001 to 50,000 (*n* 149)	103 (69.13)	46 (30.87)	0.98 (0.55 to 1.73)	
	<u>></u>50,001 (*n* 91)	72 (79.12)	19 (20.88)	0.58 (0.29 to 1.14)	
	Not disclosed (*n* 91)	56 (61.54)	35 (38.46)	1.37 (0.73 to 2.54)	

[2] Odds ratio for questionnaire non-return obtained using logistic regression
[3] P-value obtained from likelihood ratio chi-square test
For calculation of Odds ratios associated with questionnaire non-return, categories with sub-groups containing <u><</u>5% (*n* 35/707) total respondents were merged into the next largest sub-group to avoid data sparsity: [4] Includes participants who identified as African or Caribbean (*n* 28/704), Mixed ethnic group (*n* 13/707), Asian (*n* 3/707), or Other (*n* 5/707). [5] Participants who identified as separated (*n* 21/703) and widowed (*n* 11/703) combined with those who identified as divorced (*n* 66/703) as next largest category. [6] Participants who identified as students (*n* 7/703) combined with those not in employment (*n* 183/703).

For peer review only - http://bmjopen.bmj.com/site/about/guidelines.xhtml

STROBE: Anxiety and depression following bariatric surgery

STROBE Statement

	Item No	Recommendation	Page No
**Title and abstract**	1	(*a*) Indicate the study's design with a commonly used term in the title or the abstract	1
		(*b*) Provide in the abstract an informative and balanced summary of what was done and what was found	4, 5
**Introduction**			
Background/rationale	2	Explain the scientific background and rationale for the investigation being reported	7, 8
Objectives	3	State specific objectives, including any prespecified hypotheses	4, 5, 8, 9
**Methods**			
Study design	4	Present key elements of study design early in the paper	8, 10
Setting	5	Describe the setting, locations, and relevant dates, including periods of recruitment, exposure, follow-up, and data collection	10, 11, 12, 13
Participants	6	(*a*) Give the eligibility criteria, and the sources and methods of selection of participants	10
Variables	7	Clearly define all outcomes, exposures, predictors, potential confounders, and effect modifiers. Give diagnostic criteria, if applicable	10, 11
Data sources/ measurement	8*	For each variable of interest, give sources of data and details of methods of assessment (measurement). Describe comparability of assessment methods if there is more than one group	10, 11, 12
Bias	9	Describe any efforts to address potential sources of bias	10, 11
Study size	10	Explain how the study size was arrived at	10
Quantitative variables	11	Explain how quantitative variables were handled in the analyses. If applicable, describe which groupings were chosen and why	11, 12
Statistical methods	12	(*a*) Describe all statistical methods, including those used to control for confounding	11, 12
		(*b*) Describe any methods used to examine subgroups and interactions	11, 12
		(*c*) Explain how missing data were addressed	11, 12
		(*d*) If applicable, describe analytical methods taking account of sampling strategy	11, 12
		(*e*) Describe any sensitivity analyses	11, 12
**Results**			
Participants	13*	(a) Report numbers of individuals at each stage of study—eg numbers potentially eligible, examined for eligibility, confirmed eligible, included in the study, completing follow-up, and analysed	14, 15, Figure 1 [Flow diagram]
		(b) Give reasons for non-participation at each stage	14, 15, Figure 1
		(c) Consider use of a flow diagram	Figure 1

For peer review only - http://bmjopen.bmj.com/site/about/guidelines.xhtml

STROBE: Anxiety and depression following bariatric surgery

Descriptive data	14*	(a) Give characteristics of study participants (eg demographic, clinical, social) and information on exposures and potential confounders	15 Table 1 (All) Appendix A (Non-responders)
		(b) Indicate number of participants with missing data for each variable of interest	15, 18, Table 1, Figure 1
Outcome data	15*	Report numbers of outcome events or summary measures	14, 15, 16, 17, 18 Table 2, Table 3, Figure 2, Figure 3, Appendix A
Main results	16	(*a*) Give unadjusted estimates and, if applicable, confounder-adjusted estimates and their precision (eg, 95% confidence interval). Make clear which confounders were adjusted for and why they were included	14, 17, Table 2
		(*b*) Report category boundaries when continuous variables were categorized	15, Table 1, Table 2, Table 3
		(*c*) If relevant, consider translating estimates of relative risk into absolute risk for a meaningful time period	N/A
Other analyses	17	Report other analyses done—eg analyses of subgroups and interactions, and sensitivity analyses	18, Appendix A
**Discussion**			
Key results	18	Summarise key results with reference to study objectives	19
Limitations	19	Discuss limitations of the study, taking into account sources of potential bias or imprecision. Discuss both direction and magnitude of any potential bias	20, 21
Interpretation	20	Give a cautious overall interpretation of results considering objectives, limitations, multiplicity of analyses, results from similar studies, and other relevant evidence	19, 20
Generalisability	21	Discuss the generalisability (external validity) of the study results	20
**Other information**			
Funding	22	Give the source of funding and the role of the funders for the present study and, if applicable, for the original study on which the present article is based	28

*Give information separately for exposed and unexposed groups.

Note: An Explanation and Elaboration article discusses each checklist item and gives methodological background and published examples of transparent reporting. The STROBE checklist is best used in conjunction with this article (freely available on the Web sites of PLoS Medicine at http://www.plosmedicine.org/, Annals of Internal Medicine at http://www.annals.org/, and Epidemiology at http://www.epidem.com/). Information on the STROBE Initiative is available at www.strobe-statement.org.

For peer review only

BMJ Open

Prevalence and short-term change in symptoms of anxiety and depression following bariatric surgery: a prospective cohort study

Journal:	*BMJ Open*
Manuscript ID	bmjopen-2022-071231.R1
Article Type:	Original research
Date Submitted by the Author:	17-May-2023
Complete List of Authors:	Band-Sleeve Trial Management Group, (Group Authorship); By-Band-Sleeve Collaborating group<br>Gibb, Jonathan; University of Bristol,
**Primary Subject Heading**:	Mental health
Secondary Subject Heading:	Surgery, Nutrition and metabolism, Epidemiology
Keywords:	Depression & mood disorders < PSYCHIATRY, Anxiety disorders < PSYCHIATRY, Adult surgery < SURGERY, EPIDEMIOLOGIC STUDIES

SCHOLARONE™ Manuscripts

I, the Submitting Author has the right to grant and does grant on behalf of all authors of the Work (as defined in the below author licence), an exclusive licence and/or a non-exclusive licence for contributions from authors who are: i) UK Crown employees; ii) where BMJ has agreed a CC-BY licence shall apply, and/or iii) in accordance with the terms applicable for US Federal Government officers or employees acting as part of their official duties; on a worldwide, perpetual, irrevocable, royalty-free basis to BMJ Publishing Group Ltd ("BMJ") its licensees and where the relevant Journal is co-owned by BMJ to the co-owners of the Journal, to publish the Work in this journal and any other BMJ products and to exploit all rights, as set out in our licence.

The Submitting Author accepts and understands that any supply made under these terms is made by BMJ to the Submitting Author unless you are acting as an employee on behalf of your employer or a postgraduate student of an affiliated institution which is paying any applicable article publishing charge ("APC") for Open Access articles. Where the Submitting Author wishes to make the Work available on an Open Access basis (and intends to pay the relevant APC), the terms of reuse of such Open Access shall be governed by a Creative Commons licence – details of these licences and which Creative Commons licence will apply to this Work are set out in our licence referred to above.

Other than as permitted in any relevant BMJ Author's Self Archiving Policies, I confirm this Work has not been accepted for publication elsewhere, is not being considered for publication elsewhere and does not duplicate material already published. I confirm all authors consent to publication of this Work and authorise the granting of this licence.

For peer review only

Prevalence and short-term change in symptoms of anxiety and depression following bariatric surgery: a prospective cohort study

On behalf of The By-Band-Sleeve Collaborating Group

Corresponding author

Dr Jonathan Gibb

Centre for Academic Mental Health, Population Health Sciences

Bristol Medical School, University of Bristol

Oakfield Grove, Clifton, Bristol BS8 2BN

Jonathan.gibb@bristol.ac.uk

+44 (0)117 428 2489

Word Count (Excluding title page, abstract, references, figures and table): **3,292**

For peer review only - http://bmjopen.bmj.com/site/about/guidelines.xhtml

ABSTRACT

Objectives: Bariatric surgery is an effective treatment for severe obesity that leads to significant physical health improvements. Few studies have prospectively described the short-term impact of surgery on mental health using standardised case-finding measures for anxiety or depressive disorders. This study describes the prevalence and short-term course of these conditions following surgery.

Design: Prospective observational cohort study.

Setting: 12 National Health Service centres in England.

Participants: Participants studied took part in the By-Band-Sleeve study, a multi-centre randomised controlled trial evaluating the surgical management of severe obesity. We included participants who had undergone surgery (Gastric Bypass, Gastric Band or Sleeve Gastrectomy) within 6 months of randomisation.

Primary and secondary outcome measures: Anxiety and depression were assessed using the Hospital Anxiety and Depression Scale (HADS) at baseline and 12 months post-randomisation. Sociodemographic variables collected at pre-randomisation included Body Mass Index, Age, Sex, Ethnicity, Marital Status, Tobacco use, Employment Status, and Income Band.

Results: In our sample of 758 participants, 94.5% (n 716) and 93.9% (n 712) had completed baseline anxiety (HADS-A) and depression (HADS-D) subscales. At pre-randomisation 46.1% (n 330/716, 95% CI 42.4 to 49.7%) met clinical case criteria for

For peer review only - http://bmjopen.bmj.com/site/about/guidelines.xhtml

anxiety and 48.2% (n 343/712, 95% CI 44.5 to 51.8%) for depression. Among participants returning completed 12 months post-randomisation questionnaires (HADS-A n 503/716, HADS-D n 498/712), there was a significant reduction in the proportion of clinical cases with anxiety (-9.5%, 95% CI -14.3 to -4.8% $p < 0.001$) and depression (-22.3%, 95% CI -27.0 to -17.6% $p < 0.001$).

Conclusions: Almost half of people undergoing bariatric surgery had underlying anxiety or depressive symptoms. In the short term, these symptoms appear to substantially improve. Future work must identify whether these effects are sustained beyond the first post-randomisation year.

Trial registration: The By-Band-Sleeve Study is registered with ClinicalTrials.gov database (NCT02841527) and ISRCTN registry (ISRCTN00786323).

For peer review only - http://bmjopen.bmj.com/site/about/guidelines.xhtml

Strengths and limitations of this study

- A validated self-report measure, the Hospital Anxiety and Depression Scale (HADS), was used to detect anxiety and depressive disorders.

- Participants were recruited from the largest randomised controlled trial, to date, in bariatric surgery (The By-Band-Sleeve Study) from multiple NHS surgical centres in England.

- Although participants were re-assessed using the HADS at one-year post-randomisation, the total follow-up period from surgery was relatively short. It is possible that these changes were not maintained after the first post-operative year.

- With respect to surgical procedure, participants were analysed as a whole group, rather than being stratified by surgery type (Gastric Bypass, Gastric Band or Sleeve Gastrectomy).

For peer review only

For peer review only - http://bmjopen.bmj.com/site/about/guidelines.xhtml

INTRODUCTION

Obesity and common mental disorders, such as anxiety and depression, contribute greatly to global disease burden and pose significant public health challenges [1, 2, 3]. There has been a recent focus on understanding the relationship between obesity and common mental disorders. Systematic reviews and meta-analyses of longitudinal studies have found a bi-directional relationship between having obesity and developing a depressive disorder [4, 5] across both sexes, however a recently updated review found an elevated risk only among females [6]. Whilst there have been fewer longitudinal studies assessing the relationship between obesity and anxiety disorders, there is evidence of a positive association between the two conditions [7, 8]. These findings have coincided with a growing body of research studying the potential shared neurobiological (the role of prolonged inflammatory changes, cortisol dysregulation, metabolic dysfunction, and disrupted cellular signalling) pathways between obesity, anxiety states, and depression which may eventually give rise to a better understanding of these common co-morbidities [9, 10, 11].

When people with severe or complex obesity (Body Mass Index ≥40kg/m^2 or ≥35kg/m^2 with a significant co-morbidity) are unable to lose weight, and have attempted all relevant non-surgical measures, current guidelines in the United Kingdom (UK) recommend that bariatric surgery should be offered [12, 13]. Compared to non-surgical management, bariatric surgery has been shown to be an effective treatment for severe obesity and is associated with gains in overall life expectancy alongside increased remission rates of rates of obesity-related co-morbidities, such as type 2 diabetes mellitus [14, 15, 16]. They were 39,054 recorded operations within

For peer review only - http://bmjopen.bmj.com/site/about/guidelines.xhtml

the UK National Bariatric Surgery Registry (NSBR) between 2013 to 2018. The Roux-en-Y gastric bypass was the most common bariatric surgical procedure (*n* 19,104, 48.9%), followed by sleeve gastrectomy (*n* 13,841, 35.4%) then the gastric band (*n* 4,499, 11.5%) [17].

Previous research suggests that people who undergo bariatric surgery have higher rates of pre-operative depression compared to people with obesity who do not undergo surgery [18]. A 2016 meta-analysis of the international literature estimated that up to 23% of patients have a mood disorder at the time of surgery [19], with the pooled estimate for depression being 19% [95% CI 14 to 25%, 34 studies, *N* 12,009/51,908 participants] and anxiety 12% [95% CI 6 to 20%, 22 studies, *N* 10,515/38,459 participants]. In the short-term following surgery, there appears to be a reduction in the prevalence and severity of depression [20] however there remains uncertainty around the course of anxiety symptoms [20, 21, 22]. Previous literature on the mental health status of bariatric surgical recipients has often been limited due to the use of uncertain diagnostic criteria, measures for common mental disorders which do not address anxiety symptoms separately from depressive symptoms, and a lack of reporting on symptom severity [21]. As rates of severe and complex obesity increase, there is a clear need to better understand the prevalence and course of common mental health problems following surgery. This is particularly timely as recent research has found an increased risk of self-harm among those who undergo weight loss surgery [23, 24] compared to people with obesity who do not.

This paper presents findings from an analysis of data from the largest randomised controlled trial to date of bariatric surgery – the By-Band-Sleeve study [25, 26]. The

For peer review only - http://bmjopen.bmj.com/site/about/guidelines.xhtml

study is comparing the clinical and cost effectiveness of gastric banding (Band), laparoscopic gastric bypass (Bypass) or sleeve gastrectomy (Sleeve) which are the three most common surgical treatments for severe obesity. The objectives of this sub-study were to describe the prevalence, and severity, of anxiety and depressive symptoms among participants who underwent any type of bariatric surgery within 6 months of randomisation at baseline (pre-randomisation) and following surgery at 12 months post-randomisation.

For peer review only

For peer review only - http://bmjopen.bmj.com/site/about/guidelines.xhtml

METHOD

Participants

Participants were included in this sub-study if they had taken part in the By-Band-Sleeve study, had undergone surgery (irrespective of procedure type) within six months of randomisation, and had completed the Hospital Anxiety and Depression Scale after informed consent and before randomisation. By-Band-Sleeve study exclusion criteria included: previous gastric surgery for severe and complex obesity, previous abdominal surgery or gastro-intestinal conditions that precludes the surgical intervention, large abdominal ventral hernia or hiatus hernia >5cm, pregnancy, clinical conditions (such as Crohn's disease, liver cirrhosis and portal hypertension), known silicone allergy, or active participation in another interventional research study which may interfere with the By-Band-Sleeve study.

To understand the effect of surgery on mental health, participants were excluded if they had not undergone surgery within six months of randomisation. This cut-off of six months from enrolment was selected a priori in the event of participants waiting a prolonged time for surgery to take place (for example, due to the ongoing impact of the COVID-19 pandemic on elective surgery), which may have reduced the accuracy and relevance of baseline assessment of pre-operative mental health status. In total, 1,351 participants were randomised to the By-Band-Sleeve study and in this paper, we report on the mental health outcomes of the 758 eligible participants.

Primary measure

The Hospital Anxiety and Depression Scale (HADS) was completed at pre-randomisation (study enrolment or 'baseline') and at 12 months post-randomisation.

For peer review only - http://bmjopen.bmj.com/site/about/guidelines.xhtml

HADS is a 14-item questionnaire (7 questions for anxiety 'A' and 7 questions for depressive 'D' symptoms), which asks the participant to score each item between 0 to 3 based on their level of agreement. A sub-scale total score of less than 8 is considered normal, 8 to 10 suggestive of possible anxiety or depressive disorder, and a score greater than 11 is suggestive of a probable disorder [27]. Previous research has determined that a sub-scale score of $\geq$8 represents the optimal case cut-off for clinical anxiety and depressive disorders, in terms of the balance between sensitivity and specificity [28].

Secondary measures

Baseline characteristics and demographic data for participants were collected on study enrolment. These included Body Mass Index (BMI), Age, Sex, Ethnicity, Marital Status, Tobacco use, Employment Status, and Income Band. Time from randomisation to surgery and number of centres participating were described.

Statistical analysis

Analyses were undertaken using Stata Version 16. Returned HADS questionnaires were assessed for completion of the 7-item anxiety (HADS-A) and depression (HADS-D) subscales. Participants who fully completed either subscale had a total symptom score calculated. The proportions of participants who met case criteria for possible anxiety and depression (defined as HADS-A/D $\geq$8) were described alongside baseline sociodemographic variables. The median symptom score (and interquartile range) was calculated for participants who had completed a subscale at both baseline and 12 months post-randomisation. The Wilcoxon signed-rank test was used to assess the statistical significance of any change in median symptom

For peer review only - http://bmjopen.bmj.com/site/about/guidelines.xhtml

score. The change in proportions of participants with possible depression or anxiety at pre-randomisation compared to 12 months post-randomisation was calculated alongside 95% confidence intervals. McNemar's chi-squared test was used to compare paired prevalence of anxiety and depression at baseline and 12 months post-randomisation.

Missing data and loss to follow-up

A complete case analysis was undertaken in which participants with fully completed HADS-A or HADS-D questionnaire subscales were included in the analysis. The characteristics of participants who did not return completed questionnaires at 12 months post-randomisation was compared to returners with respect to baseline symptom scores, proportion of clinical cases, and sociodemographic variables. For categorical variables, cross-tabulation was used to compare the distribution of baseline characteristics by repeat subscale return status. Odds ratios (with 95% confidence intervals) for questionnaire return status were calculated using logistic regression for each categorical variable. For continuous variables, which were normally distributed, a two-sample t-test was used to compare whether the mean value (such as BMI, age, and time from randomisation to surgery) differed by return status.

Ethical approval

The By-Band-Sleeve study gained National Health Service (NHS) ethics approval from the Southwest Frenchay Research Ethics Committee (REC No: 11/SW/0248) in 2011. The study is sponsored by the University of Bristol and was granted Health Research Authority (HRA) Approval in 2017. The By-Band-Sleeve Study is

For peer review only - http://bmjopen.bmj.com/site/about/guidelines.xhtml

registered with the National Institutes of Health ClinicalTrials.gov database (NCT02841527) and ISRCTN registry (ISRCTN00786323).

Patient and public involvement

This sub-study features data obtained from participants who took part in the By-Band-Sleeve study. Patients and public were involved in By-Band-Sleeve Study throughout the design and conduct of the trial. Patient representatives on the Trial Management Group contributed towards the writing of this manuscript and are recognised as co-authors. The results of this sub-study will be disseminated through the By-Band-Sleeve Patient and Public Involvement Group and summarised, for a non-specialist audience, on the study (www.bybandsleevestudy.blogs.bristol.ac.uk) webpage following publication.

For peer review only - http://bmjopen.bmj.com/site/about/guidelines.xhtml

RESULTS

Seven hundred and fifty-eight By-Band-Sleeve study participants who had undergone surgery at the time of undertaking this work and who were within six months of randomisation were included [***Figure 1***]. Participants were recruited between January 2013 and September 2019 from 12 NHS surgical centres in England. Demographic characteristics by baseline (pre-randomisation) total HADS scores (normal, possible, probable disorder) are displayed in [***Table 1***]. At the point of randomisation, the mean age of participants was 47.8 (Standard Deviation, *SD* 10.6) years and the mean BMI was 46.3 (SD 6.7) kg/m^2. In total 570/758 (75.2%) participants were female.

Participant characteristics by baseline HADS scores

Of the 758 participants, 737 (97.2%) had returned baseline HADS questionnaires. For the subscales, baseline completion for the HADS-A was 94.5% (716/758) and 93.9% (712/758) for the HADS-D. The median symptom score for both baseline HADS-A and HADS-D was 7 (IQR 4 – 10). The proportion of individuals meeting case criteria for a possible, or probable, anxiety disorder was 46.1% (*n* 330/716, 95% CI 42.4 to 49.7%) and 48.2% (*n* 343/712, 95% CI 44.5 to 51.8%) for depression. Time from randomisation to surgery varied with a mean time of 92.1 (SD 44.4) days and was similar across the groups when stratified by baseline anxiety and depression status [***Table 1***].

For peer review only - http://bmjopen.bmj.com/site/about/guidelines.xhtml

Table 1: Demographic data by baseline HADS scores

		**HADS-A** (n = 716) Anxiety case category			**HADS-D** (n = 712) Depression case category		
		**Nil**	**Possible**	**Probable**	**Nil**	**Possible**	**Probable**
		<8	8 - 10	≥11	<8	8 - 10	≥11
**Total**	n (%)	386 (53.9)	159 (22.2)	171 (23.9)	369 (51.8)	181 (25.4)	162 (22.8)
**Time to surgery** (days)	mean (SD)	93.2 (45.3)	93.2 (44.3)	88.2 (41.5)	93.7 (46.5)	92.7 (43.8)	88.9 (39.7)
**Age** (years)	mean (SD)	48.1 (10.5)	46.8 (10.7)	46.3 (10.6)	47.8 (10.5)	47.7 (11.2)	46.4 (10.2)
**BMI** (kg/m^2)	mean (SD)	46.0 (6.4)	47.0 (7.1)	46.9 (7.0)	46.2 (6.6)	46.5 (6.9)	46.8 (7.0)
**Sex** n (%)	Male	102 (56.4)	42 (23.2)	37 (20.4)	90 (49.7)	50 (27.6)	41 (22.7)
	Female	284 (53.1)	117 (21.9)	134 (25.1)	279 (52.5)	131 (24.7)	121 (22.8)
**Ethnicity** n (%)	White	359 (53.9)	150 (22.5)	157 (23.6)	340 (51.4)	173 (26.1)	149 (22.5)
	African or Caribbean	17 (60.7)	6 (21.4)	5 (17.9)	17 (60.7)	4 (14.3)	7 (25.0)
	Mixed	7 (50.0)	2 (14.3)	5 (35.7)	8 (57.1)	1 (7.1)	5 (35.7)
	Asian	0 (0.0)	0 (0.0)	3 (100.0)	2 (66.7)	0 (0.0)	1 (33.3)
	Other	3 (60.0)	1 (20.0)	1 (20.0)	2 (40.0)	3 (60.0)	0 (0.0)
**Marital status** n (%)	Married or civil partnership	223 (55.8)	83 (20.8)	94 (23.5)	205 (52.0)	95 (24.1)	94 (23.9)
	Co-habiting	50 (58.8)	19 (22.4)	16 (18.8)	45 (52.9)	22 (25.9)	18 (21.2)
	Single	69 (51.5)	32 (23.9)	33 (24.6)	70 (52.2)	34 (25.4)	30 (22.4)
	Divorced	28 (43.1)	19 (29.2)	18 (27.7)	35 (52.2)	19 (28.4)	13 (19.4)
	Separated	11 (52.4)	5 (23.8)	5 (23.8)	11 (52.4)	7 (33.3)	3 (14.3)
	Widowed	5 (45.5)	1 (9.1)	5 (45.5)	3 (27.3)	4 (36.4)	4 (36.4)
**Smoking status** n (%)	Never smoked	181 (56.4)	61 (19.0)	79 (24.6)	156 (48.8)	83 (25.9)	81 (25.3)
	Ex-smoker	181 (52.0)	90 (25.9)	77 (22.1)	189 (54.8)	83 (24.1)	73 (21.2)
	Current smoker	24 (51.1)	8 (17.0)	15 (31.9)	24 (51.1)	15 (31.9)	8 (17.0)
**Employment status** n (%)	Employed	285 (62.1)	93 (20.3)	81 (17.7)	267 (58.8)	110 (24.2)	77 (17.0)
	Not in employment	62 (33.0)	50 (26.6)	76 (40.4)	70 (37.2)	51 (27.1)	67 (35.6)
	Student	4 (57.1)	2 (28.6)	1 (14.3)	4 (57.1)	2 (28.6)	1 (14.3)
	Retired	35 (56.5)	14 (22.6)	13 (21.0)	28 (44.4)	18 (28.6)	17 (27.0)
**Income band (GBP)** n (%)	≤ £10,000	28 (31.5)	29 (32.6)	32 (36.0)	35 (35.6)	30 (33.3)	28 (31.1)
	10,001-30,000	159 (52.5)	60 (20.6)	73 (25.0)	156 (54.0)	69 (23.9)	64 (22.2)
	30,001-50,000	89 (59.3)	36 (24.0)	25 (16.7)	80 (53.7)	41 (27.5)	28 (18.8)
	50,001-70,000	44 (68.8)	12 (18.8)	8 (12.5)	38 (60.3)	15 (23.8)	10 (15.9)

	> £70,001	18 (66.7)	6 (17.2)	3 (11.1)	20 (71.4)	6 (21.7)	2 (7.1)
	Not disclosed	47 (50.5)	16 (17.2)	30 (32.3)	42 (45.7)	20 (21.7)	30 (32.6)
	Missing	1 (100.0)	0 (0.0)	0 (0.0)	1 (100.0)	0 (0.0)	0 (0.0)

Prevalence of anxiety and depression at 12 months post-randomisation

At 12 months post-randomisation, nine of the participants who had completed baseline HADS-A and eight of the participants who had completed baseline HADS-D had withdrawn or died. [***Figure 1***]. After accounting for these individuals, the proportion of questionnaires returned complete was 71.1% (*n* 503/707) for the HADS-A and 70.7% (*n* 498/704) for the HADS-D. The median HADS score decreased from 7 at baseline to 5 (IQR 2 – 10) for anxiety and to 3 (IQR 1 – 7) for depression at 12 months post-randomisation among participants who completed questionnaires at both timepoints [***Table 2***]. There was a statistically significant (p < 0.001) decrease in both HADS-A and HADS-D scores [***Figure 2***]. This was coupled with a significant reduction in the proportion of participants meeting caseness for anxiety (9.5% decrease, 95% CI -14.3 to -4.8%, p < 0.001) and depression (22.3% decrease, 95% CI -27.0 to -17.6%, p < 0.001) at 12 months post-randomisation when compared to baseline [***Figure 3***].

Whilst the overall proportion of cases of anxiety and depression decreased, the mental health of a small number of participants appeared to decline over the course of the 12-month follow-up, with 4.4% (*n* 22/498) of participants developing possible depression and 9.2% (*n* 46/503) developing a possible anxiety disorder [***Supplementary Table 1***].

For peer review only - http://bmjopen.bmj.com/site/about/guidelines.xhtml

Table 2: Change in HADS scores from baseline to 12 months post-randomisation						
				Proportion		
		**Median score** (IQR)	**Cases** (%)	**Change** (%)	**95% CI** (%)	**P-value**
**HADS-A** Anxiety (*n* 503)	Baseline	7 (4 – 10)	45.3	-9.5	-14.3 to -4.8	<0.001
	12 months post-randomisation	5 (2 – 10)	35.8			
**HADS-D** Depression (*n* 498)	Baseline	7 (4 – 10)	46.4	-22.3	-27.0 to -17.6	<0.001
	12 months post-randomisation	3 (1 – 7)	24.1			

Characteristics of 12-month post-randomisation HADS questionnaire returners and non-returners

The prevalence of baseline anxiety and depression was similar among those who did and did not return a completed questionnaire. Baseline BMI, participant sex, ethnicity, marital status, smoking status, and self-reported income were not associated with repeat HADS questionnaire return [***Supplementary Table 2, Supplementary Table 3, Supplementary Table 4***].

Factors associated with 12-month post-randomisation HADS return included participant age and employment status. Participants who returned completed anxiety or depression questionnaires were on average older (HADS-A: 4.1 years older, 95% CI 2.5 to 5.8, $p < 0.001$; HADS-D: 4.3 years older, 95% CI 2.7 to 6.0, $p < 0.001$) than participants who did not return completed questionnaires. Compared to individuals who were in employment, being retired at baseline was associated with an increased

For peer review only - http://bmjopen.bmj.com/site/about/guidelines.xhtml

odds of completed HADS-A (Odds Ratio (OR) 2.7, 95% CI 1.2 to 5.8, $p < 0.01$) and HADS-D (OR 2.4, 95% CI 1.1 to 5.0, $p < 0.05$) questionnaire return at 12 months post-randomisation.

DISCUSSION

In this study of the course of common mental health disorders in a population of randomised participants undergoing bariatric surgery, nearly half of the sample met criteria for possible or probable anxiety or depression on trial enrolment. Following surgery, substantial reductions in the proportion of participants with possible depression and anxiety were observed at 12 months post-randomisation. The greatest reduction was observed in symptoms of depression, where there was over a 20% decrease in prevalence. Whilst most participants reported an improvement in their mental health, over a third retained symptoms of an underlying anxiety disorder and a quarter of participants met criteria for a depressive disorder at 12 months post-randomisation.

Compared to previously published research utilising the HADS, we found higher a prevalence of pre-operative anxiety and depression in our study sample. Karlsson et al. described HADS scores amongst a consecutive sample of participants (*n* 655) who took part in the Swedish Obese Subjects (SOS) study and underwent bariatric surgery [29]. Using identical cut-off points to those used in our study, the prevalence of pre-operative anxiety was 34% and that of depression was 24% among those who were surgically treated. Whilst the mean age of their sample was comparable to ours, the mean BMI (41.9 SD 4.2 kg/m^2) was lower. The increased BMI among our sample may reflect the higher rate of adult obesity within the UK population,

For peer review only - http://bmjopen.bmj.com/site/about/guidelines.xhtml

alongside the substantially lower number of bariatric surgical procedures taking place in the UK compared to Sweden and other European countries [30]. This may also be linked to the higher levels of depression and anxiety in our sample. In a prospective study of people who underwent bariatric surgery (*n* 153) recruited from six surgical centres in Austria and Germany, Burgmer et al. found that 40.5% of the sample had depression (HADS-D $\geq$8) at baseline which decreased to 17.1% after one year following surgery [31]. Participants had a higher mean BMI (51.3 SD 8.4 kg/m^2) compared to those enrolled in this study. However, they did not find any significant changes in anxiety caseness which could have arisen due to the use of a higher (HADS-A $\geq$10) case cut-off score.

The significant reduction in depression prevalence and symptom severity observed over the first post-operative is in keeping with other studies which have utilised differing assessment criteria, such as the Beck Depression Inventory (BDI) or structured clinical interview [19]. In the Longitudinal Assessment of Bariatric Surgery series (LABS), a large multicentre cohort study of adults undergoing bariatric surgery in the USA, the authors found that LABS-2 surgery recipients (*N* 2,148) monitored over three years experienced the greatest reduction in mean BDI score between baseline and one-year post-operatively [32]. In their study, participants with pre-operative depressive symptoms (defined using a BDI score of $\geq$10) were significantly more likely, than those with minimal or no symptoms, to experience depressive symptoms on follow-up. Whilst the literature has predominantly studied the trajectory of depressive disorders, structured clinical interviews could offer greater insight into the pre-operative prevalence of anxiety disorders. In a sub-sample of LABS participants (*N* 199) interviewed before bariatric surgery, 18.1% (*n* 36) were found to

For peer review only - http://bmjopen.bmj.com/site/about/guidelines.xhtml

have a current anxiety disorder, with a specific phobia (11.1%, *n* 22) being the most common diagnosis [33].

Our study has several strengths. To our knowledge, it is the largest prospective study to assess the short-term effects of bariatric surgery on both anxiety and depressive symptoms in the UK. Participants were screened with a validated case-finding scale which has been shown to be reliable in detecting both disorders [34]. Whereas previous studies have often utilised single dimension instruments. Our large sample were recruited from 12 UK NHS surgical centres that are likely to be representative of the national population undergoing bariatric surgery, compared to those sampled from a single geographical site. We also report the effect size, with respect to change in prevalence of anxiety and depression (an important metric which has been missing from previously published studies in the field [19, 21]), alongside the pre-operative sociodemographic characteristics of questionnaire non-returners which could inform the delivery of future work and targeted interventions for this group. Our finding that repeat questionnaire returners were slightly older (compared to questionnaire non-returners at 12-month post-randomisation) is in keeping with the wider epidemiological literature regarding survey response rates in this age-group [35, 36] and likely linked to the increase in response among those who were retired.

There were also some important limitations. There was a significant questionnaire non-return rate of around 30% at 12 months post-randomisation. Whilst we did not find an association between having poorer mental health at baseline and questionnaire non-return, it is possible that individuals who did not return repeat

For peer review only - http://bmjopen.bmj.com/site/about/guidelines.xhtml

HADS questionnaires may have later developed anxiety or depressive disorders following randomisation. We have also not explored the change in BMI of participants returning or not returning questionnaires in follow up (which may have influenced questionnaire return status) because this primary outcome data remains confidential until analyses of the main trial is completed. The By-Band-Sleeve study remains in active follow-up and it was not possible to compare the differences in symptom scores between surgical groups. As the purpose of this study was to describe the course of anxiety and depressive symptoms, irrespective of procedure type, the research team were not unblinded to participant's surgical intervention status. In terms of sociodemographic characteristics, the participants were predominantly female, identified as being from a White British ethnic background, and in employment at the time of study. It is therefore possible that our findings are not generalisable to the other groups undergoing bariatric surgery, particularly males and individuals from ethnic minorities. It is also plausible that responses to the pre-randomisation HADS questionnaires may have been influenced or affected by social desirability bias, particularly if participants incorrectly perceived that disclosure of their mental health difficulties was going to influence the likelihood of surgery. Participants response to the baseline HADS questionnaires had no bearing on their treatment allocation status and their responses remained confidential.

The role of mental health stigma and marginalisation has been highlighted throughout qualitative research [37, 38] featuring surgery recipients and could contribute to the high prevalence of poor mental health within our sample. Previous work has demonstrated a disparity in gains within mental health-related quality of life (HRQoL) compared to physical HRQoL following bariatric surgery [39] that could

For peer review only - http://bmjopen.bmj.com/site/about/guidelines.xhtml

prove important to understanding the short-term effects within our sample. In a study of LABS-3 participants, the presence of a pre-operative anxiety or affective disorder was associated with reduced improvements in mental HRQoL in the long-term following surgery and was independent of weight gain [40]. Recent research has found that increased physical activity following surgery was associated with a sustained improvement in both mental and physical HRQoL, alongside a reduction in depressive symptoms [41].

CONCLUSIONS

Our study highlights the very high prevalence of pre-operative psychological morbidity amongst people undergoing bariatric surgery for the treatment of severe or complex obesity in the UK. An improvement in symptoms of anxiety and depression was observed following surgery amongst participants who had returned completed questionnaires. Future work must be undertaken to understand the mechanisms underpinning these associations and whether these improvements were sustained in the long-term.

For peer review only - http://bmjopen.bmj.com/site/about/guidelines.xhtml

Contributorship

Dr Jonathan Gibb and all authors within the By-Band-Sleeve Collaborating Group contributed to the drafting and revision of this final manuscript.

Collaborators

<u>The By-Band-Sleeve Collaborating Group</u>

Rob C Andrews PhD, Medical Research, University of Exeter Medical School, Exeter, UK;

John Bessent, Patient representative, By-Band Sleeve Trial management Group, UK;

Jane M Blazeby MD, NIHR Bristol Biomedical Research Centre Bristol Medical School, Population Health Sciences, University of Bristol, Bristol, UK;

James P Byrne MD, University Hospital Southampton NHS Foundation Trust, Southampton, UK;

Nicholas Carter MSc, Portsmouth Hospitals University NHS Trust, Portsmouth, UK;

Caroline Clay (Deceased), Patient representative, By-Band-Sleeve Trial Management Group, UK;

Jenny L Donovan PhD, Bristol Medical School, Population Health Sciences, University of Bristol, Bristol, UK;

Jonathan Gibb MBChB, Centre for Academic Mental Health, Population Health Sciences, Bristol Medical School, University of Bristol, UK;

Eleanor A Gidman PhD, Bristol Trials Centre, Bristol Medical School, University of Bristol, Bristol, UK;

For peer review only - http://bmjopen.bmj.com/site/about/guidelines.xhtml
For peer review only

Graziella Mazza PhD, Bristol Trials Centre, Bristol Medical School, University of Bristol, Bristol, UK;

Paul Moran MD, Centre for Academic Mental Health, Population Health Sciences, Bristol Medical School, University of Bristol, UK;

Mary O'Kane MSc, Dietetic Department, Leeds Teaching Hospitals NHS Trust, Leeds, UK;

Barnaby C Reeves PhD, Bristol Trials Centre, Bristol Medical School, University of Bristol, Bristol, UK;

Chris A Rogers PhD, Bristol Trials Centre, Bristol Medical School, University of Bristol, Bristol, UK;

Nicki Salter DipHE, Somerset NHS Foundation Trust, Somerset, UK;

Janice L Thompson PhD, School of Sport, Exercise & Rehabilitation Sciences, University of Birmingham, Birmingham, UK;

Richard Welbourn MD, Somerset NHS Foundation Trust, Somerset, UK;

Sarah Wordsworth PhD, Health Economics Research Centre, Nuffield Department of Population Health, University of Oxford, UK

For peer review only

Jonathan Gibb developed the study idea based on existing trial data (conceived and developed by the By-Band-Sleeve Trial Management Group), contributed to the statistical analysis plan, undertook data-analysis, wrote the first draft, and revised the final manuscript. Jane M Blazeby (By-Band-Sleeve Chief Investigator) developed the sub-study idea, contributed to design, contributed to the statistical analysis plan, and co-supervised the project; Paul Moran developed the sub-study idea, contributed to design, contributed to the statistical analysis plan, and co-supervised the project. Graziella Mazza contributed to the study design; Eleanor A Gidman prepared the

For peer review only - http://bmjopen.bmj.com/site/about/guidelines.xhtml

sub-study dataset and contributed to the statistical analysis plan; Chris A Rogers contributed to the statistical analysis plan.

Acknowledgements

Independent data monitoring committee

Craig Ramsay (Chair), Health Services Research Unit, University of Aberdeen UK

Nick Finer, UCLH centre for weight loss, metabolic and endocrine surgery, London, UK

Torsten Olbers, Sahlgrenska University Hospital, Sweden

Trial Steering Committee

Julia Brown (Chair), Leeds Institute of Clinical Trials Research, Leeds UK

John Dixon, Baker IDI Heart and Diabetes Institute, Melbourne, Australia

Steve Morris, Department of Public Health and Primary Care, University of Cambridge

Jodie Smith, Patient representative

Michel Suter, Université de Lausanne, Switzerland

John Wilding, Clinical Sciences Centre, University Hospital Aintree, Liverpool, UK

Lead research nurses

Sally Abbott, Benita Adams, Alison Fletcher, Hassina Furreed, Hussain Gordon, Jennifer Henderson, Helen Horton, Tracey Lee, Amy Long, Melody MacGregor, Sarah Matthias, Maria Moon, Catherine Moriarty, Rosemary Mullett, Nicki Salter, Jill Townley

For peer review only - http://bmjopen.bmj.com/site/about/guidelines.xhtml

Research nurses and practitioners

Philippa Allison, Fiona Brogan, Katie Cook, Paul Corrigan, , Anne Daw, Naomi Dindol, Jacqueline Dingle, Eve Fletcher, Jeremy Gilbert, Ana Gill, Beth Greenslade, Andrew Guy, Madeleine Hawkes, Emma Holzer, Lianne Hufton, Lucy Johnstone, Jasmine Jose, Susan Kelly, Krishna Kholia, Jasmina Mandair, Claire Mason, Priya Mathew, Maxine Nixon, Madeleine Pappas, Mark Priestley, Tracey Robson, Jana Rojkova, Rachel Schranz, Barbara Watkins, Louise White

Bariatric surgeons

Ahmed Ahmed, Sanjay Agrawal, Sara Ajaz, Waleed Al-Khyatt, Sherif Awad, Altaf Awan, Shlok Balupuri, Ashok Bohra, James Byrne, Richard Byrom, Nicholas Carter, Michael Clarke, Allwyn Cota, Markos Daskalakis, Nick Davies, Simon Dexter, Ian Finlay, Jeremy Hayden, James Hopkins, Noah Howes, Khaleel Fareed, Sherif Hakky, James Hewes, Neil Jennings, Jamie Kelly, Ben Knight, Yashwant Koak, Moorthy Krishna, Paul Leeder, John Loy, Brijesh Madhok, Kamal Mahawar, David Mahon, Matthew Mason, Samir Mehta, Rajwinder Nijjar, Hamish Noble, Alan Osborne, Dimitri Pournaras, Sanjay Purkayastha, Martin Richardson, Abeezar Sarela, Rishi Singhal, Peter Small, Shaw Somers, Paul Super, Christos Tsironis, Richard Welbourn

For peer review only - http://bmjopen.bmj.com/site/about/guidelines.xhtml

Declaration of interests

From the By-Band-Sleeve Collaborative Group:

James P Byrne MD, On the By-Band-Sleeve Trial Management Group, is on the medical advisory board for the company Oxford Medical Products. All other authors declare no competing interests.

From those acknowledged:

Sanjay Agrawal received a royalty from Springer Publishers for being Editor of the book – 'Obesity, Bariatric and Metabolic Surgery-A Practical Guide' in addition to honoraria for lectures given at national and international bariatric meetings; Sanjay Agrawal is also the Director of Bariatric and Metabolic Surgery UK: Not for Profit – Charity Company; Company No: 11729612, Registered in England & Wales. Sherif Awad receives honoraria for lectures delivered at bariatric meetings. Nick Finer is the Chair of the Trial Steering Committee for the iPREVENT study (NIHR funded EME Project:15/185/16 - Increase colonic propionate as a method of preventing weight gain in young adults). John Dixon previously served as a consultant for the company Reshape who own the LapBand.

Ethical approval

The By-band study gained National Health Service (NHS) ethics approval from the South West Frenchay Research Ethics Committee (REC No: 11/SW/0248) on the 6th December 2011 and on the 8th May 2015 the Ethics Committee granted ethical approval to adapt the study from a two group (By-Band) to a three group (By-Band-Sleeve) trial. REC approval applies to all NHS sites taking part in the study. The

For peer review only - http://bmjopen.bmj.com/site/about/guidelines.xhtml

study is sponsored by the University of Bristol and it is the responsibility of the sponsor to ensure that all the conditions of the study are complied with. In addition, By-Band-Sleeve study was processed under pre-Health Research Authority (HRA) Approval systems, the study was granted HRA approval on the 24th July 2017. The By-Band-Sleeve Study is registered with the National Institutes of Health ClinicalTrials.gov database (NCT02841527) and ISRCTN registry (ISRCTN00786323).

Funding statement

The By-Band-Sleeve study is funded by the United Kingdom National Institute for Health Research (NIHR) HTA programme (ref: 09/127/53). The views and opinions expressed are those of the authors and do not necessarily reflect those of the HTA programme, the NIHR, the UK NHS, or the Department of Health. The trial is being delivered in collaboration with Bristol Trials Centre, a UKCRC registered clinical trials unit which is in receipt of NIHR CTU support funding. This study was supported by the NIHR Biomedical Research Centre at University Hospitals Bristol and Weston NHS Foundation Trust and the University of Bristol. Jane M Blazeby MD is partially funded by the Bristol NIHR Biomedical research centre.

Data sharing statement

Data may be obtained from a third party and are not publicly available. Data will not be made available for sharing until after publication of the main results of the randomised trial. Thereafter, anonymised individual patient data will be made available for secondary research, conditional on assurance from the secondary researcher that the proposed use of the data is compliant with the MRC Policy on

For peer review only - http://bmjopen.bmj.com/site/about/guidelines.xhtml

Data Preservation and Sharing regarding scientific quality, ethical requirements, and value for money.

REFERENCES

[1] Dai H, Alsalhe TA, Chalghaf N, Riccò M, Bragazzi NL, Wu J. The global burden of disease attributable to high body mass index in 195 countries and territories, 1990-2017: An analysis of the Global Burden of Disease Study. PLoS Med. 2020;17(7):e1003198.

[2] Vos T, Lim SS, Abbafati C, Abbas KM, Abbasi M, Abbasifard M, et al. Global burden of 369 diseases and injuries in 204 countries and territories, 1990–2019: a systematic analysis for the Global Burden of Disease Study 2019. The Lancet. 2020;396(10258):1204-22.

[3] Santomauro DF, Mantilla Herrera AM, Shadid J, Zheng P, Ashbaugh C, Pigott DM, et al. Global prevalence and burden of depressive and anxiety disorders in 204 countries and territories in 2020 due to the COVID-19 pandemic. The Lancet. 2021;398(10312):1700-12.

[4] Luppino FS, Wit LMd, Bouvy PF, Stijnen T, Cuijpers P, Penninx BWJH, et al. Overweight, Obesity, and Depression: A Systematic Review and Meta-analysis of Longitudinal Studies. Arch. Gen. Psychiatry. 2021;67:220-9.

[5] Pan A, Sun Q, Czernichow S, Kivimaki M, Okereke OI, Lucas M, et al. Bidirectional association between depression and obesity in middle-aged and older women. Int J Obes (Lond). 2012;36(4):595-602.

[6] Jung SJ, Woo H-t, Cho S, Park K, Jeong S, Lee YJ, et al. Association between body size, weight change and depression: systematic review and meta-analysis. British Journal of Psychiatry. 2017;211(1):14-21.

[7] Gariepy G, Nitka D, Schmitz N. The association between obesity and anxiety disorders in the population: a systematic review and meta-analysis. International journal of obesity. 2010 Mar;34(3):407-19.

For peer review only - http://bmjopen.bmj.com/site/about/guidelines.xhtml

[8] de Wit L, Have Mt, Cuijpers P, de Graaf R. Body Mass Index and risk for onset of mood and anxiety disorders in the general population: Results from the Netherlands Mental Health Survey and Incidence Study-2 (NEMESIS-2). BMC Psychiatry. 2022;22(1):522. 2.

[9] F ulton S, Décarie-Spain L, Fioramonti X, Guiard B, Nakajima S. The menace of obesity to depression and anxiety prevalence. Trends in Endocrinology & Metabolism. 2022;33(1):18-35.

[10] McLaughlin AP, Nikkheslat N, Hastings C, Nettis MA, Kose M, Worrell C, et al. The influence of comorbid depression and overweight status on peripheral inflammation and cortisol levels. Psychological Medicine. 2022;52(14):3289-96.

[11] Borgland SL. Can treatment of obesity reduce depression or vice versa? J Psychiatry Neurosci. 2021;46(2):E313-e8.

[12] National Institute for Health and Care Excellence. Obesity: identification, assessment and management [Internet]. [London]: NICE; 2014 [updated 2022 Sept 08; cited 2022 Dec 04]. (Clinical guideline [CG189]). Available from: https://www.nice.org.uk/guidance/cg189

[13] Douglas IJ, Bhaskaran K, Batterham RL, Smeeth L. Bariatric Surgery in the United Kingdom: A Cohort Study of Weight Loss and Clinical Outcomes in Routine Clinical Care. PLoS Med. 2015;12(12):e1001925.

[14] Syn NL, Cummings DE, Wang LZ, Lin DJ, Zhao JJ, Loh M, et al. Association of metabolic-bariatric surgery with long-term survival in adults with and without diabetes: a one-stage meta-analysis of matched cohort and prospective controlled studies with 174;772 participants. Lancet. 2021;397(10287):1830-41.

[15] Gloy VL, Briel M, Bhatt DL, Kashyap SR, Schauer PR, Mingrone G, et al. Bariatric surgery versus non-surgical treatment for obesity: a systematic review and meta-analysis of randomised controlled trials. BMJ. 2013;347:f5934.

[16] Nguyen NT, Varela JE. Bariatric surgery for obesity and metabolic disorders: state of the art. Nat. Rev. Gastroenterol. Hepatol. 2016;14:160-9.

For peer review only - http://bmjopen.bmj.com/site/about/guidelines.xhtml

[17] National Bariatric Surgery Registry. The United Kingdom National Bariatric Surgery Registry 3rd Report. 2020. Available from: https://e-dendrite.com/Publishing/Reports/Bariatric/NBSR2020.pdf [Accessed 12 Apr 2023]

[18] Booth H, Khan O, Prevost AT, Reddy M, Charlton J, Gulliford MC. Impact of bariatric surgery on clinical depression. Interrupted time series study with matched controls. J. Affect. Disord. 2015;174:644-9.

[19] Dawes AJ, Maggard-Gibbons M, Maher AR, Booth MJ, Miake-Lye I, Beroes JM, et al. Mental Health Conditions Among Patients Seeking and Undergoing Bariatric Surgery: A Meta-analysis. JAMA. 2016;315:150-63.

[20] Fu R, Zhang Y, Yu K, Mao D, Su H. Bariatric surgery alleviates depression in obese patients: A systematic review and meta-analysis. Obesity Research & Clinical Practice. 2022;16(1):10-6.

[21] Gill H, Kang S, Lee Y, Rosenblat JD, Brietzke E, Zuckerman H, et al. The long-term effect of bariatric surgery on depression and anxiety. J. Affect. Disord. 2019;246:886-94.

[22] de Zwaan M, Enderle J, Wagner S, Mühlhans B, Ditzen B, Gefeller O, et al. Anxiety and depression in bariatric surgery patients: A prospective, follow-up study using structured clinical interviews. Journal of Affective Disorders. 2011;133(1):61-8.

[23] Neovius M, Bruze G, Jacobson P, Sjöholm K, Johansson K, Granath F, et al. Risk of suicide and non-fatal self-harm after bariatric surgery: results from two matched cohort studies. Lancet Diabetes Endocrinol. 2018;6:197-207.

[24] Backman O, Stockeld D, Rasmussen F, Näslund E, Marsk R. Alcohol and substance abuse, depression and suicide attempts after Roux-en-Y gastric bypass surgery. Br J Surg. 2016;103:1336-42.

[25] Rogers CA, Reeves BC, Byrne J, Donovan JL, Mazza G, Paramasivan S, et al. Adaptation of the By-Band randomised clinical trial to By-Band-Sleeve to include a new intervention and maintain relevance of the study to practice. Br J Surg. 2017;104:1207-14.

For peer review only - http://bmjopen.bmj.com/site/about/guidelines.xhtml

[26] Rogers CA, Welbourn R, Byrne J, Donovan JL, Reeves BC, Wordsworth S, et al. The By-Band study: gastric bypass or adjustable gastric band surgery to treat morbid obesity: study protocol for a multi-centre randomised controlled trial with an internal pilot phase. Trials. 2014;15:53.

[27] Zigmond AS, Snaith RP. The hospital anxiety and depression scale. Acta Psychiatr Scand. 1983;67:361-70.

[28] Bjelland I, Dahl AA, Haug TT, Neckelmann D. The validity of the Hospital Anxiety and Depression Scale: An updated literature review. J. Psychosom. Res. 2002;52:69-77.

[29] Karlsson J, Taft C, Rydén A, Sjöström L, Sullivan M. Ten-year trends in health-related quality of life after surgical and conventional treatment for severe obesity: the SOS intervention study. Int J Obes (Lond). 2007;31:1248-61.

[30] Borisenko O, Colpan Z, Dillemans B, Funch-Jensen P, Hedenbro J, Ahmed AR. Clinical Indications, Utilization, and Funding of Bariatric Surgery in Europe. Obes Surg. 2015;25:1408-16.

[31] Burgmer R, Petersen I, Burgmer M, de Zwaan M, Wolf AM, Herpertz S. Psychological outcome two years after restrictive bariatric surgery. Obes Surg. 2007;17(6):785-91.

[32] Mitchell JE, King WC, Chen J-Y, Devlin MJ, Flum D, Garcia L, et al. Course of depressive symptoms and treatment in the longitudinal assessment of bariatric surgery (LABS-2) study. Obesity. 2014;22(8):1799-806.

[33] Mitchell JE, Selzer F, Kalarchian MA, Devlin MJ, Strain GW, Elder KA, et al. Psychopathology before surgery in the longitudinal assessment of bariatric surgery-3 (LABS-3) psychosocial study. Surg Obes Relat Dis. 2012;8(5):533-41.

[34] Hansson M, Chotai J, Nordstöm A, Bodlund O. Comparison of two self-rating scales to detect depression: HADS and PHQ-9. Br J Gen Pract. 2009;59(566):e283-8.

For peer review only - http://bmjopen.bmj.com/site/about/guidelines.xhtml

[35]. Robb KA, Gatting L, Wardle J. What impact do questionnaire length and monetary incentives have on mailed health psychology survey response? British Journal of Health Psychology. 2017;22(4):671-85.

[36]. Regine A, Martin Veel S, Paul KH, Gølin Finckenhagen G, Kjell T, Johny K, et al. Non-response in a cross-sectional study of respiratory health in Norway. BMJ Open. 2016;6(1):e009912.

[37] Homer CV, Tod AM, Thompson AR, Allmark P, Goyder E. Expectations and patients' experiences of obesity prior to bariatric surgery: a qualitative study. BMJ Open. 2016;6:e009389.

[38] Raves DM, Brewis A, Trainer S, Han S-Y, Wutich A. Bariatric Surgery Patients' Perceptions of Weight-Related Stigma in Healthcare Settings Impair Post-surgery Dietary Adherence. Front Psychol. 2016;7:1497-.

[39] Lindekilde N, Gladstone BP, Lübeck M, Nielsen J, Clausen L, Vach W, et al. The impact of bariatric surgery on quality of life: a systematic review and meta-analysis. Obes Rev. 2015;16:639-51.

[40] Kalarchian MA, King WC, Devlin MJ, Hinerman A, Marcus MD, Yanovski SZ, et al. Mental disorders and weight change in a prospective study of bariatric surgery patients: 7 years of follow-up. Surg Obes Relat Dis. 2019;15(5):739-48.

[41] King WC, Hinerman AS, White GE, Courcoulas AP, Belle SH. Associations Between Physical Activity and Changes in Depressive Symptoms and Health-related Quality of Life Across 7 Years After Roux-en-Y Gastric Bypass Surgery: A Multicenter Prospective Cohort Study. Ann Surg. 2022;276(6):e777-e83.

For peer review only - http://bmjopen.bmj.com/site/about/guidelines.xhtml

LEGENDS

TABLE 1: Demographic data by baseline HADS-A and HADS-D scores. Case categories based on the original HADS cut-offs proposed by Zigmond & Snaith, 1983.

TABLE 2: Frequency and percentage change in clinical cases for individuals who underwent surgery within 6 months of randomisation with completed questionnaires at both timepoints. p-value associated with change in case proportions obtained from McNemar's chi-squared test statistic.

FIGURE 1: Flow Diagram

Flow diagram representing questionnaire subscale completion for the HADS amongst the sample obtained from the By-Band-Sleeve study.

FIGURE 2: HADS Anxiety (HADS-A) and Depression (HADS-D)

Total symptom scores for participants who completed HADS-A or HADS-D subscales at baseline (pre-randomisation) and 12 months post-randomisation. The horizontal black line at the HADS Score of 8 on the y axis represents the cut-off for clinical cases. For both anxiety and depression, there was a significant ($p < 0.001$) decrease in median HADS score at 12 months post-randomisation**.**

FIGURE 3: Change in clinical cases

Proportion of possible clinical cases (HADS-A/D $\geq$8) at baseline and 12 months post-randomisation. Each bar represents the case prevalence (with associated 95% confidence interval) for anxiety and depression. At 12 months post-randomisation, there was a reduction in the proportion of individuals with anxiety (9.5% decrease, 95% CI -14.3 to -4.8%, $p < 0.001$) and depression (22.3% decrease, 95% CI -27.0 to -17.6%, $p < 0.001$).

For peer review only - http://bmjopen.bmj.com/site/about/guidelines.xhtml

Figure 1: Participant flow

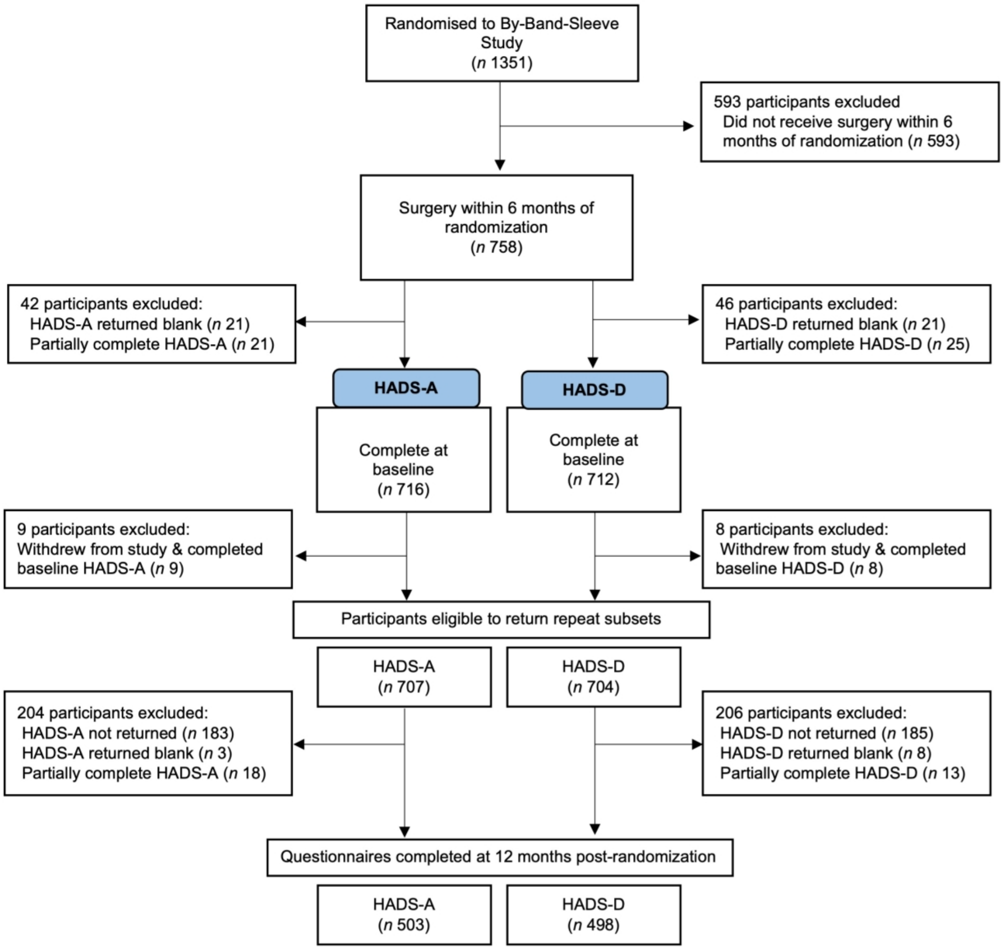

Flow diagram representing questionnaire subscale completion for the HADS amongst the sample obtained from the By-Band-Sleeve study.

192x192mm (330 x 330 DPI)

Figure 2: HADS Anxiety (HADS-A) and Depression (HADS-D)

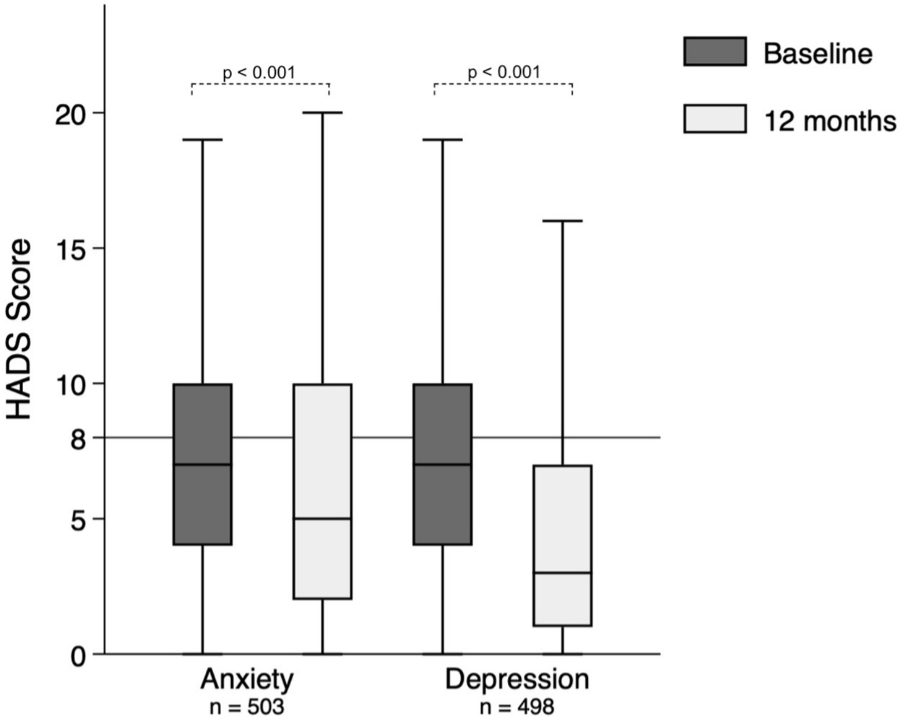

Total symptom scores for participants who completed HADS-A or HADS-D subscales at baseline (pre-randomisation) and 12 months post-randomisation. The horizontal black line at the HADS Score of 8 on the y axis represents the cut-off for clinical cases. For both anxiety and depression, there was a significant ($p < 0.001$) decrease in median HADS score at 12 months post-randomisation.

159x136mm (220 x 220 DPI)

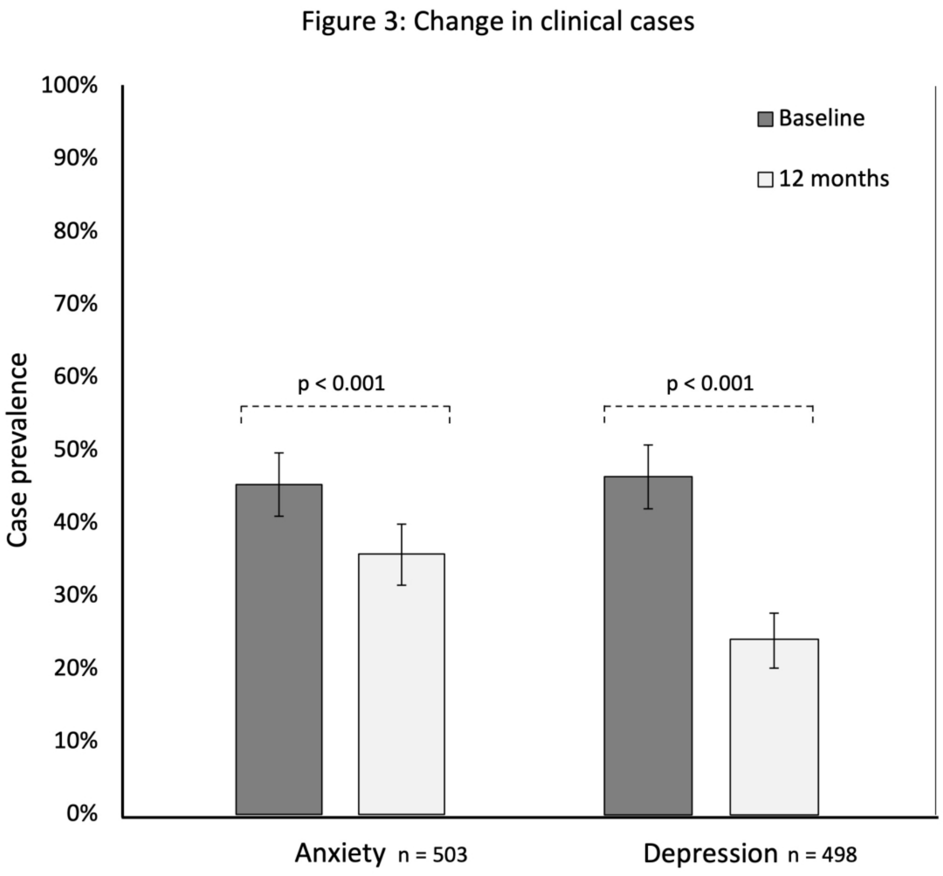

Proportion of possible clinical cases (HADS-A/D >8) at baseline and 12 months post-randomisation. Each bar represents the case prevalence (with associated 95% confidence interval) for anxiety and depression. At 12 months post-randomisation, there was a reduction in the proportion of individuals with anxiety (9.5% decrease, 95% CI -14.3 to -4.8%, $p < 0.001$) and depression (22.3% decrease, 95% CI -27.0 to -17.6%, $p < 0.001$).

165x149mm (330 x 330 DPI)

Supplementary Results

Anxiety and depression following bariatric surgery

List of supplementary tables

Page

Table 1	Change in clinical cases from baseline to 12 months post-randomisation	2
Table 2	Baseline characteristics by repeat HADS questionnaire return status	3
Table 3	Baseline characteristics by repeat HADS-A questionnaire return status	4
Table 4	Baseline characteristics by repeat HADS-D questionnaire return status	5

For peer review only

For peer review only - http://bmjopen.bmj.com/site/about/guidelines.xhtml

Supplementary Results

Anxiety and depression following bariatric surgery

Table 1
Change in clinical cases from baseline to 12 months post-randomisation

		**Frequency**	**Proportion** (%)	**95% CI (%)**
**Anxiety** HADS-A (*n* 503)	Non-case unchanged	229	45.5	41.2 – 49.9
	Non-case to case	46	9.2	6.9 – 12.0
	Case to non-case	94	18.7	15.5 – 22.3
	Case unchanged	134	26.6	23.0 – 30.7
**Depression** HADS-D (*n* 498)	Non-case unchanged	245	49.2	44.8 – 53.6
	Non-case to case	22	4.4	2.9 – 6.6
	Case to non-case	133	26.7	23.0 – 30.8
	Case unchanged	98	19.7	16.4 – 23.4

For peer review only

For peer review only - http://bmjopen.bmj.com/site/about/guidelines.xhtml

Supplementary Results

Anxiety and depression following bariatric surgery

Table 2
Participant baseline characteristics by repeat HADS questionnaire return status (Continuous variables)

		12 months post-randomisation HADS-A return			
		**Yes** (N = 503)	**No** (N = 204)	**Difference** (95% CI)	**p-value**[1]
**Age** (years)	Mean (95% CI)	48.7 (47.8 to 49.6)	44.6 (43.1 to 46.0)	4.1 (2.5 to 5.8)	<0.001
**BMI** (kg/m2)	Mean (95% CI)	46.3 (45.7 to 46.8)	46.8 (45.8 to 47.7)	-0.5 (-1.6 to 0.6)	0.379
**Time from randomisation to surgery** (days)	Mean (95% CI)	92.6 (88.7 to 96.5)	91.5 (85.5 to 97.6)	1.1 (-6.1 to 8.3)	0.767
		**12 months post-randomisation HADS-D return**			
		**Yes** (N = 498)	**No** (N = 206)	**Difference** (95% CI)	**p-value**[1]
**Age** (years)	Mean (95% CI)	48.8 (47.9 to 49.7)	44.5 (43.1 to 45.9)	4.3 (2.7 to 6.0)	<0.001
**BMI** (kg/m2)	Mean (95% CI)	46.3 (45.7 to 46.9)	46.8 (45.8 to 47.7)	-0.5 (-1.6 to 0.6)	0.370
**Time from randomisation to surgery** (days)	Mean (95% CI)	92.9 (89.0 to 96.8)	91.8 (85.8 to 97.8)	1.1 (-6.1 to 8.3)	0.767

[1] p-value obtained from paired sample t-test statistic for mean difference by HADS return status

For peer review only - http://bmjopen.bmj.com/site/about/guidelines.xhtml

Supplementary Results

Anxiety and depression following bariatric surgery

Table 3
Participant characteristics by repeat HADS-A questionnaire return status (categorical variables)

		12 months post-randomisation HADS-A return			
		**Yes** (%) N = 503 (71.15)	**No** (%) N = 204 (28.85)	**Odds Ratio for non-return**[2] (95% CI)	**p-value**[3]
**Sex**	Male (*n* 178)	126 (70.79)	52 (29.21)	1.00	0.903
	Female (*n* 529)	377 (71.27)	152 (28.73)	0.98 (0.67 to 1.42)	
**Ethnicity**	White (*n* 658)	472 (71.73)	186 (28.27)	1.00	0.217
	Other ethnic group[4] (*n* 49)	31 (63.27)	18 (36.73)	1.47 (0.80 to 2.70)	
**Marital status**	Married or civil partnership (*n* 395)	280 (70.89)	115 (29.11)	1.00	0.926
	Co-habiting (*n* 84)	62 (73.81)	22 (26.19)	0.86 (0.51 to 1.47)	
	Single (*n* 132)	92 (69.70)	40 (30.30)	1.06 (0.69 to 1.63)	
	Divorced, Separated, or Widowed[5] (*n* 96)	69 (71.88)	27 (28.12)	0.95 (0.58 to 1.56)	
**Smoking status**	Never smoked (*n* 317)	226 (71.29)	91 (28.71)	1.00	0.339
	Ex-smoker (*n* 343)	248 (72.30)	95 (27.70)	0.95 (0.68 to 1.33)	
	Current smoker (*n* 47)	29 (61.70)	18 (38.30)	1.54 (0.82 to 2.91)	
**Employment status**	Employed (*n* 455)	326 (71.65)	129 (28.35)	1.00	0.002
	Not in employment or student[6] (*n* 190)	123 (64.74)	67 (35.26)	1.38 (0.96 to 1.97)	
	Retired (*n* 62)	54 (87.10)	8 (12.90)	0.37 (0.17 to 0.81)	
**Income band**	$\leq$10,000 (*n* 84)	59 (70.24)	25 (29.76)	1.00	0.247
	10,001 to 30,000 (*n* 289)	212 (73.36)	77 (26.64)	0.86 (0.50 to 1.46)	
	30,001 to 50,000 (*n* 150)	105 (70.00)	45 (30.00)	1.01 (0.56 to 1.81)	
	$\geq$50,001 (*n* 91)	69 (75.82)	22 (24.18)	0.75 (0.38 to 1.47)	
	Not disclosed (*n* 92)	57 (61.96)	35 (38.04)	1.45 (0.77 to 2.72)	

2 Odds ratio for questionnaire non-return calculated using logistic regression
3 p-value obtained from likelihood ratio chi-square test
For calculation of Odds ratios associated with questionnaire non-return, categories with sub-groups containing $\leq$5% (*n* 35/707) total respondents were merged with the next largest sub-group to avoid data sparsity: **4** Includes participants who identified as African or Caribbean (*n* 28/707), Mixed ethnic group (*n* 13/707), Asian (*n* 3/707), or Other (*n* 5/707). **5** Participants who identified as separated (*n* 21/703) and widowed (*n* 11/703) combined with those who identified as divorced (*n* 64/703) as next largest category. **6** Participants who identified as students (*n* 6/703) combined with those not in employment (*n* 183/703)

Supplementary Results

Anxiety and depression following bariatric surgery

Table 4
Baseline characteristics by repeat HADS-D questionnaire return status (categorical variables)

		12 months post-randomisation HADS-D return			
		**Yes** (%) N = 498 (70.74)	**No** (%) N = 206 (29.26)	**Odds Ratio for non-return**[2] (95% CI)	**p-value**[3]
**Sex**	Male (*n* 178)	126 (70.79)	52 (29.21)	1.00	0.987
	Female (*n* 526)	372 (70.72)	154 (29.28)	0.98 (0.69 to 1.46)	
**Ethnicity**	White (*n* 655)	468 (71.45)	187 (28.55)	1.00	0.139
	Other ethnic group[4] (*n* 49)	30 (61.22)	19 (38.78)	1.59 (0.87 to 2.89)	
**Marital status**	Married or civil partnership (*n* 390)	273 (70.00)	117 (30.00)	1.00	0.592
	Co-habiting (*n* 84)	64 (76.19)	20 (23.81)	0.73 (0.42 to 1.26)	
	Single (*n* 132)	90 (68.18)	42 (31.82)	1.09 (0.71 to 1.67)	
	Divorced, Separated, or Widowed[5] (*n* 98)	71 (72.45)	27 (27.55)	0.89 (0.54 to 1.45)	
**Smoking status**	Never smoked (*n* 317)	225 (70.98)	92 (29.02)	1.00	0.566
	Ex-smoker (*n* 340)	243 (71.47)	97 (28.53)	0.98 (0.70 to 1.37)	
	Current smoker (*n* 47)	30 (63.83)	17 (36.17)	1.39 (0.73 to 2.63)	
**Employment status**	Employed (*n* 451)	322 (71.40)	129 (28.60)	1.00	0.003
	Not in employment or student[6] (*n* 190)	122 (64.21)	68 (35.79)	1.39 (0.97 to 1.99)	
	Retired (*n* 63)	54 (85.71)	9 (14.29)	0.40 (0.33 to 0.49)	
**Income band**	≤10,000 (*n* 86)	59 (68.60)	27 (31.40)	1.00	0.108
	10,001 to 30,000 (*n* 286)	207 (72.38)	79 (27.62)	0.83 (0.49 to 1.41)	
	30,001 to 50,000 (*n* 149)	103 (69.13)	46 (30.87)	0.98 (0.55 to 1.73)	
	≥50,001 (*n* 91)	72 (79.12)	19 (20.88)	0.58 (0.29 to 1.14)	
	Not disclosed (*n* 91)	56 (61.54)	35 (38.46)	1.37 (0.73 to 2.54)	

[2] Odds ratio for questionnaire non-return obtained using logistic regression
[3] P-value obtained from likelihood ratio chi-square test
For calculation of Odds ratios associated with questionnaire non-return, categories with sub-groups containing ≤5% (*n* 35/707) total respondents were merged into the next largest sub-group to avoid data sparsity: [4] Includes participants who identified as African or Caribbean (*n* 28/704), Mixed ethnic group (*n* 13/707), Asian (*n* 3/707), or Other (*n* 5/707). [5] Participants who identified as separated (*n* 21/703) and widowed (*n* 11/703) combined with those who identified as divorced (*n* 66/703) as next largest category. [6] Participants who identified as students (*n* 7/703) combined with those not in employment (*n* 183/703).

For peer review only - http://bmjopen.bmj.com/site/about/guidelines.xhtml

STROBE: Anxiety and depression following bariatric surgery

STROBE Statement

	Item No	Recommendation	Page No
**Title and abstract**	1	(*a*) Indicate the study's design with a commonly used term in the title or the abstract	1
		(*b*) Provide in the abstract an informative and balanced summary of what was done and what was found	4, 5
**Introduction**			
Background/rationale	2	Explain the scientific background and rationale for the investigation being reported	7, 8
Objectives	3	State specific objectives, including any prespecified hypotheses	4, 5, 8, 9
**Methods**			
Study design	4	Present key elements of study design early in the paper	8, 10
Setting	5	Describe the setting, locations, and relevant dates, including periods of recruitment, exposure, follow-up, and data collection	10, 11, 12, 13
Participants	6	(*a*) Give the eligibility criteria, and the sources and methods of selection of participants	10
Variables	7	Clearly define all outcomes, exposures, predictors, potential confounders, and effect modifiers. Give diagnostic criteria, if applicable	10, 11
Data sources/ measurement	8*	For each variable of interest, give sources of data and details of methods of assessment (measurement). Describe comparability of assessment methods if there is more than one group	10, 11, 12
Bias	9	Describe any efforts to address potential sources of bias	10, 11
Study size	10	Explain how the study size was arrived at	10
Quantitative variables	11	Explain how quantitative variables were handled in the analyses. If applicable, describe which groupings were chosen and why	11, 12
Statistical methods	12	(*a*) Describe all statistical methods, including those used to control for confounding	11, 12
		(*b*) Describe any methods used to examine subgroups and interactions	11, 12
		(*c*) Explain how missing data were addressed	11, 12
		(*d*) If applicable, describe analytical methods taking account of sampling strategy	11, 12
		(*e*) Describe any sensitivity analyses	11, 12
**Results**			
Participants	13*	(a) Report numbers of individuals at each stage of study—eg numbers potentially eligible, examined for eligibility, confirmed eligible, included in the study, completing follow-up, and analysed	14, 15, Figure 1 [Flow diagram]
		(b) Give reasons for non-participation at each stage	14, 15, Figure 1
		(c) Consider use of a flow diagram	Figure 1

For peer review only

STROBE: Anxiety and depression following bariatric surgery

Descriptive data	14*	(a) Give characteristics of study participants (eg demographic, clinical, social) and information on exposures and potential confounders	15<br>Table 1 (All)<br>Appendix A (Non-responders)
		(b) Indicate number of participants with missing data for each variable of interest	15, 18, Table 1, Figure 1
Outcome data	15*	Report numbers of outcome events or summary measures	14, 15, 16, 17, 18<br>Table 2, Table 3, Figure 2, Figure 3, Appendix A
Main results	16	(*a*) Give unadjusted estimates and, if applicable, confounder-adjusted estimates and their precision (eg, 95% confidence interval). Make clear which confounders were adjusted for and why they were included	14, 17, Table 2
		(*b*) Report category boundaries when continuous variables were categorized	15, Table 1, Table 2, Table 3
		(*c*) If relevant, consider translating estimates of relative risk into absolute risk for a meaningful time period	N/A
Other analyses	17	Report other analyses done—eg analyses of subgroups and interactions, and sensitivity analyses	18, Appendix A
**Discussion**			
Key results	18	Summarise key results with reference to study objectives	19
Limitations	19	Discuss limitations of the study, taking into account sources of potential bias or imprecision. Discuss both direction and magnitude of any potential bias	20, 21
Interpretation	20	Give a cautious overall interpretation of results considering objectives, limitations, multiplicity of analyses, results from similar studies, and other relevant evidence	19, 20
Generalisability	21	Discuss the generalisability (external validity) of the study results	20
**Other information**			
Funding	22	Give the source of funding and the role of the funders for the present study and, if applicable, for the original study on which the present article is based	28

*Give information separately for exposed and unexposed groups.

Note: An Explanation and Elaboration article discusses each checklist item and gives methodological background and published examples of transparent reporting. The STROBE checklist is best used in conjunction with this article (freely available on the Web sites of PLoS Medicine at http://www.plosmedicine.org/, Annals of Internal Medicine at http://www.annals.org/, and Epidemiology at http://www.epidem.com/). Information on the STROBE Initiative is available at www.strobe-statement.org.

For peer review only

BMJ Open

Prevalence and short-term change in symptoms of anxiety and depression following bariatric surgery: a prospective cohort study

Journal:	*BMJ Open*
Manuscript ID	bmjopen-2022-071231.R2
Article Type:	Original research
Date Submitted by the Author:	22-Nov-2023
Complete List of Authors:	Band-Sleeve Trial Management Group, (Group Authorship); By-Band-Sleeve Collaborating group<br>Gibb, Jonathan; University of Bristol,
**Primary Subject Heading**:	Mental health
Secondary Subject Heading:	Surgery, Nutrition and metabolism, Epidemiology
Keywords:	Depression & mood disorders < PSYCHIATRY, Anxiety disorders < PSYCHIATRY, Adult surgery < SURGERY, EPIDEMIOLOGIC STUDIES

SCHOLARONE™
Manuscripts

I, the Submitting Author has the right to grant and does grant on behalf of all authors of the Work (as defined in the below author licence), an exclusive licence and/or a non-exclusive licence for contributions from authors who are: i) UK Crown employees; ii) where BMJ has agreed a CC-BY licence shall apply, and/or iii) in accordance with the terms applicable for US Federal Government officers or employees acting as part of their official duties; on a worldwide, perpetual, irrevocable, royalty-free basis to BMJ Publishing Group Ltd ("BMJ") its licensees and where the relevant Journal is co-owned by BMJ to the co-owners of the Journal, to publish the Work in this journal and any other BMJ products and to exploit all rights, as set out in our licence.

The Submitting Author accepts and understands that any supply made under these terms is made by BMJ to the Submitting Author unless you are acting as an employee on behalf of your employer or a postgraduate student of an affiliated institution which is paying any applicable article publishing charge ("APC") for Open Access articles. Where the Submitting Author wishes to make the Work available on an Open Access basis (and intends to pay the relevant APC), the terms of reuse of such Open Access shall be governed by a Creative Commons licence – details of these licences and which Creative Commons licence will apply to this Work are set out in our licence referred to above.

Other than as permitted in any relevant BMJ Author's Self Archiving Policies, I confirm this Work has not been accepted for publication elsewhere, is not being considered for publication elsewhere and does not duplicate material already published. I confirm all authors consent to publication of this Work and authorise the granting of this licence.

For peer review only

Prevalence and short-term change in symptoms of anxiety and depression following bariatric surgery: a prospective cohort study

On behalf of The By-Band-Sleeve Collaborating Group

Corresponding author

Dr Jonathan Gibb

Centre for Academic Mental Health, Population Health Sciences

Bristol Medical School, University of Bristol

Oakfield Grove, Clifton, Bristol BS8 2BN

Jonathan.gibb@bristol.ac.uk

+44 (0)117 428 2489

Word Count (Excluding title page, abstract, references, figures and table): **3,298**

For peer review only - http://bmjopen.bmj.com/site/about/guidelines.xhtml

ABSTRACT

Objectives: Bariatric surgery is an effective treatment for severe obesity that leads to significant physical health improvements. Few studies have prospectively described the short-term impact of surgery on mental health using standardised case-finding measures for anxiety or depressive disorders. This study describes the prevalence and short-term course of these conditions following surgery.

Design: Prospective observational cohort study.

Setting: 12 National Health Service centres in England.

Participants: Participants studied took part in the By-Band-Sleeve study, a multi-centre randomised controlled trial evaluating the surgical management of severe obesity. We included participants who had undergone surgery (Gastric Bypass, Gastric Band or Sleeve Gastrectomy) within 6 months of randomisation.

Primary and secondary outcome measures: Anxiety and depression were assessed using the Hospital Anxiety and Depression Scale (HADS) at baseline and 12 months post-randomisation. Sociodemographic variables collected at pre-randomisation included Body Mass Index, Age, Sex, Ethnicity, Marital Status, Tobacco use, Employment Status, and Income Band.

Results: In our sample of 758 participants, 94.5% (n 716) and 93.9% (n 712) had completed baseline anxiety (HADS-A) and depression (HADS-D) subscales. At pre-randomisation 46.1% (n 330/716, 95% CI 42.4 to 49.7%) met clinical case criteria for

For peer review only - http://bmjopen.bmj.com/site/about/guidelines.xhtml

anxiety and 48.2% (n 343/712, 95% CI 44.5 to 51.8%) for depression. Among participants returning completed 12 months post-randomisation questionnaires (HADS-A n 503/716, HADS-D n 498/712), there was a significant reduction in the proportion of clinical cases with anxiety (-9.5%, 95% CI -14.3 to -4.8% $p < 0.001$) and depression (-22.3%, 95% CI -27.0 to -17.6% $p < 0.001$).

Conclusions: Almost half of people undergoing bariatric surgery had underlying anxiety or depressive symptoms. In the short term, these symptoms appear to substantially improve. Future work must identify whether these effects are sustained beyond the first post-randomisation year.

Trial registration: The By-Band-Sleeve Study is registered with ClinicalTrials.gov database (NCT02841527) and ISRCTN registry (ISRCTN00786323).

For peer review only - http://bmjopen.bmj.com/site/about/guidelines.xhtml

Strengths and limitations of this study

- A validated self-report measure, the Hospital Anxiety and Depression Scale (HADS), was used to detect anxiety and depressive disorders.

- Participants were recruited from the largest randomised controlled trial, to date, in bariatric surgery (The By-Band-Sleeve Study) from multiple NHS surgical centres in England.

- Although participants were re-assessed using the HADS at one-year post-randomisation, the total follow-up period from surgery was relatively short. It is possible that these changes were not maintained after the first post-operative year.

- With respect to surgical procedure, participants were analysed as a whole group, rather than being stratified by surgery type (Gastric Bypass, Gastric Band or Sleeve Gastrectomy).

For peer review only - http://bmjopen.bmj.com/site/about/guidelines.xhtml

INTRODUCTION

Obesity and common mental disorders, such as anxiety and depression, contribute greatly to global disease burden and pose significant public health challenges [1, 2, 3]. There has been a recent focus on understanding the relationship between obesity and common mental disorders. Systematic reviews and meta-analyses of longitudinal studies have found a bi-directional relationship between having obesity and developing a depressive disorder [4, 5] across both sexes, however a recently updated review found an elevated risk only among females [6]. Whilst there have been fewer longitudinal studies assessing the relationship between obesity and anxiety disorders, there is evidence of a positive association between the two conditions [7, 8]. These findings have coincided with a growing body of research studying the potential shared neurobiological (the role of prolonged inflammatory changes, cortisol dysregulation, metabolic dysfunction, and disrupted cellular signalling) pathways between obesity, anxiety states, and depression which may eventually give rise to a better understanding of these common co-morbidities [9, 10, 11].

When people with severe or complex obesity (Body Mass Index $\geq$40kg/m^2 or $\geq$35kg/m^2 with a significant co-morbidity) are unable to lose weight, and have attempted all relevant non-surgical measures, current guidelines in the United Kingdom (UK) recommend that bariatric surgery should be offered [12, 13]. Compared to non-surgical management, bariatric surgery has been shown to be an effective treatment for severe obesity and is associated with gains in overall life expectancy alongside increased remission rates of rates of obesity-related co-morbidities, such as type 2 diabetes mellitus [14, 15, 16]. They were 39,054

For peer review only - http://bmjopen.bmj.com/site/about/guidelines.xhtml

recorded operations within the UK National Bariatric Surgery Registry (NSBR) between 2013 to 2018. The Roux-en-Y gastric bypass was the most common bariatric surgical procedure (*n* 19,104, 48.9%), followed by sleeve gastrectomy (*n* 13,841, 35.4%) then the gastric band (*n* 4,499, 11.5%) [17].

Previous research suggests that people who undergo bariatric surgery have higher rates of pre-operative depression compared to people with obesity who do not undergo surgery [18]. A 2016 meta-analysis of the international literature estimated that up to 23% of patients have a mood disorder at the time of surgery [19], with the pooled estimate for depression being 19% (95% CI 14 to 25%, 34 studies, *N* 12,009/51,908 participants) and anxiety 12% (95% CI 6 to 20%, 22 studies, *N* 10,515/38,459 participants). In the short-term following surgery, there appears to be a reduction in the prevalence and severity of depression [20] however there remains uncertainty around the course of anxiety symptoms [20, 21, 22]. Previous literature on the mental health status of bariatric surgical recipients has often been limited due to the use of uncertain diagnostic criteria, measures for common mental disorders which do not address anxiety symptoms separately from depressive symptoms, and a lack of reporting on symptom severity [21]. As rates of severe and complex obesity increase, there is a clear need to better understand the prevalence and course of common mental health problems following surgery. This is particularly timely as recent research has found an increased risk of self-harm among those who undergo weight loss surgery [23, 24] compared to people with obesity who do not.

This paper presents findings from an analysis of data from the largest randomised controlled trial to date of bariatric surgery – the By-Band-Sleeve study [25, 26]. The

For peer review only - http://bmjopen.bmj.com/site/about/guidelines.xhtml

study is comparing the clinical and cost effectiveness of gastric banding (Band), laparoscopic gastric bypass (Bypass) or sleeve gastrectomy (Sleeve) which are the three most common surgical treatments for severe obesity. The objectives of this sub-study were to describe the prevalence, and severity, of anxiety and depressive symptoms among participants who underwent any type of bariatric surgery within 6 months of randomisation at baseline (pre-randomisation) and following surgery at 12 months post-randomisation.

For peer review only

For peer review only - http://bmjopen.bmj.com/site/about/guidelines.xhtml

METHOD

Participants

Participants were included in this sub-study if they had taken part in the By-Band-Sleeve study, had undergone surgery (irrespective of procedure type) within six months of randomisation, and had completed the Hospital Anxiety and Depression Scale after informed consent and before randomisation. By-Band-Sleeve study exclusion criteria included: previous gastric surgery for severe and complex obesity, previous abdominal surgery or gastro-intestinal conditions that precludes the surgical intervention, large abdominal ventral hernia or hiatus hernia >5cm, pregnancy, clinical conditions (such as Crohn's disease, liver cirrhosis and portal hypertension), known silicone allergy, or active participation in another interventional research study which may interfere with the By-Band-Sleeve study.

To understand the effect of surgery on mental health, participants were excluded if they had not undergone surgery within six months of randomisation. This cut-off of six months from enrolment was selected a priori in the event of participants waiting a prolonged time for surgery to take place (for example, due to the ongoing impact of the COVID-19 pandemic on elective surgery), which may have reduced the accuracy and relevance of baseline assessment of pre-operative mental health status. In total, 1,351 participants were randomised to the By-Band-Sleeve study and in this paper, we report on the mental health outcomes of the 758 eligible participants.

Primary measure

The Hospital Anxiety and Depression Scale (HADS) was completed at pre-randomisation (study enrolment or 'baseline') and at 12 months post-randomisation.

For peer review only - http://bmjopen.bmj.com/site/about/guidelines.xhtml

HADS is a 14-item questionnaire (7 questions for anxiety 'A' and 7 questions for depressive 'D' symptoms), which asks the participant to score each item between 0 to 3 based on their level of agreement. A sub-scale total score of less than 8 is considered normal, 8 to 10 suggestive of possible anxiety or depressive disorder, and a score greater than 11 is suggestive of a probable disorder [27]. Previous research has determined that a sub-scale score of $\geq$8 represents the optimal case cut-off for clinical anxiety and depressive disorders, in terms of the balance between sensitivity and specificity [28].

Secondary measures

Baseline characteristics and demographic data for participants were collected on study enrolment. These included Body Mass Index (BMI), Age, Sex, Ethnicity, Marital Status, Tobacco use, Employment Status, and Income Band. Time from randomisation to surgery and number of centres participating were described.

Statistical analysis

Analyses were undertaken using Stata Version 16. Returned HADS questionnaires were assessed for completion of the 7-item anxiety (HADS-A) and depression (HADS-D) subscales. Participants who fully completed either subscale had a total symptom score calculated. The proportions of participants who met case criteria for possible anxiety and depression (defined as HADS-A/D $\geq$8) were described alongside baseline sociodemographic variables. The median symptom score (and interquartile range) was calculated for participants who had completed a subscale at both baseline and 12 months post-randomisation. The Wilcoxon signed-rank test was used to assess the statistical significance of any change in median symptom

For peer review only - http://bmjopen.bmj.com/site/about/guidelines.xhtml

score. The change in proportions of participants with possible depression or anxiety at pre-randomisation compared to 12 months post-randomisation was calculated alongside 95% confidence intervals. McNemar's chi-squared test was used to compare paired prevalence of anxiety and depression at baseline and 12 months post-randomisation.

Missing data and loss to follow-up

A complete case analysis was undertaken in which participants with fully completed HADS-A or HADS-D questionnaire subscales were included in the analysis. The characteristics of participants who did not return completed questionnaires at 12 months post-randomisation was compared to returners with respect to baseline symptom scores, proportion of clinical cases, and sociodemographic variables. For categorical variables, cross-tabulation was used to compare the distribution of baseline characteristics by repeat subscale return status. Odds ratios (with 95% confidence intervals) for questionnaire return status were calculated using logistic regression for each categorical variable. For continuous variables, which were normally distributed, a two-sample t-test was used to compare whether the mean value (such as BMI, age, and time from randomisation to surgery) differed by return status.

Ethical approval

The By-Band-Sleeve study gained National Health Service (NHS) ethics approval from the Southwest Frenchay Research Ethics Committee (REC No: 11/SW/0248) in 2011. The study is sponsored by the University of Bristol and was granted Health Research Authority (HRA) Approval in 2017. The By-Band-Sleeve Study is

For peer review only - http://bmjopen.bmj.com/site/about/guidelines.xhtml

For peer review only

registered with the National Institutes of Health ClinicalTrials.gov database (NCT02841527) and ISRCTN registry (ISRCTN00786323).

Patient and public involvement

This sub-study features data obtained from participants who took part in the By-Band-Sleeve study. Patients and public were involved in By-Band-Sleeve Study throughout the design and conduct of the trial. Patient representatives on the Trial Management Group contributed towards the writing of this manuscript and are recognised as co-authors. The results of this sub-study will be disseminated through the By-Band-Sleeve Patient and Public Involvement Group and summarised, for a non-specialist audience, on the study (www.bybandsleevestudy.blogs.bristol.ac.uk) webpage following publication.

For peer review only

For peer review only - http://bmjopen.bmj.com/site/about/guidelines.xhtml

RESULTS

Seven hundred and fifty-eight By-Band-Sleeve study participants who had undergone surgery at the time of undertaking this work and who were within six months of randomisation were included [***Figure 1***]. Participants were recruited between January 2013 and September 2019 from 12 NHS surgical centres in England. Demographic characteristics by baseline (pre-randomisation) total HADS scores (normal, possible, probable disorder) are displayed in [***Table 1***]. At the point of randomisation, the mean age of participants was 47.8 (Standard Deviation, *SD* 10.6) years and the mean BMI was 46.3 (SD 6.7) kg/m^2. In total 570/758 (75.2%) participants were female.

Participant characteristics by baseline HADS scores

Of the 758 participants, 737 (97.2%) had returned baseline HADS questionnaires. For the subscales, baseline completion for the HADS-A was 94.5% (716/758) and 93.9% (712/758) for the HADS-D. The median symptom score for both baseline HADS-A and HADS-D was 7 (IQR 4 – 10). The proportion of individuals meeting case criteria for a possible, or probable, anxiety disorder was 46.1% (*n* 330/716, 95% CI 42.4 to 49.7%) and 48.2% (*n* 343/712, 95% CI 44.5 to 51.8%) for depression. Time from randomisation to surgery varied with a mean time of 92.1 (SD 44.4) days and was similar across the groups when stratified by baseline anxiety and depression status [***Table 1***].

For peer review only - http://bmjopen.bmj.com/site/about/guidelines.xhtml

Table 1: Demographic data by baseline HADS scores

		**HADS-A** (n = 716) Anxiety case category			**HADS-D** (n = 712) Depression case category		
		**Nil**	**Possible**	**Probable**	**Nil**	**Possible**	**Probable**
		<8	8 - 10	≥11	<8	8 - 10	≥11
**Total**	n (%)	386 (53.9)	159 (22.2)	171 (23.9)	369 (51.8)	181 (25.4)	162 (22.8)
**Time to surgery** (days)	mean (SD)	93.2 (45.3)	93.2 (44.3)	88.2 (41.5)	93.7 (46.5)	92.7 (43.8)	88.9 (39.7)
**Age** (years)	mean (SD)	48.1 (10.5)	46.8 (10.7)	46.3 (10.6)	47.8 (10.5)	47.7 (11.2)	46.4 (10.2)
**BMI** (kg/m^2)	mean (SD)	46.0 (6.4)	47.0 (7.1)	46.9 (7.0)	46.2 (6.6)	46.5 (6.9)	46.8 (7.0)
**Sex** n (%)	Male	102 (56.4)	42 (23.2)	37 (20.4)	90 (49.7)	50 (27.6)	41 (22.7)
	Female	284 (53.1)	117 (21.9)	134 (25.1)	279 (52.5)	131 (24.7)	121 (22.8)
**Ethnicity** n (%)	White	359 (53.9)	150 (22.5)	157 (23.6)	340 (51.4)	173 (26.1)	149 (22.5)
	African or Caribbean	17 (60.7)	6 (21.4)	5 (17.9)	17 (60.7)	4 (14.3)	7 (25.0)
	Mixed	7 (50.0)	2 (14.3)	5 (35.7)	8 (57.1)	1 (7.1)	5 (35.7)
	Asian	0 (0.0)	0 (0.0)	3 (100.0)	2 (66.7)	0 (0.0)	1 (33.3)
	Other	3 (60.0)	1 (20.0)	1 (20.0)	2 (40.0)	3 (60.0)	0 (0.0)
**Marital status** n (%)	Married or civil partnership	223 (55.8)	83 (20.8)	94 (23.5)	205 (52.0)	95 (24.1)	94 (23.9)
	Co-habiting	50 (58.8)	19 (22.4)	16 (18.8)	45 (52.9)	22 (25.9)	18 (21.2)
	Single	69 (51.5)	32 (23.9)	33 (24.6)	70 (52.2)	34 (25.4)	30 (22.4)
	Divorced	28 (43.1)	19 (29.2)	18 (27.7)	35 (52.2)	19 (28.4)	13 (19.4)
	Separated	11 (52.4)	5 (23.8)	5 (23.8)	11 (52.4)	7 (33.3)	3 (14.3)
	Widowed	5 (45.5)	1 (9.1)	5 (45.5)	3 (27.3)	4 (36.4)	4 (36.4)
**Smoking status** n (%)	Never smoked	181 (56.4)	61 (19.0)	79 (24.6)	156 (48.8)	83 (25.9)	81 (25.3)
	Ex-smoker	181 (52.0)	90 (25.9)	77 (22.1)	189 (54.8)	83 (24.1)	73 (21.2)
	Current smoker	24 (51.1)	8 (17.0)	15 (31.9)	24 (51.1)	15 (31.9)	8 (17.0)
**Employment status** n (%)	Employed	285 (62.1)	93 (20.3)	81 (17.7)	267 (58.8)	110 (24.2)	77 (17.0)
	Not in employment	62 (33.0)	50 (26.6)	76 (40.4)	70 (37.2)	51 (27.1)	67 (35.6)
	Student	4 (57.1)	2 (28.6)	1 (14.3)	4 (57.1)	2 (28.6)	1 (14.3)
	Retired	35 (56.5)	14 (22.6)	13 (21.0)	28 (44.4)	18 (28.6)	17 (27.0)
**Income band (GBP)** n (%)	≤ £10,000	28 (31.5)	29 (32.6)	32 (36.0)	35 (35.6)	30 (33.3)	28 (31.1)
	10,001-30,000	159 (52.5)	60 (20.6)	73 (25.0)	156 (54.0)	69 (23.9)	64 (22.2)
	30,001-50,000	89 (59.3)	36 (24.0)	25 (16.7)	80 (53.7)	41 (27.5)	28 (18.8)
	50,001-70,000	44 (68.8)	12 (18.8)	8 (12.5)	38 (60.3)	15 (23.8)	10 (15.9)

For peer review only - http://bmjopen.bmj.com/site/about/guidelines.xhtml

	> £70,001	18 (66.7)	6 (17.2)	3 (11.1)	20 (71.4)	6 (21.7)	2 (7.1)
	Not disclosed	47 (50.5)	16 (17.2)	30 (32.3)	42 (45.7)	20 (21.7)	30 (32.6)
	Missing	1 (100.0)	0 (0.0)	0 (0.0)	1 (100.0)	0 (0.0)	0 (0.0)

Prevalence of anxiety and depression at 12 months post-randomisation

At 12 months post-randomisation, nine of the participants who had completed baseline HADS-A and eight of the participants who had completed baseline HADS-D had withdrawn or died. [***Figure 1***]. After accounting for these individuals, the proportion of questionnaires returned complete was 71.1% (*n* 503/707) for the HADS-A and 70.7% (*n* 498/704) for the HADS-D. The median HADS score decreased from 7 at baseline to 5 (IQR 2 – 10) for anxiety and to 3 (IQR 1 – 7) for depression at 12 months post-randomisation among participants who completed questionnaires at both timepoints [***Table 2***]. There was a statistically significant ($p < 0.001$) decrease in both HADS-A and HADS-D scores [***Figure 2***]. This was coupled with a significant reduction in the proportion participants meeting caseness for anxiety (9.5% decrease, 95% CI -14.3 to -4.8%, $p < 0.001$) and depression (22.3% decrease, 95% CI -27.0 to -17.6%, $p < 0.001$) at 12 months post-randomisation when compared to baseline [***Figure 3***].

Whilst the overall proportion of cases of anxiety and depression decreased, the mental health of a small number of participants appeared to decline over the course of the 12-month follow-up, with 4.4% (*n* 22/498) of participants developing possible depression and 9.2% (*n* 46/503) developing a possible anxiety disorder [***Supplementary Table 1***].

For peer review only - http://bmjopen.bmj.com/site/about/guidelines.xhtml

Table 2: Change in HADS scores from baseline to 12 months post-randomisation						
				Proportion		
		**Median score** (IQR)	**Cases** (%)	**Change** (%)	**95% CI** (%)	**P-value**
**HADS-A** Anxiety (*n* 503)	Baseline	7 (4 – 10)	45.3	-9.5	-14.3 to -4.8	<0.001
	12 months post-randomisation	5 (2 – 10)	35.8			
**HADS-D** Depression (*n* 498)	Baseline	7 (4 – 10)	46.4	-22.3	-27.0 to -17.6	<0.001
	12 months post-randomisation	3 (1 – 7)	24.1			

Characteristics of 12-month post-randomisation HADS questionnaire returners and non-returners

The prevalence of baseline anxiety and depression was similar among those who did and did not return a completed questionnaire. Baseline BMI, participant sex, ethnicity, marital status, smoking status, and self-reported income were not associated with repeat HADS questionnaire return [***Supplementary Table 2, Supplementary Table 3, Supplementary Table 4***].

Factors associated with 12-month post-randomisation HADS return included participant age and employment status. Participants who returned completed anxiety or depression questionnaires were on average older (HADS-A: 4.1 years older, 95% CI 2.5 to 5.8, p < 0.001; HADS-D: 4.3 years older, 95% CI 2.7 to 6.0, p < 0.001) than participants who did not return completed questionnaires. Compared to individuals who were in employment, being retired at baseline was associated with an increased

For peer review only - http://bmjopen.bmj.com/site/about/guidelines.xhtml

odds of completed HADS-A (Odds Ratio (OR) 2.7, 95% CI 1.2 to 5.8, $p < 0.01$) and HADS-D (OR 2.4, 95% CI 1.1 to 5.0, $p < 0.05$) questionnaire return at 12 months post-randomisation.

DISCUSSION

In this study of the course of common mental health disorders in a population of randomised participants undergoing bariatric surgery, nearly half of the sample met criteria for possible or probable anxiety or depression on trial enrolment. Following surgery, substantial reductions in the proportion of participants with possible depression and anxiety were observed at 12 months post-randomisation. The greatest reduction was observed in symptoms of depression, where there was over a 20% decrease in prevalence. Whilst most participants reported an improvement in their mental health, over a third retained symptoms of an underlying anxiety disorder and a quarter of participants met criteria for a depressive disorder at 12 months post-randomisation.

Compared to previously published research utilising the HADS, we found higher a prevalence of pre-operative anxiety and depression in our study sample. Karlsson et al. described HADS scores amongst a consecutive sample of participants (*n* 655) who took part in the Swedish Obese Subjects (SOS) study and underwent bariatric surgery [29]. Using identical cut-off points to those used in our study, the prevalence of pre-operative anxiety was 34% and that of depression was 24% among those who were surgically treated. Whilst the mean age of their sample was comparable to ours, the mean BMI (41.9 SD 4.2 kg/m^2) was lower. The increased BMI among our sample may reflect the higher rate of adult obesity within the UK population,

For peer review only - http://bmjopen.bmj.com/site/about/guidelines.xhtml
For peer review only

alongside the substantially lower number of bariatric surgical procedures taking place in the UK compared to Sweden and other European countries [30]. This may also be linked to the higher levels of depression and anxiety in our sample. In a prospective study of people who underwent bariatric surgery (*n* 153) recruited from six surgical centres in Austria and Germany, Burgmer et al. found that 40.5% of the sample had depression (HADS-D $\geq$8) at baseline which decreased to 17.1% after one year following surgery [31]. Participants had a higher mean BMI (51.3 SD 8.4 kg/m^2) compared to those enrolled in this study. However, they did not find any significant changes in anxiety caseness which could have arisen due to the use of a higher (HADS-A $\geq$10) case cut-off score.

The significant reduction in depression prevalence and symptom severity observed over the first post-operative is in keeping with other studies which have utilised differing assessment criteria, such as the Beck Depression Inventory (BDI) or structured clinical interview [19]. In the Longitudinal Assessment of Bariatric Surgery series (LABS), a large multicentre cohort study of adults undergoing bariatric surgery in the USA, the authors found that LABS-2 surgery recipients (*N* 2,148) monitored over three years experienced the greatest reduction in mean BDI score between baseline and one-year post-operatively [32]. In their study, participants with pre-operative depressive symptoms (defined using a BDI score of $\geq$10) were significantly more likely, than those with minimal or no symptoms, to experience depressive symptoms on follow-up. Whilst the literature has predominantly studied the trajectory of depressive disorders, structured clinical interviews could offer greater insight into the pre-operative prevalence of anxiety disorders. In a sub-sample of LABS participants (*N* 199) interviewed before bariatric surgery, 18.1% (*n* 36) were found to

For peer review only - http://bmjopen.bmj.com/site/about/guidelines.xhtml

have a current anxiety disorder, with a specific phobia (11.1%, *n* 22) being the most common diagnosis [33].

Our study has several strengths. To our knowledge, it is the largest prospective study to assess the short-term effects of bariatric surgery on both anxiety and depressive symptoms in the UK. Participants were screened with a validated case-finding scale which has been shown to be reliable in detecting both disorders [34]. Whereas previous studies have often utilised single dimension instruments. Our large sample were recruited from 12 UK NHS surgical centres that are likely to be representative of the national population undergoing bariatric surgery, compared to those sampled from a single geographical site. We also report the effect size, with respect to change in prevalence of anxiety and depression (an important metric which has been missing from previously published studies in the field [19, 21]), alongside the pre-operative sociodemographic characteristics of questionnaire non-returners which could inform the delivery of future work and targeted interventions for this group. Our finding that repeat questionnaire returners were slightly older (compared to questionnaire non-returners at 12-month post-randomisation) is in keeping with the wider epidemiological literature regarding survey response rates in this age-group [35, 36] and likely linked to the increase in response among those who were retired.

There were also some important limitations. There was a significant questionnaire non-return rate of around 30% at 12 months post-randomisation. Whilst we did not find an association between having poorer mental health at baseline and questionnaire non-return, it is possible that individuals who did not return repeat

For peer review only - http://bmjopen.bmj.com/site/about/guidelines.xhtml

HADS questionnaires may have later developed anxiety or depressive disorders following randomisation. After completing the HADS, participants were not offered a formal structured clinical interview to confirm the diagnosis of an anxiety or depressive disorder (this was not part of the study protocol), hence we have referred to possible and probable cases in keeping with the limitations of this questionnaire. Future work would benefit from the use of structured clinical interviews which could offer insight into the presence of other co-morbid mental health disorders at the time of surgery. We have also not explored the change in BMI of participants returning or not returning questionnaires in follow up (which may have influenced questionnaire return status) because this primary outcome data remains confidential until analyses of the main trial is completed. The By-Band-Sleeve study remains in active follow-up, and it was not possible to compare the differences in symptom scores between surgical groups. As the purpose of this study was to describe the course of anxiety and depressive symptoms, irrespective of procedure type, the research team were not unblinded to participant's surgical intervention status. The study did not feature a non-surgical control group; therefore, we are unable to compare the natural trajectory of symptoms amongst people who did not undergo surgery over the same time-period. In terms of sociodemographic characteristics, the participants were predominantly female, identified as being from a White British ethnic background, and in employment at the time of study. It is therefore possible that our findings are not generalisable to the other groups undergoing bariatric surgery, particularly males and individuals from ethnic minorities. It is also plausible that responses to the pre-randomisation HADS questionnaires may have been influenced or affected by social desirability bias, particularly if participants incorrectly perceived that disclosure of their mental health difficulties was going to influence the likelihood of surgery.

For peer review only - http://bmjopen.bmj.com/site/about/guidelines.xhtml

Participants response to the baseline HADS questionnaires had no bearing on their treatment allocation status and their responses remained confidential.

The role of mental health stigma and marginalisation has been highlighted throughout qualitative research [37, 38] featuring surgery recipients and could contribute to the high prevalence of poor mental health within our sample. Previous work has demonstrated a disparity in gains within mental health-related quality of life (HRQoL) compared to physical HRQoL following bariatric surgery [39] that could prove important to understanding the short-term effects within our sample. In a study of LABS-3 participants, the presence of a pre-operative anxiety or affective disorder was associated with reduced improvements in mental HRQoL in the long-term following surgery and was independent of weight gain [40]. Recent research has found that increased physical activity following surgery was associated with a sustained improvement in both mental and physical HRQoL, alongside a reduction in depressive symptoms [41].

CONCLUSIONS

Our study highlights the very high prevalence of pre-operative psychological morbidity amongst people undergoing bariatric surgery for the treatment of severe or complex obesity in the UK. An improvement in symptoms of anxiety and depression was observed following surgery amongst participants who had returned completed questionnaires. Future work must be undertaken to understand the mechanisms underpinning these associations and whether these improvements were sustained in the long-term.

For peer review only - http://bmjopen.bmj.com/site/about/guidelines.xhtml

Author contributions

Corresponding author

Dr Jonathan Gibb

Centre for Academic Mental Health, Population Health Sciences

Bristol Medical School, University of Bristol

Oakfield Grove, Clifton, Bristol BS8 2BN

Jonathan.gibb@bristol.ac.uk

+44 (0)117 428 2489

Contributors

All authors within the By-Band-Sleeve Collaborating Group contributed to the drafting and revision of this final manuscript. Jonathan Gibb (Corresponding author) developed the study idea based on existing trial data (conceived and developed by the By-Band-Sleeve Trial Management Group), contributed to the statistical analysis plan, undertook data-analysis, wrote the first draft, and revised the final manuscript.

Collaborators

The By-Band-Sleeve Collaborating Group

Rob C Andrews PhD, Medical Research, University of Exeter Medical School, Exeter, UK;

John Bessent, Patient representative, By-Band Sleeve Trial management Group, UK;

Jane M Blazeby MD, NIHR Bristol Biomedical Research Centre Bristol Medical School, Population Health Sciences, University of Bristol, Bristol, UK;

For peer review only - http://bmjopen.bmj.com/site/about/guidelines.xhtml

James P Byrne MD, University Hospital Southampton NHS Foundation Trust, Southampton, UK;

Nicholas Carter MSc, Portsmouth Hospitals University NHS Trust, Portsmouth, UK;

Caroline Clay (Deceased), Patient representative, By-Band-Sleeve Trial Management Group, UK;

Jenny L Donovan PhD, Bristol Medical School, Population Health Sciences, University of Bristol, Bristol, UK;

Jonathan Gibb MBChB, Centre for Academic Mental Health, Population Health Sciences, Bristol Medical School, University of Bristol, UK;

Eleanor A Gidman PhD, Bristol Trials Centre, Bristol Medical School, University of Bristol, Bristol, UK;

Graziella Mazza PhD, Bristol Trials Centre, Bristol Medical School, University of Bristol, Bristol, UK;

Paul Moran MD, Centre for Academic Mental Health, Population Health Sciences, Bristol Medical School, University of Bristol, UK;

Mary O'Kane MSc, Dietetic Department, Leeds Teaching Hospitals NHS Trust, Leeds, UK;

Barnaby C Reeves PhD, Bristol Trials Centre, Bristol Medical School, University of Bristol, Bristol, UK;

Chris A Rogers PhD, Bristol Trials Centre, Bristol Medical School, University of Bristol, Bristol, UK;

Nicki Salter DipHE, Somerset NHS Foundation Trust, Somerset, UK;

Janice L Thompson PhD, School of Sport, Exercise & Rehabilitation Sciences, University of Birmingham, Birmingham, UK;

Richard Welbourn MD, Somerset NHS Foundation Trust, Somerset, UK;

For peer review only - http://bmjopen.bmj.com/site/about/guidelines.xhtml

Sarah Wordsworth PhD, Health Economics Research Centre, Nuffield Department of Population Health, University of Oxford, UK

Jane M Blazeby (By-Band-Sleeve Chief Investigator) developed the sub-study idea, contributed to design, contributed to the statistical analysis plan, and co-supervised the project;

Paul Moran developed the sub-study idea, contributed to design, contributed to the statistical analysis plan, and co-supervised the project.

Graziella Mazza contributed to the study design;

Eleanor A Gidman prepared the sub-study dataset and contributed to the statistical analysis plan;

Chris A Rogers contributed to the statistical analysis plan.

Acknowledgements

Independent data monitoring committee

Craig Ramsay (Chair), Health Services Research Unit, University of Aberdeen UK

Nick Finer, UCLH centre for weight loss, metabolic and endocrine surgery, London, UK

Torsten Olbers, Sahlgrenska University Hospital, Sweden

Trial Steering Committee

Julia Brown (Chair), Leeds Institute of Clinical Trials Research, Leeds UK

John Dixon, Baker IDI Heart and Diabetes Institute, Melbourne, Australia

Steve Morris, Department of Public Health and Primary Care, University of Cambridge

For peer review only

Jodie Smith, Patient representative

Michel Suter, Université de Lausanne, Switzerland

John Wilding, Clinical Sciences Centre, University Hospital Aintree, Liverpool, UK

Lead research nurses

Sally Abbott, Benita Adams, Alison Fletcher, Hassina Furreed, Hussain Gordon, Jennifer Henderson, Helen Horton, Tracey Lee, Amy Long, Melody MacGregor, Sarah Matthias, Maria Moon, Catherine Moriarty, Rosemary Mullett, Nicki Salter, Jill Townley

Research nurses and practitioners

Philippa Allison, Fiona Brogan, Katie Cook, Paul Corrigan, , Anne Daw, Naomi Dindol, Jacqueline Dingle, Eve Fletcher, Jeremy Gilbert, Ana Gill, Beth Greenslade, Andrew Guy, Madeleine Hawkes, Emma Holzer, Lianne Hufton, Lucy Johnstone, Jasmine Jose, Susan Kelly, Krishna Kholia, Jasmina Mandair, Claire Mason, Priya Mathew, Maxine Nixon, Madeleine Pappas, Mark Priestley, Tracey Robson, Jana Rojkova, Rachel Schranz, Barbara Watkins, Louise White

For peer review only

Bariatric surgeons

Ahmed Ahmed, Sanjay Agrawal, Sara Ajaz, Waleed Al-Khyatt, Sherif Awad, Altaf Awan, Shlok Balupuri, Ashok Bohra, James Byrne, Richard Byrom, Nicholas Carter, Michael Clarke, Allwyn Cota, Markos Daskalakis, Nick Davies, Simon Dexter, Ian Finlay, Jeremy Hayden, James Hopkins, Noah Howes, Khaleel Fareed, Sherif

For peer review only - http://bmjopen.bmj.com/site/about/guidelines.xhtml

Hakky, James Hewes, Neil Jennings, Jamie Kelly, Ben Knight, Yashwant Koak, Moorthy Krishna, Paul Leeder, John Loy, Brijesh Madhok, Kamal Mahawar, David Mahon, Matthew Mason, Samir Mehta, Rajwinder Nijjar, Hamish Noble, Alan Osborne, Dimitri Pournaras, Sanjay Purkayastha, Martin Richardson, Abeezar Sarela, Rishi Singhal, Peter Small, Shaw Somers, Paul Super, Christos Tsironis, Richard Welbourn

For peer review only

For peer review only - http://bmjopen.bmj.com/site/about/guidelines.xhtml

Declaration of interests

From the By-Band-Sleeve Collaborative Group:

James P Byrne MD, On the By-Band-Sleeve Trial Management Group, is on the medical advisory board for the company Oxford Medical Products. All other authors declare no competing interests.

From those acknowledged:

Sanjay Agrawal received a royalty from Springer Publishers for being Editor of the book – 'Obesity, Bariatric and Metabolic Surgery-A Practical Guide' in addition to honoraria for lectures given at national and international bariatric meetings; Sanjay Agrawal is also the Director of Bariatric and Metabolic Surgery UK: Not for Profit – Charity Company; Company No: 11729612, Registered in England & Wales. Sherif Awad receives honoraria for lectures delivered at bariatric meetings. Nick Finer is the Chair of the Trial Steering Committee for the iPREVENT study (NIHR funded EME Project:15/185/16 - Increase colonic propionate as a method of preventing weight gain in young adults). John Dixon previously served as a consultant for the company Reshape who own the LapBand.

Ethical approval

The By-band study gained National Health Service (NHS) ethics approval from the South West Frenchay Research Ethics Committee (REC No: 11/SW/0248) on the 6th December 2011 and on the 8th May 2015 the Ethics Committee granted ethical approval to adapt the study from a two group (By-Band) to a three group (By-Band-Sleeve) trial. REC approval applies to all NHS sites taking part in the study. The study is sponsored by the University of Bristol and it is the responsibility of the

For peer review only - http://bmjopen.bmj.com/site/about/guidelines.xhtml

sponsor to ensure that all the conditions of the study are complied with. In addition, By-Band-Sleeve study was processed under pre-Health Research Authority (HRA) Approval systems, the study was granted HRA approval on the 24th July 2017. The By-Band-Sleeve Study is registered with the National Institutes of Health ClinicalTrials.gov database (NCT02841527) and ISRCTN registry (ISRCTN00786323).

Funding statement

The By-Band-Sleeve study is funded by the United Kingdom National Institute for Health Research (NIHR) HTA programme (ref: 09/127/53). The views and opinions expressed are those of the authors and do not necessarily reflect those of the HTA programme, the NIHR, the UK NHS, or the Department of Health. The trial is being delivered in collaboration with Bristol Trials Centre, a UKCRC registered clinical trials unit which is in receipt of NIHR CTU support funding. This study was supported by the NIHR Biomedical Research Centre at University Hospitals Bristol and Weston NHS Foundation Trust and the University of Bristol. Jane M Blazeby MD is partially funded by the Bristol NIHR Biomedical research centre.

Data sharing statement

Data may be obtained from a third party and are not publicly available. Data will not be made available for sharing until after publication of the main results of the randomised trial. Thereafter, anonymised individual patient data will be made available for secondary research, conditional on assurance from the secondary researcher that the proposed use of the data is compliant with the MRC Policy on

For peer review only - http://bmjopen.bmj.com/site/about/guidelines.xhtml

Data Preservation and Sharing regarding scientific quality, ethical requirements, and value for money.

For peer review only

For peer review only - http://bmjopen.bmj.com/site/about/guidelines.xhtml

REFERENCES

[1] Dai H, Alsalhe TA, Chalghaf N, Riccò M, Bragazzi NL, Wu J. The global burden of disease attributable to high body mass index in 195 countries and territories, 1990-2017: An analysis of the Global Burden of Disease Study. PLoS Med. 2020;17(7):e1003198.

[2] Vos T, Lim SS, Abbafati C, Abbas KM, Abbasi M, Abbasifard M, et al. Global burden of 369 diseases and injuries in 204 countries and territories, 1990–2019: a systematic analysis for the Global Burden of Disease Study 2019. The Lancet. 2020;396(10258):1204-22.

[3] Santomauro DF, Mantilla Herrera AM, Shadid J, Zheng P, Ashbaugh C, Pigott DM, et al. Global prevalence and burden of depressive and anxiety disorders in 204 countries and territories in 2020 due to the COVID-19 pandemic. The Lancet. 2021;398(10312):1700-12.

[4] Luppino FS, Wit LMd, Bouvy PF, Stijnen T, Cuijpers P, Penninx BWJH, et al. Overweight, Obesity, and Depression: A Systematic Review and Meta-analysis of Longitudinal Studies. Arch. Gen. Psychiatry. 2021;67:220-9.

[5] Pan A, Sun Q, Czernichow S, Kivimaki M, Okereke OI, Lucas M, et al. Bidirectional association between depression and obesity in middle-aged and older women. Int J Obes (Lond). 2012;36(4):595-602.

[6] Jung SJ, Woo H-t, Cho S, Park K, Jeong S, Lee YJ, et al. Association between body size, weight change and depression: systematic review and meta-analysis. British Journal of Psychiatry. 2017;211(1):14-21.

[7] Gariepy G, Nitka D, Schmitz N. The association between obesity and anxiety disorders in the population: a systematic review and meta-analysis. International journal of obesity. 2010 Mar;34(3):407-19.

[8] de Wit L, Have Mt, Cuijpers P, de Graaf R. Body Mass Index and risk for onset of mood and anxiety disorders in the general population: Results from the Netherlands Mental Health Survey and Incidence Study-2 (NEMESIS-2). BMC Psychiatry. 2022;22(1):522. 2.

For peer review only - http://bmjopen.bmj.com/site/about/guidelines.xhtml

[9] F ulton S, Décarie-Spain L, Fioramonti X, Guiard B, Nakajima S. The menace of obesity to depression and anxiety prevalence. Trends in Endocrinology & Metabolism. 2022;33(1):18-35.

[10] McLaughlin AP, Nikkheslat N, Hastings C, Nettis MA, Kose M, Worrell C, et al. The influence of comorbid depression and overweight status on peripheral inflammation and cortisol levels. Psychological Medicine. 2022;52(14):3289-96.

[11] Borgland SL. Can treatment of obesity reduce depression or vice versa? J Psychiatry Neurosci. 2021;46(2):E313-e8.

[12] National Institute for Health and Care Excellence. Obesity: identification, assessment and management [Internet]. [London]: NICE; 2014 [updated 2022 Sept 08; cited 2022 Dec 04]. (Clinical guideline [CG189]). Available from: https://www.nice.org.uk/guidance/cg189

[13] Douglas IJ, Bhaskaran K, Batterham RL, Smeeth L. Bariatric Surgery in the United Kingdom: A Cohort Study of Weight Loss and Clinical Outcomes in Routine Clinical Care. PLoS Med. 2015;12(12):e1001925.

[14] Syn NL, Cummings DE, Wang LZ, Lin DJ, Zhao JJ, Loh M, et al. Association of metabolic-bariatric surgery with long-term survival in adults with and without diabetes: a one-stage meta-analysis of matched cohort and prospective controlled studies with 174;772 participants. Lancet. 2021;397(10287):1830-41.

[15] Gloy VL, Briel M, Bhatt DL, Kashyap SR, Schauer PR, Mingrone G, et al. Bariatric surgery versus non-surgical treatment for obesity: a systematic review and meta-analysis of randomised controlled trials. BMJ. 2013;347:f5934.

[16] Nguyen NT, Varela JE. Bariatric surgery for obesity and metabolic disorders: state of the art. Nat. Rev. Gastroenterol. Hepatol. 2016;14:160-9.

[17] National Bariatric Surgery Registry. The United Kingdom National Bariatric Surgery Registry 3rd Report. 2020. Available from: https://e-dendrite.com/Publishing/Reports/Bariatric/NBSR2020.pdf [Accessed 12 Apr 2023]

For peer review only - http://bmjopen.bmj.com/site/about/guidelines.xhtml

[18] Booth H, Khan O, Prevost AT, Reddy M, Charlton J, Gulliford MC. Impact of bariatric surgery on clinical depression. Interrupted time series study with matched controls. J. Affect. Disord. 2015;174:644-9.

[19] Dawes AJ, Maggard-Gibbons M, Maher AR, Booth MJ, Miake-Lye I, Beroes JM, et al. Mental Health Conditions Among Patients Seeking and Undergoing Bariatric Surgery: A Meta-analysis. JAMA. 2016;315:150-63.

[20] Fu R, Zhang Y, Yu K, Mao D, Su H. Bariatric surgery alleviates depression in obese patients: A systematic review and meta-analysis. Obesity Research & Clinical Practice. 2022;16(1):10-6.

[21] Gill H, Kang S, Lee Y, Rosenblat JD, Brietzke E, Zuckerman H, et al. The long-term effect of bariatric surgery on depression and anxiety. J. Affect. Disord. 2019;246:886-94.

[22] de Zwaan M, Enderle J, Wagner S, Mühlhans B, Ditzen B, Gefeller O, et al. Anxiety and depression in bariatric surgery patients: A prospective, follow-up study using structured clinical interviews. Journal of Affective Disorders. 2011;133(1):61-8.

[23] Neovius M, Bruze G, Jacobson P, Sjöholm K, Johansson K, Granath F, et al. Risk of suicide and non-fatal self-harm after bariatric surgery: results from two matched cohort studies. Lancet Diabetes Endocrinol. 2018;6:197-207.

[24] Backman O, Stockeld D, Rasmussen F, Näslund E, Marsk R. Alcohol and substance abuse, depression and suicide attempts after Roux-en-Y gastric bypass surgery. Br J Surg. 2016;103:1336-42.

[25] Rogers CA, Reeves BC, Byrne J, Donovan JL, Mazza G, Paramasivan S, et al. Adaptation of the By-Band randomised clinical trial to By-Band-Sleeve to include a new intervention and maintain relevance of the study to practice. Br J Surg. 2017;104:1207-14.

[26] Rogers CA, Welbourn R, Byrne J, Donovan JL, Reeves BC, Wordsworth S, et al. The By-Band study: gastric bypass or adjustable gastric band surgery to treat morbid obesity:

For peer review only

For peer review only - http://bmjopen.bmj.com/site/about/guidelines.xhtml

study protocol for a multi-centre randomised controlled trial with an internal pilot phase. Trials. 2014;15:53.

[27] Zigmond AS, Snaith RP. The hospital anxiety and depression scale. Acta Psychiatr Scand. 1983;67:361-70.

[28] Bjelland I, Dahl AA, Haug TT, Neckelmann D. The validity of the Hospital Anxiety and Depression Scale: An updated literature review. J. Psychosom. Res. 2002;52:69-77.

[29] Karlsson J, Taft C, Rydén A, Sjöström L, Sullivan M. Ten-year trends in health-related quality of life after surgical and conventional treatment for severe obesity: the SOS intervention study. Int J Obes (Lond). 2007;31:1248-61.

[30] Borisenko O, Colpan Z, Dillemans B, Funch-Jensen P, Hedenbro J, Ahmed AR. Clinical Indications, Utilization, and Funding of Bariatric Surgery in Europe. Obes Surg. 2015;25:1408-16.

[31] Burgmer R, Petersen I, Burgmer M, de Zwaan M, Wolf AM, Herpertz S. Psychological outcome two years after restrictive bariatric surgery. Obes Surg. 2007;17(6):785-91.

[32] Mitchell JE, King WC, Chen J-Y, Devlin MJ, Flum D, Garcia L, et al. Course of depressive symptoms and treatment in the longitudinal assessment of bariatric surgery (LABS-2) study. Obesity. 2014;22(8):1799-806.

[33] Mitchell JE, Selzer F, Kalarchian MA, Devlin MJ, Strain GW, Elder KA, et al. Psychopathology before surgery in the longitudinal assessment of bariatric surgery-3 (LABS-3) psychosocial study. Surg Obes Relat Dis. 2012;8(5):533-41.

[34] Hansson M, Chotai J, Nordstöm A, Bodlund O. Comparison of two self-rating scales to detect depression: HADS and PHQ-9. Br J Gen Pract. 2009;59(566):e283-8.

For peer review only - http://bmjopen.bmj.com/site/about/guidelines.xhtml

[35]. Robb KA, Gatting L, Wardle J. What impact do questionnaire length and monetary incentives have on mailed health psychology survey response? British Journal of Health Psychology. 2017;22(4):671-85.

[36]. Regine A, Martin Veel S, Paul KH, Gølin Finckenhagen G, Kjell T, Johny K, et al. Non-response in a cross-sectional study of respiratory health in Norway. BMJ Open. 2016;6(1):e009912.

[37] Homer CV, Tod AM, Thompson AR, Allmark P, Goyder E. Expectations and patients' experiences of obesity prior to bariatric surgery: a qualitative study. BMJ Open. 2016;6:e009389.

[38] Raves DM, Brewis A, Trainer S, Han S-Y, Wutich A. Bariatric Surgery Patients' Perceptions of Weight-Related Stigma in Healthcare Settings Impair Post-surgery Dietary Adherence. Front Psychol. 2016;7:1497-.

[39] Lindekilde N, Gladstone BP, Lübeck M, Nielsen J, Clausen L, Vach W, et al. The impact of bariatric surgery on quality of life: a systematic review and meta-analysis. Obes Rev. 2015;16:639-51.

[40] Kalarchian MA, King WC, Devlin MJ, Hinerman A, Marcus MD, Yanovski SZ, et al. Mental disorders and weight change in a prospective study of bariatric surgery patients: 7 years of follow-up. Surg Obes Relat Dis. 2019;15(5):739-48.

[41] King WC, Hinerman AS, White GE, Courcoulas AP, Belle SH. Associations Between Physical Activity and Changes in Depressive Symptoms and Health-related Quality of Life Across 7 Years After Roux-en-Y Gastric Bypass Surgery: A Multicenter Prospective Cohort Study. Ann Surg. 2022;276(6):e777-e83.

For peer review only - http://bmjopen.bmj.com/site/about/guidelines.xhtml

LEGENDS

TABLE 1: Demographic data by baseline HADS-A and HADS-D scores. Case categories based on the original HADS cut-offs proposed by Zigmond & Snaith, 1983.

TABLE 2: Frequency and percentage change in clinical cases for individuals who underwent surgery within 6 months of randomisation with completed questionnaires at both timepoints. p-value associated with change in case proportions obtained from McNemar's chi-squared test statistic.

FIGURE 1: Flow Diagram

Flow diagram representing questionnaire subscale completion for the HADS amongst the sample obtained from the By-Band-Sleeve study.

FIGURE 2: HADS Anxiety (HADS-A) and Depression (HADS-D)

Total symptom scores for participants who completed HADS-A or HADS-D subscales at baseline (pre-randomisation) and 12 months post-randomisation. The horizontal black line at the HADS Score of 8 on the y axis represents the cut-off for clinical cases. For both anxiety and depression, there was a significant ($p < 0.001$) decrease in median HADS score at 12 months post-randomisation**.**

FIGURE 3: Change in clinical cases

Proportion of possible clinical cases (HADS-A/D $\geq$8) at baseline and 12 months post-randomisation. Each bar represents the case prevalence (with associated 95% confidence interval) for anxiety and depression. At 12 months post-randomisation, there was a reduction in the proportion of individuals with anxiety (9.5% decrease, 95% CI -14.3 to -4.8%, $p < 0.001$) and depression (22.3% decrease, 95% CI -27.0 to -17.6%, $p < 0.001$).

For peer review only - http://bmjopen.bmj.com/site/about/guidelines.xhtml

For peer review only

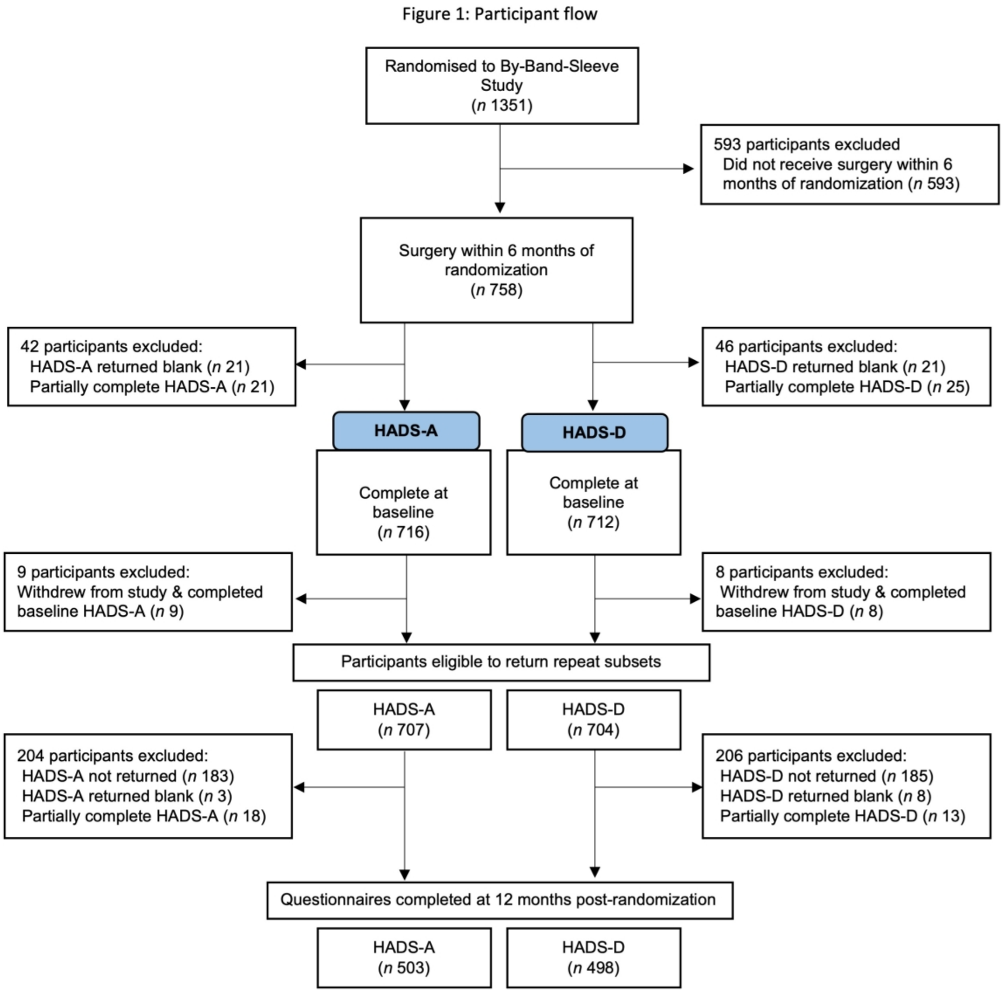

Flow diagram representing questionnaire subscale completion for the HADS amongst the sample obtained from the By-Band-Sleeve study.

192x192mm (330 x 330 DPI)

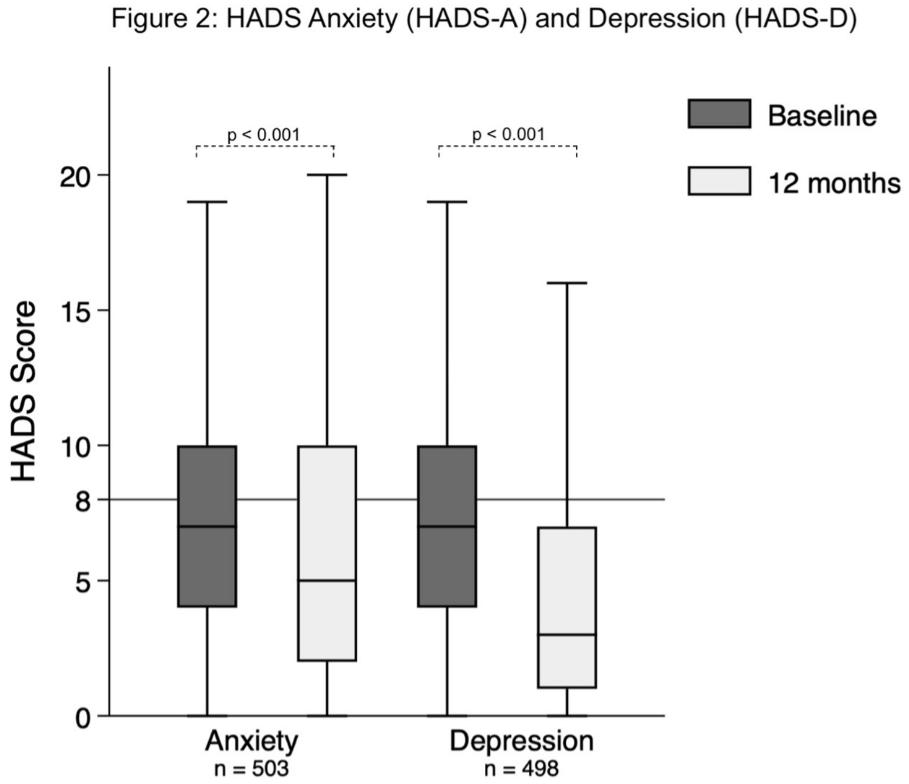

Total symptom scores for participants who completed HADS-A or HADS-D subscales at baseline (pre-randomisation) and 12 months post-randomisation. The horizontal black line at the HADS Score of 8 on the y axis represents the cut-off for clinical cases. For both anxiety and depression, there was a significant ($p < 0.001$) decrease in median HADS score at 12 months post-randomisation.

159x136mm (220 x 220 DPI)

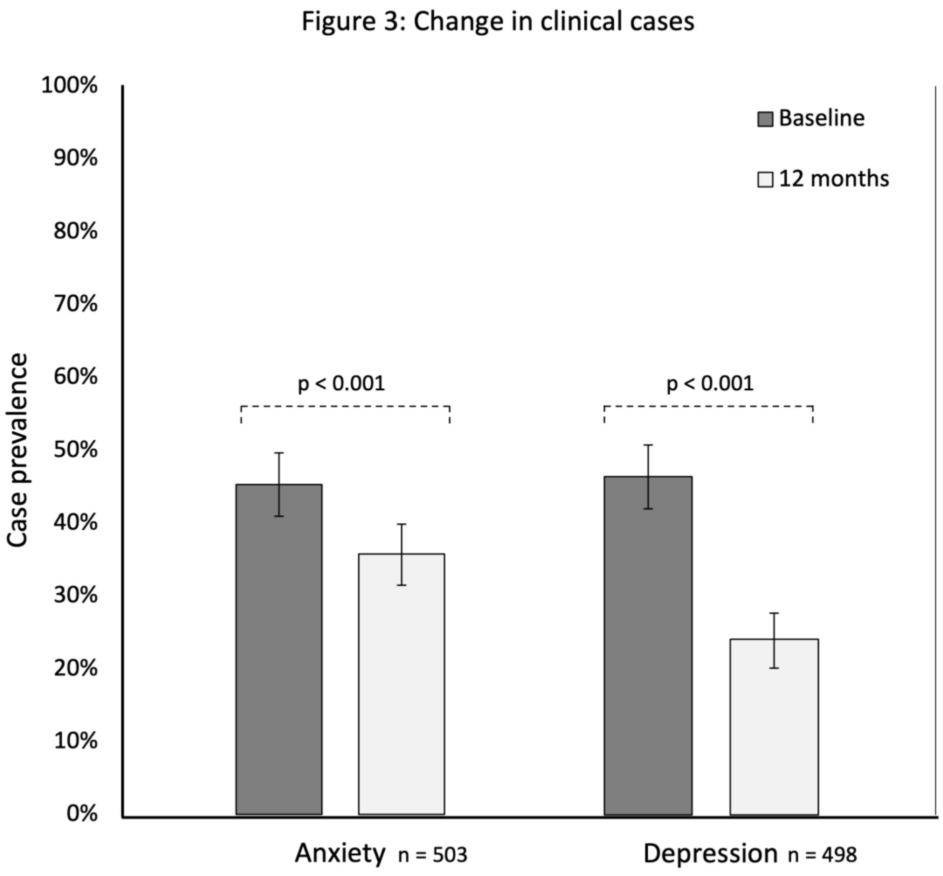

Proportion of possible clinical cases (HADS-A/D >8) at baseline and 12 months post-randomisation. Each bar represents the case prevalence (with associated 95% confidence interval) for anxiety and depression. At 12 months post-randomisation, there was a reduction in the proportion of individuals with anxiety (9.5% decrease, 95% CI -14.3 to -4.8%, $p < 0.001$) and depression (22.3% decrease, 95% CI -27.0 to -17.6%, $p < 0.001$).

165x149mm (330 x 330 DPI)

Supplementary Results

Anxiety and depression following bariatric surgery

List of supplementary tables

		Page
Table 1	Change in clinical cases from baseline to 12 months post-randomisation	2
Table 2	Baseline characteristics by repeat HADS questionnaire return status	3
Table 3	Baseline characteristics by repeat HADS-A questionnaire return status	4
Table 4	Baseline characteristics by repeat HADS-D questionnaire return status	5

For peer review only

For peer review only - http://bmjopen.bmj.com/site/about/guidelines.xhtml

Supplementary Results

Anxiety and depression following bariatric surgery

Table 1
Change in clinical cases from baseline to 12 months post-randomisation

		**Frequency**	**Proportion** (%)	**95% CI (%)**
**Anxiety** HADS-A (*n* 503)	Non-case unchanged	229	45.5	41.2 – 49.9
	Non-case to case	46	9.2	6.9 – 12.0
	Case to non-case	94	18.7	15.5 – 22.3
	Case unchanged	134	26.6	23.0 – 30.7
**Depression** HADS-D (*n* 498)	Non-case unchanged	245	49.2	44.8 – 53.6
	Non-case to case	22	4.4	2.9 – 6.6
	Case to non-case	133	26.7	23.0 – 30.8
	Case unchanged	98	19.7	16.4 – 23.4

For peer review only

For peer review only - http://bmjopen.bmj.com/site/about/guidelines.xhtml

Supplementary Results

Anxiety and depression following bariatric surgery

Table 2
Participant baseline characteristics by repeat HADS questionnaire return status
(Continuous variables)

		12 months post-randomisation HADS-A return			
		**Yes** (N = 503)	**No** (N = 204)	**Difference** (95% CI)	**p-value**[1]
**Age** (years)	Mean (95% CI)	48.7 (47.8 to 49.6)	44.6 (43.1 to 46.0)	4.1 (2.5 to 5.8)	<0.001
**BMI** (kg/m2)	Mean (95% CI)	46.3 (45.7 to 46.8)	46.8 (45.8 to 47.7)	-0.5 (-1.6 to 0.6)	0.379
**Time from randomisation to surgery** (days)	Mean (95% CI)	92.6 (88.7 to 96.5)	91.5 (85.5 to 97.6)	1.1 (-6.1 to 8.3)	0.767
		**12 months post-randomisation HADS-D return**			
		**Yes** (N = 498)	**No** (N = 206)	**Difference** (95% CI)	**p-value**[1]
**Age** (years)	Mean (95% CI)	48.8 (47.9 to 49.7)	44.5 (43.1 to 45.9)	4.3 (2.7 to 6.0)	<0.001
**BMI** (kg/m2)	Mean (95% CI)	46.3 (45.7 to 46.9)	46.8 (45.8 to 47.7)	-0.5 (-1.6 to 0.6)	0.370
**Time from randomisation to surgery** (days)	Mean (95% CI)	92.9 (89.0 to 96.8)	91.8 (85.8 to 97.8)	1.1 (-6.1 to 8.3)	0.767

[1] p-value obtained from paired sample t-test statistic for mean difference by HADS return status

For peer review only - http://bmjopen.bmj.com/site/about/guidelines.xhtml

Supplementary Results

Anxiety and depression following bariatric surgery

Table 3
Participant characteristics by repeat HADS-A questionnaire return status (categorical variables)

		12 months post-randomisation HADS-A return			
		**Yes** (%) N = 503 (71.15)	**No** (%) N = 204 (28.85)	**Odds Ratio for non-return**[2] (95% CI)	**p-value**[3]
**Sex**	Male (*n* 178)	126 (70.79)	52 (29.21)	1.00	0.903
	Female (*n* 529)	377 (71.27)	152 (28.73)	0.98 (0.67 to 1.42)	
**Ethnicity**	White (*n* 658)	472 (71.73)	186 (28.27)	1.00	0.217
	Other ethnic group[4] (*n* 49)	31 (63.27)	18 (36.73)	1.47 (0.80 to 2.70)	
**Marital status**	Married or civil partnership (*n* 395)	280 (70.89)	115 (29.11)	1.00	0.926
	Co-habiting (*n* 84)	62 (73.81)	22 (26.19)	0.86 (0.51 to 1.47)	
	Single (*n* 132)	92 (69.70)	40 (30.30)	1.06 (0.69 to 1.63)	
	Divorced, Separated, or Widowed[5] (*n* 96)	69 (71.88)	27 (28.12)	0.95 (0.58 to 1.56)	
**Smoking status**	Never smoked (*n* 317)	226 (71.29)	91 (28.71)	1.00	0.339
	Ex-smoker (*n* 343)	248 (72.30)	95 (27.70)	0.95 (0.68 to 1.33)	
	Current smoker (*n* 47)	29 (61.70)	18 (38.30)	1.54 (0.82 to 2.91)	
**Employment status**	Employed (*n* 455)	326 (71.65)	129 (28.35)	1.00	0.002
	Not in employment or student[6] (*n* 190)	123 (64.74)	67 (35.26)	1.38 (0.96 to 1.97)	
	Retired (*n* 62)	54 (87.10)	8 (12.90)	0.37 (0.17 to 0.81)	
**Income band**	$\leq$10,000 (*n* 84)	59 (70.24)	25 (29.76)	1.00	0.247
	10,001 to 30,000 (*n* 289)	212 (73.36)	77 (26.64)	0.86 (0.50 to 1.46)	
	30,001 to 50,000 (*n* 150)	105 (70.00)	45 (30.00)	1.01 (0.56 to 1.81)	
	$\geq$50,001 (*n* 91)	69 (75.82)	22 (24.18)	0.75 (0.38 to 1.47)	
	Not disclosed (*n* 92)	57 (61.96)	35 (38.04)	1.45 (0.77 to 2.72)	

2 Odds ratio for questionnaire non-return calculated using logistic regression
3 p-value obtained from likelihood ratio chi-square test
For calculation of Odds ratios associated with questionnaire non-return, categories with sub-groups containing $\leq$5% (*n* 35/707) total respondents were merged with the next largest sub-group to avoid data sparsity: **4** Includes participants who identified as African or Caribbean (*n* 28/707), Mixed ethnic group (*n* 13/707), Asian (*n* 3/707), or Other (*n* 5/707). **5** Participants who identified as separated (*n* 21/703) and widowed (*n* 11/703) combined with those who identified as divorced (*n* 64/703) as next largest category. **6** Participants who identified as students (*n* 6/703) combined with those not in employment (*n* 183/703)

For peer review only - http://bmjopen.bmj.com/site/about/guidelines.xhtml

Supplementary Results

Anxiety and depression following bariatric surgery

Table 4
Baseline characteristics by repeat HADS-D questionnaire return status (categorical variables)

		12 months post-randomisation HADS-D return			
		**Yes** (%) N = 498 (70.74)	**No** (%) N = 206 (29.26)	**Odds Ratio for non-return**[2] (95% CI)	**p-value**[3]
**Sex**	Male (*n* 178)	126 (70.79)	52 (29.21)	1.00	0.987
	Female (*n* 526)	372 (70.72)	154 (29.28)	0.98 (0.69 to 1.46)	
**Ethnicity**	White (*n* 655)	468 (71.45)	187 (28.55)	1.00	0.139
	Other ethnic group[4] (*n* 49)	30 (61.22)	19 (38.78)	1.59 (0.87 to 2.89)	
**Marital status**	Married or civil partnership (*n* 390)	273 (70.00)	117 (30.00)	1.00	0.592
	Co-habiting (*n* 84)	64 (76.19)	20 (23.81)	0.73 (0.42 to 1.26)	
	Single (*n* 132)	90 (68.18)	42 (31.82)	1.09 (0.71 to 1.67)	
	Divorced, Separated, or Widowed[5] (*n* 98)	71 (72.45)	27 (27.55)	0.89 (0.54 to 1.45)	
**Smoking status**	Never smoked (*n* 317)	225 (70.98)	92 (29.02)	1.00	0.566
	Ex-smoker (*n* 340)	243 (71.47)	97 (28.53)	0.98 (0.70 to 1.37)	
	Current smoker (*n* 47)	30 (63.83)	17 (36.17)	1.39 (0.73 to 2.63)	
**Employment status**	Employed (*n* 451)	322 (71.40)	129 (28.60)	1.00	0.003
	Not in employment or student[6] (*n* 190)	122 (64.21)	68 (35.79)	1.39 (0.97 to 1.99)	
	Retired (*n* 63)	54 (85.71)	9 (14.29)	0.40 (0.33 to 0.49)	
**Income band**	$\leq$10,000 (*n* 86)	59 (68.60)	27 (31.40)	1.00	0.108
	10,001 to 30,000 (*n* 286)	207 (72.38)	79 (27.62)	0.83 (0.49 to 1.41)	
	30,001 to 50,000 (*n* 149)	103 (69.13)	46 (30.87)	0.98 (0.55 to 1.73)	
	$\geq$50,001 (*n* 91)	72 (79.12)	19 (20.88)	0.58 (0.29 to 1.14)	
	Not disclosed (*n* 91)	56 (61.54)	35 (38.46)	1.37 (0.73 to 2.54)	

2 Odds ratio for questionnaire non-return obtained using logistic regression
3 P-value obtained from likelihood ratio chi-square test
For calculation of Odds ratios associated with questionnaire non-return, categories with sub-groups containing $\leq$5% (*n* 35/707) total respondents were merged into the next largest sub-group to avoid data sparsity: **4** Includes participants who identified as African or Caribbean (*n* 28/704), Mixed ethnic group (*n* 13/707), Asian (*n* 3/707), or Other (*n* 5/707). **5** Participants who identified as separated (*n* 21/703) and widowed (*n* 11/703) combined with those who identified as divorced (*n* 66/703) as next largest category. **6** Participants who identified as students (*n* 7/703) combined with those not in employment (*n* 183/703).

For peer review only - http://bmjopen.bmj.com/site/about/guidelines.xhtml

STROBE: Anxiety and depression following bariatric surgery

STROBE Statement

	Item No	Recommendation	Page No
**Title and abstract**	1	(*a*) Indicate the study's design with a commonly used term in the title or the abstract	1
		(*b*) Provide in the abstract an informative and balanced summary of what was done and what was found	4, 5
**Introduction**			
Background/rationale	2	Explain the scientific background and rationale for the investigation being reported	7, 8
Objectives	3	State specific objectives, including any prespecified hypotheses	4, 5, 8, 9
**Methods**			
Study design	4	Present key elements of study design early in the paper	8, 10
Setting	5	Describe the setting, locations, and relevant dates, including periods of recruitment, exposure, follow-up, and data collection	10, 11, 12, 13
Participants	6	(*a*) Give the eligibility criteria, and the sources and methods of selection of participants	10
Variables	7	Clearly define all outcomes, exposures, predictors, potential confounders, and effect modifiers. Give diagnostic criteria, if applicable	10, 11
Data sources/ measurement	8*	For each variable of interest, give sources of data and details of methods of assessment (measurement). Describe comparability of assessment methods if there is more than one group	10, 11, 12
Bias	9	Describe any efforts to address potential sources of bias	10, 11
Study size	10	Explain how the study size was arrived at	10
Quantitative variables	11	Explain how quantitative variables were handled in the analyses. If applicable, describe which groupings were chosen and why	11, 12
Statistical methods	12	(*a*) Describe all statistical methods, including those used to control for confounding	11, 12
		(*b*) Describe any methods used to examine subgroups and interactions	11, 12
		(*c*) Explain how missing data were addressed	11, 12
		(*d*) If applicable, describe analytical methods taking account of sampling strategy	11, 12
		(*e*) Describe any sensitivity analyses	11, 12
**Results**			
Participants	13*	(a) Report numbers of individuals at each stage of study—eg numbers potentially eligible, examined for eligibility, confirmed eligible, included in the study, completing follow-up, and analysed	14, 15, Figure 1 [Flow diagram]
		(b) Give reasons for non-participation at each stage	14, 15, Figure 1
		(c) Consider use of a flow diagram	Figure 1

For peer review only

For peer review only - http://bmjopen.bmj.com/site/about/guidelines.xhtml

STROBE: Anxiety and depression following bariatric surgery

Descriptive data	14*	(a) Give characteristics of study participants (eg demographic, clinical, social) and information on exposures and potential confounders	15 Table 1 (All) Appendix A (Non-responders)
		(b) Indicate number of participants with missing data for each variable of interest	15, 18, Table 1, Figure 1
Outcome data	15*	Report numbers of outcome events or summary measures	14, 15, 16, 17, 18 Table 2, Table 3, Figure 2, Figure 3, Appendix A
Main results	16	(*a*) Give unadjusted estimates and, if applicable, confounder-adjusted estimates and their precision (eg, 95% confidence interval). Make clear which confounders were adjusted for and why they were included	14, 17, Table 2
		(*b*) Report category boundaries when continuous variables were categorized	15, Table 1, Table 2, Table 3
		(*c*) If relevant, consider translating estimates of relative risk into absolute risk for a meaningful time period	N/A
Other analyses	17	Report other analyses done—eg analyses of subgroups and interactions, and sensitivity analyses	18, Appendix A
**Discussion**			
Key results	18	Summarise key results with reference to study objectives	19
Limitations	19	Discuss limitations of the study, taking into account sources of potential bias or imprecision. Discuss both direction and magnitude of any potential bias	20, 21
Interpretation	20	Give a cautious overall interpretation of results considering objectives, limitations, multiplicity of analyses, results from similar studies, and other relevant evidence	19, 20
Generalisability	21	Discuss the generalisability (external validity) of the study results	20
**Other information**			
Funding	22	Give the source of funding and the role of the funders for the present study and, if applicable, for the original study on which the present article is based	28

*Give information separately for exposed and unexposed groups.

Note: An Explanation and Elaboration article discusses each checklist item and gives methodological background and published examples of transparent reporting. The STROBE checklist is best used in conjunction with this article (freely available on the Web sites of PLoS Medicine at http://www.plosmedicine.org/, Annals of Internal Medicine at http://www.annals.org/, and Epidemiology at http://www.epidem.com/). Information on the STROBE Initiative is available at www.strobe-statement.org.

For peer review only

For peer review only - http://bmjopen.bmj.com/site/about/guidelines.xhtml
